# Supplementary material for: New infant formulas for healthy term infants: A randomized, controlled, double-blind, multicenter, non-inferiority design safety study
Source: PLoS One. 2025 Dec 17;20(12):e0336689. doi: 10.1371/journal.pone.0336689 (PMC12711075; doi:10.1371/journal.pone.0336689)
Supplement: S1 File — (DOCX) [file pone.0336689.s001.docx]

**Title:** New Infant Formulas for Healthy Term Infants: A Randomized, Controlled, Double-Blind, Multicenter, Non-inferiority Design Safety Study

**Authors:** Stephen A. Fleming^1^, Stefanie Flunkert^2^, Anne S. Kvistgaard^3^, James McGrath^4^, David K. Glover^5*^

^1^Traverse Science, 435 E Hawley St #816, Mundelein, IL, United States, 60060

^2^BioDoks e.U., 8410 St. Georgen an der Stiefing, Austria

^3^Arla Foods Ingredients Group P/S, Sønderhøj 10 -12, 8260 Viby J, Denmark

^4^Building Block Nutritionals, LLC, 200 Garrett Street, Suite S, Charlottesville, VA 22902

^5^PBM Capital Group, LLC, 200 Garrett Street, Suite S, Charlotteville, VA 22902

***** Corresponding Author: Traverse Science, 435 E Hawley Street #816, Mundelein, IL 60060

[Stephen@traversescience.com](mailto:Stephen@traversescience.com)

# Supplemental Material

# Multi-center controls and quality assurance

Each clinical site consented to study-related monitoring, audits, IRB review, and regulatory inspection by providing direct access to source data and documents. All data were recorded as case report forms (CRFs). Clinical sites were periodically monitored to 1) perform source document verification for all patients at each site, 2) review paper and electronic case report forms for accuracy, completeness of information, missing data, intercurrent illness, serious adverse events, missing patient visits or examinations, and omission of specifical individual data and concomitant drugs, and 3) verifying each sites investigator/designee reviewed each case report form. Standard operating procedures were followed for the collection of patient data, and all measurement equipment were inspected and approved by the CRO clinical monitor initial site visits. Further, clinical sites were included as a statistical covariate to address site-to-site variability.

# Stool composition

Fecal calcium was analyzed by modifying AOAC 984.27, 985.01, and 2011.14 and analyzed on a dry weight basis. Soap fatty acids were determined using methods previously described (24). Briefly, samples were thawed, homogenized, and lyophilized. Sample was extracted by solvent reflux and treated with acetic acid and soap fatty acids isolated by a second solvent reflux step. Non-soap (neutral) lipids and soap (acid-released) lipids were determined gravimetrically. Internal standard was added to both extracts, and free acids isolated from neutral lipids using solid phase extraction. Isolated free acids were converted to methyl esters using methanolic hydrochloric acid and analyzed via gas chromatograph equipped with a flame ionization detector, and quantitated using external standards. Only total soap fatty acids and soap palmitic acids were analyzed.

# Cytokine analysis

A single blood sample of at least 0.5 mL but not more than 0.8 mL was collected from each subject via heel stick at visit 9 to assess levels of the inflammatory markers tumor necrosis factor-α (TNF-α); interleukins 2, 4, 5, 6, 8, 10, 12, 13, and 17; interleukin-2 receptor; interleukin-1β, and interferon-γ. Before taking a blood sample, the heel was treated with a numbing cream followed by povidone-iodine for disinfection. After collection in a 1 mL red top capillary serum tube, the sample was thoroughly mixed by inverting the tube five times. Blood was then allowed to clot for 30 to 60 minutes and centrifuged at 1,500 to 2,000 x g for at least 15 minutes until the blood clot and serum were separated. Serum was then transferred to a fresh tube, frozen at -20°C, and shipped frozen on the collection day to Covance CLS (Indianapolis, IN, USA). ARUP Laboratories analyzed cytokines using a quantitative multiplex bead assay (CYT 12 SE; ARUP Laboratories, UT, USA).

# Associated Datafile

There are several sheets in the supplemental excel data file at <https://doi.org/10.5281/zenodo.16616829>. They contain the following data:

- Formula composition
  - Contains the full nutrient/ingredient composition of the formulas used.
- CM
  - Concomitant Medications in the Safety population
- MH
  - Medical History in the Safety population
- AE
  - Treatment-Emergent Adverse Events in the Safety population
- Stool composition
  - A read-friendly table containing data for fecal calcium, palmitic acid, and total soap fatty acids.
- GI Tolerance
  - A read-friendly table containing data on gassiness and fussiness
- Long-form outcomes
  - A code-friendly table containing data on anthropometrics, formula intake, gastrointestinal metrics, and ICQ data.
- Enrollment Age
  - A table listing the enrollment ages of each population.
