# Supplementary material for: New infant formulas for healthy term infants: A randomized, controlled, double-blind, multicenter, non-inferiority design safety study
Source: PLoS One. 2025 Dec 17;20(12):e0336689. doi: 10.1371/journal.pone.0336689 (PMC12711075; doi:10.1371/journal.pone.0336689)
Supplement: S1 Protocol — Trial Protocol. (PDF) [file pone.0336689.s003.pdf]

Growth and Safety Study of an Infant Formula for Healthy Term Infants

Date of Protocol: 09 August 2017

Acknowledgment of Responsibilities (Protocol Version 2)

This protocol is the property of Building Block Nutritionals, LLC. I understand that the information within it is confidential and is provided to me for review by myself, my staff, and applicable ethics committees. I understand that the protocol must be kept in a confidential manner and must be returned to the Sponsor, Building Block Nutritionals, LLC, or destroyed per Building Block Nutritionals, LLC instructions, upon request. No part of this protocol may be reproduced in any form without written authorization from Building Block Nutritionals, LLC. By accepting this protocol, I agree that the information contained herein will not be disclosed to a third party without written authorization from Building Block Nutritionals, LLC.

I have read and understood the protocol and agree that it contains all of the necessary information to carry out the study.

I agree to conduct this trial in accordance with all stipulations of the protocol and in accordance with the following: Good Clinical Practice, the ethical principles that have their origin in the Declaration of Helsinki; Title 21 of the Code of Federal Regulations, Parts 50 (Protection of Human Subjects), and 56 (Institutional Review Boards), and 312 (Investigational New Drug Application); and International Council for Harmonisation E6 (Guideline for Good Clinical Practice).

I agree that I will not modify this protocol without obtaining the prior approval of the sponsor and of the institutional review board or independent ethics committee, except when necessary to protect the safety, rights, or welfare of subjects.

Institution Name	Investigator Name	Signature	Date

TABLE OF CONTENTS

1.0 CONTACTS 7
1.1 Sponsor 7
1.2 Clinical Research Organization 7
2.0 ABBREVIATIONS 7
3.0 SYNOPSIS 8
4.0 ETHICS 10
4.1 Institutional Review Board 10
4.2 Ethical Conduct of Study 10
4.3 Informed Consent 10
5.0 FLOWCHART 11
5.1 Study Flowchart 11
6.0 BACKGROUND INFORMATION AND RATIONALE 11
6.1 Introduction 11
6.2 Alpha-lactalbumin 12
6.3 Lactoferrin 13
6.4 Osteopontin 14
6.5 OPO Sn-2 oil 15
6.6 Pre-biotics 16
6.7 Lutein 17
6.8 Microencapsulated DHA and ARA 17
7.0 OBJECTIVES 18
8.0 STUDY DESIGN 18
8.1 Approximate Duration of Subject Participation 18
8.2 Approximate Number of Subjects 18
9.0 SELECTION OF SUBJECTS 19
9.1 Inclusion Criteria 19
9.2 Exclusion Criteria 20

10.0 PRIOR AND CONCOMITANT MEDICATION AND TREATMENT 20

11.0 PROCEDURES 21

11.1 Screening....21

11.2 Study Visit Procedures....21

12.0 INVESTIGATIONAL PRODUCT AND ADMINISTRATION....23

12.1 Study Formulas 23

12.2 Type and Amount 24

12.3 Administration....24

12.4 Formula Storage by Investigator....24

12.5 Formula Storage by Parent/Guardian 24

12.6 Subject Compliance 25

13.0 SAFETY 25

14.0 EFFICACY....25

14.1 Primary Efficacy Endpoint....25

14.2 Secondary Efficacy Endpoints 25

15.0 LABORATORY DETERMINATIONS....25

15.1 Biological samples 25

16.0 STATISTICS....26

16.1 Statistical Methods....26

16.2 Statistical Power and Sample Size Considerations 27

17.0 SUBJECT IDENTIFICATION 28

18.0 INVESTIGATIONAL PRODUCT ACCOUNTABILITY 28

18.1 Formula Inventory....28

18.2 Formula Disposition....28

19.0 RANDOMIZATION 28

20.0 ADVERSE EVENTS 29

20.1 Definitions....29

21.0 SERIOUS ADVERSE EVENTS....30

22.0 ADVERSE EVENT AND SERIOUS ADVERSE EVENT RECORDING AND REPORTING....31

23.0 SUBJECT DISCONTINUATION OR WITHDRAWAL 31

24.0 INFORMED CONSENT 32

25.0 PROTOCOL AMENDMENTS 32

26.0 QUALITY CONTROL AND ASSURANCE 32

27.0 DIRECT ACCESS, DATA HANDLING, AND RECORD-KEEPING 33

28.0 RECORDS RETENTION 33

29.0 REFERENCES 35

30.0 APPENDICES 40

30.1 Appendix A: Anthropometry Data Collection Procedures 40

30.2 Appendix B: Formula Composition 44

30.3 Appendix C: Infant Formula Ingredients 46

30.4 Appendix D: Sample Investigational Product Labels 47

30.5 Appendix E: Investigational Product Accountability.......................................48

30.6 Appendix F: Standardized definitions for common Adverse Events that may or may not be related to formula tolerance (stooling, spit-up, crying, skin issues).........50

30.7 Appendix G: Site Investigational Product Accountability Log 52

30.8 Appendix H: Subject Investigational Product Accountability Log...................53

30.9 Appendix I: Infant Formula Preparation Instructions 54

1.0 CONTACTS

1.1 Sponsor

Building Block Nutritionals (BBN), LLC
200 Garrett Street, Suite S
Charlottesville, VA 22902

1.2 Clinical Research Organization

Paidion Research, Inc.
240 Leigh Farm Road, Suite 250
Durham, NC 27707

2.0 ABBREVIATIONS

Abbreviation	Term
AE	Adverse event
ARA	Arachidonic acid
BBN	Building Block Nutritionals, LLC
CFR	Code of Federal Regulations
CRF	Case report form
CSA	Clinical study agreement
CSR	Clinical study report
DHA	Docosahexaenoic acid
Formula B	Experimental Formula (Building Block Nutritionals, LLC)
Formula A	Commercially Available Milk Infant Formula (Enfamil Premium® 0-12 months)
FOS	Fructo-oligosaccharide
GCP	Good Clinical Practice
GI	Gastrointestinal
GOS	Galacto-oligosaccharide
GRAS	Generally recognized as safe
HM	Human milk
ICF	Informed Consent Form
IEC	Independent ethics committee
IP	Investigational product
IRB	Institutional Review Board
ITT	Intent to treat
PD	Polydextrose
PP	Per protocol
PWD	Powder
SAE	Serious adverse event

3.0 SYNOPSIS

This is a synopsis. The body of the protocol must be referenced for the complete study information.

**Study Title:**	Growth and Safety Study of an Infant Formula for Healthy Term Infants
**Rationale:**	A goal of infant formula development is to mimic human milk (HM) both in nutrient composition as well as physiologic outcomes. We have developed an infant formula for term infants that more closely resembles the composition of human milk. The purpose of this study is to demonstrate that this formulation meets nutritional requirements and supports age appropriate growth of healthy term infants.
**Objectives:**	The **primary efficacy objective** is to compare the growth of infants randomized to the experimental infant formula for term infants (Formula B) versus growth of infants randomized to a commercially available term infant formula (Formula A).<br><br>The **secondary efficacy objectives** are to describe among the formula groups:<br>a. Gastrointestinal tolerance (stool characteristics, stool composition, spit-up, gas)<br>b. Markers of inflammation (tumor necrosis factor-alpha, interleukin 2, 4, 5, 6, 8, 10, 12, 13, & 17; interleukin 2 receptor, interleukin 1 beta, and interferon gamma)<br><br>The **primary safety objective** is to compare the frequency of adverse events (AEs) between the formula groups (Formula A versus Formula B).
**Design:**	This study is a randomized, controlled, double-blind, study of healthy term formula fed (FF) infants. FF infants will be randomized to receive either a new infant formula formulated for healthy term infants (Formula B) or a commercially available infant formula for healthy term infants (Formula A). Infants will consume the formula for a total of 16-weeks; infant growth, serum markers of nutritional status, and tolerance to the formulas will be assessed throughout the study.
**Inclusion Criteria:**	Infants will be eligible to participate if they meet all of the following conditions. At birth the infant must be:<br>1. Healthy, term (early term/no less than 37 weeks, 0 days through late term/no greater than 41 weeks, 6 days), singleton infant<br>2. Have a birth weight of ≥ 2500 grams<br><br>At the time of the baseline/enrollment visit, infants must be:<br>3. Designated as healthy by a physician<br>4. ≤14 days post-natal age (Date of Birth = Day 0)<br>5. Weight for age ≥ 5th and ≤ 95th percentile for age according to sex-specific World Health Organization (WHO) growth charts

	6. Length for age ≥ 5th and ≤ 95th percentile for age according to sex-specific World Health Organization (WHO) growth charts<br>7. Head circumference for age ≥ 5th and ≤ 95th percentile for age according to sex-specific World Health Organization (WHO) growth charts<br>8. Weight for length for age ≥ 5th and ≤ 95th percentile for age according to sex-specific World Health Organization (WHO) growth charts<br>9. Exclusively consuming and tolerating a cow's milk infant formula at time of enrollment; only infants whose parent(s) or legal guardian(s) have decided to feed infant formula as the sole soure of nutrition, will be approached for potential study enrollment<br>10. Have parent(s) or legal guardian(s) who agree to feed the study formula to the study subject as his/her sole source of nutrition for the duration of the study<br>11. Have parent(s) or legal guardian(s) who have read and voluntarily signed an Informed Consent form approved by the Institutional Review Board prior to any participation in the study.
**Exclusion Criteria:**	Infants will be ineligible if they have any of the following conditions that are judged by a physician to interfere with the infant's normal growth, development, and/or tolerance to an infant formula:<br>1. Show evidence of anatomic and physiologic defects of the respiratory tract, or other congenital defects (as determined by the clinician);<br>2. Show evidence of chronic hepatic, gastrointestinal, renal, cardiac, pulmonary, or neurological diseases;<br>3. Have a maternal history with known adverse effects on the fetus and/or the newborn infant, such as diabetes (gestational diabetes is acceptable if infant's birth weight is < 4300 g), active tuberculosis, perinatal infection, or substance abuse<br>4. Have a family history of cow's milk protein intolerance/allergy<br>5. Are an infant from a multiple birth (twin, triplet, etc.)
**Investigational products and Administra-tion:**	Infants will consume ad libitum per day one of the following:<br>1. Formula B: An infant formula for term infants containing alpha-lactalbumin enriched whey, OPO Sn-2 oil, osteopontin, lactoferrin, pre-biotics (PD, FOS, GOS), lutein, microencapsulated DHA/ARA; 100 kcal/5 fl. oz, 2.2 g protein/100kcal (Manufactured by Building Block Nutritionals, LLC)<br>2. Formula A: A commercially available infant formula for term infants; 100 kcal/5 fl. oz, 2.0 g protein/100kcal (Enfamil Premium® 0-12 months by Mead Johnson Nutrition, LLC)
**Primary Efficacy and Safety Evaluations**:	Weight gain velocity (g/d)<br><br>Adverse Events will be collected throughout the 16-week study

**Statistics**	Sample size estimation is based on the primary endpoint, weight gain velocity (g/d) over a 16-week study period. Weight gain velocity will be compared between Formula groups A and B by the calculation of the 95% confidence interval on the difference between the two means. If the confidence interval does not include 3.0 g/d, the formulation will be declared equivalent. The number and percent of subjects with each AE will besummarized. All secondary endpoints will be descriptively summarized.<br><br>The criterion for equivalence in weight gain is that the difference in the two formula-fed groups' mean weight gain velocity (g/d) is significantly less than 3 g/d. Assuming a standard deviation in weight gain of 5.6 g/d (Nelson et al., 1989) and 80% power, 90 subjects per group (180 subjects total) will be sufficient to demonstrate equivalence (one sided alpha = 0.025). Assuming a 25% attrition rate, a total 256 subjects will be enrolled in this trial. Within each formula group, infants will be balanced by sex (equal number of males and females in each formula group).

4.0 ETHICS

4.1 Institutional Review Board

Clinical documentation for this study, including the clinical protocol, protocol amendments, and informed consent forms will be reviewed and approved by the IRB of each study site.

4.2 Ethical Conduct of Study

The study will be performed in accordance with the ethical principles that have their origin in the Declaration of Helsinki. The trial will be conducted in compliance with protocol, Good Clinical Practice, and other applicable regulatory requirements.

4.3 Informed Consent

Written informed consent will be obtained from the parent(s) or guardian(s) of each infant before any study formulas are fed to the study subject or data are collected, using the IRB-approved informed consent form.

5.0 FLOWCHART

Shaded columns indicate visit occurs at study site

5.1 Study Flowchart

**Study Visit**	**1**	**2**	**3**	**4**	**5**	**6**	**7**	**8**	**9**
**Study Day**	**0**	**15** ±3 days	**30** ±3 days	**45** ±3 days	**60** ±3 days	**75** ±3 days	**90** ±3 days	**105** ±3 days	**120** ±3 days
Informed consent	X								
Inclusion/Exclusion criteria	X								
Demography	X								
Randomization	X								
Infant feeding history	X								
Physical Exam	X								
Medical History	X	X	X		X		X		X
Anthropometry[1]	X	X	X		X		X		X
Stool Characteristics and Tolerance Questionnaire	X	X	X	X	X	X	X	X	X
Infant Characteristics Questionnaire (ICQ)	X	X	X		X		X		X
3-day Formula/Diet Record[2]	X	X	X		X		X		X
Blood Collection[3]									X
Dispense stool collection kit[4]							X		
Stool Collection[4]									X
Telephone contact[5]	X			X		X		X	
Concomitant Medications	X	X	X	X	X	X	X	X	X
Adverse events	X	X	X	X	X	X	X	X	X
Dispense and collect study formula	X	X	X		X		X		X

[1]Anthropometry includes assessment of weight, length, and head circumference (see Appendix C for procedure details).

[2]Initial 3-day Formula/Diet Record will be recorded for 3 days following the first study visit. Remainder of 3-day Formula/Diet Records will be recorded for 3 days prior to each subsequent scheduled study visit.

[3]Blood collection to assess markers of inflammation (tumor necrosis factor-alpha, interleukin 2, 4, 5, 6, 8, 10, 12, 13, & 17; interleukin 2 receptor, interleukin 1 beta, and interferon gamma) and albumin.

[4]Stool collection to assess stool composition. Stool collection supplies will be distributed to parents at Visit 7 for collection of all stools in the 3 days just before Visit 9. The stool collection kit is returned at Visit 9.

[5]Initial telephone contact will occur 3 days after enrollment to check compliance with study feeding and inquire about subject well-being. Remainder of telephone contacts will occur mid-way between clinic visits. Telephone contact notes will be recorded in the study subject's medical record.

6.0 BACKGROUND INFORMATION AND RATIONALE

6.1 Introduction

Human milk (HM) is universally considered the gold standard for infant feeding. HM is a dynamic, multi-faceted fluid containing nutrients and bioactive factors needed for infant health

and development. If an infant cannot be breastfed, the American Academy of Pediatrics recommends infant formula as the next best feeding alternative. A goal of infant formula development is to mimic HM in both nutrient composition as well as physiologic outcomes. For years, infant formulas have not been able to provide many of the bioactive factors found in HM. With a goal of providing infants who receive infant formula a nutritional product closer in composition to HM, improvements in infant formula composition are warranted. **The current study is designed to confirm that an infant formula for term infants that contains alpha-lactalbumin enriched whey, OPO Sn-2 oil, osteopontin, lactoferrin, prebiotics, lutein, microencapsulated DHA/ARA, designed to more closely match breast milk, will support growth in healthy, term infants.**

The proteins of human milk are divided into the whey and casein fractions, with each comprised of specific proteins and bioactive peptides that confer nutritional and functional benefits (Ballard and Morrow, 2013). The most abundant proteins are casein, alpha-lactalbumin, lactoferrin, secretory immunoglobulin IgA, lysozyme, serum albumin, and osteopontin (Ballard and Morrow, 2013; Schack et al 2009; Lonnerdal et al 2014).

6.2 Alpha-lactalbumin

Alpha-lactalbumin is the predominant whey protein found in HM with a concentration of 2 to 3 g/L (Jackson et al., 2004); however, it is a relatively minor component of bovine milk whey. Alpha-lactalbumin has both a biochemical role and a nutritional role for the mother-infant dyad. During lactation, the mammary gland produces alpha-lactalbumin and galactosyltransferase. These two proteins form the enzyme complex lactose synthase, which catalyzes the synthesis of lactose from glucose and galactose (Brodbeck et al., 1967). The nutritional value of alpha-lactalbumin lies in its high proportion of essential amino acids, specifically tryptophan, cysteine, and lysine (Lonnerdal, 1994). Standard term whey containing infant formulas typically provide some (~1.2g/L) bovine alpha-lactalbumin (Lien et al, 2004). Term infant formulas enriched with the whey protein, alpha-lactalbumin have been developed and are currently marketed today. Alpha-lactalbumin-rich formula retains a 60:40 whey to casein ratio and has an amino acid

profile closer to the amino acid profile of HM. The higher biological value of alpha-whey protein also allows for a reduction of total protein closer to that of HM (14 g protein/L alpha-lactalbumin rich formula versus 16 g protein/L in standard term infant formulas). Lien et al (2003) were the first to conduct a growth and safety study comparing the alpha-lactalbumin-rich formula to standard term infant formula. This study found that all parameters of growth for both groups were within age appropriate reference ranges, growth velocity was comparable between groups, and the alpha-lactalbumin-rich formula was well tolerated. Formula with alpha-lactalbumin enriched way have been marketed for over a decade and several other studies have documented the safety and suitability of infant formulas containing this whey (Trabulsi et al., 2011, DuPont et al., 2010). Alpha lactalbumin enriched whey protein concentrates used in this study are allowed to be used in infant formula in European Union (EU- no legal defintion), China (GB11674-2010), acceptable under Codex standard (289-1995) and also conforms to US legislation (CFR.21.184.1979c).

6.3 Lactoferrin

Lactoferrin is an iron-binding protein that is found in human milk. It plays a role in iron homeostasis, gastrointestinal defense against microbial infection, and has anti-inflammatory and immune modulatory effects (Lonnerdal 2014). It is found in the highest concentrations in colostrum, but persists throughout the entire first year. Cow's milk, which is used to make standard infant formulas, contains small amounts of bovine lactoferrin (bLF; 30– 485 mg/L), but for the most part, unless added, do not contain appreciable amounts of bLF (Cheng et al., 2008). Human and bovine lactoferrin share 70% sequence homology and have been shown to have similar biologic effects (Ochoa et al., 2008). The safety and efficacy of infant formula supplemented with 850mg/L bLF (King et al., 2007) was evaluated in small (n=52) 12- month placebo-controlled trial of healthy term infants. This study found that infants receiving formula supplemented with lactoferrin tolerated the formula, had significantly lower respiratory illness and higher blood hematocrit concentrations compared to infants fed standard formula (King et al., 2007). More recently a large- scale growth and safety study of infant formula supplemented with bLF was conducted. A total of 480 infants were enrolled and randomized to receive one of

three formulas: 1) standard cow milk formula (control), 2) an investigational formula with bLf at 600mg/L, or 3) an investigational formula with 1000mg lactoferrin/L, for the first year of life. The investigational formulas also contained a pre-biotic blend of blend of polydextrose (PDX) and galactooligosaccharides (GOS). This study found that all formulas were well tolerated and there were no group differences in growth rate (g/d) from 14-365 days of age among the study groups. Infants receiving the experimental formulas had softer stools from day 30 to day 180 (Johnston et al., 2015). A study of preterm infant formula supplemented with lactoferrin also reported the formula was well tolerated, supported age appropriate growth, and resulted in decreased fungal infections (Manzoni et al., 2012). Taken together, these studies demonstrate the safety and efficacy of lactoferrin supplemented infant formula.

6.4 Osteopontin

Osteopontin (OPN) is a bioactive protein found in human and bovine milk; it is thought to play a role in immunity, wound healing, and bone remodeling (Lonnerdal, 2014). The concentration of OPN in HM averages 138mg/L (Schack et al., 2009) and cow's milk typically contains 1/10 the concentrations. Standard infant formulas contain 5.3 – 13.0mg/L bovine osteopontin (Schack et al., 2009). OPN is a key cytokine in the regulation of the Th1/Th2 balanced immune response (Ashkar et al., 2000), protecting infants against infections by inducing a Th1 response. Indeed, breast-fed infants have been found to have greater induction of a Th1-like response after immunization against measles, mumps, and rubella, compared to formula fed infants (Pabst et al., 1997). A randomized clinical trial was recently conducted to evaluate effects of adding a bovine OPN fraction to formula. Formula fed infants were randomized to one of three formula groups: standard formula, (F0), standard formula with bovine OPN at 65 mg/L bovine OPN (F65) or standard formula with 130 mg/L bovine OPN (F130) mg/L (50% and 100% of human milk level, respectively) from 1 to 6 months of age. Growth and biological outcomes of formula fed infants were compared with a reference group of breast-fed (BF) infants. Among the formula-fed groups, the pro-inflammatory cytokine TNF-α was significantly lower in the F65 and F130 groups than in the F0 group, suggesting that OPN downregulates inflammatory cytokines in formula fed infants (Lonnerdal et al., 2016). There were no differences in growth,

formula intake, or tolerance among the formula fed groups (Lonnerdal et al., 2016). In addition, several fold higher levels of no observed adverse event levels (NOAEL) for bovine osteopontin have been established using in vitro, and in vivo animal studies (Kvistgaard et al 2014)

6.5 OPO Sn-2 oil

It has long been recognized that there are differences in bowel habits and gastrointestinal (GI) symptoms in HM fed versus formula fed (FF) infants. Infants fed HM are reported to have more frequent and softer stools than FF infants. The fat blends used in infant formulas may contribute to harder and less frequent stools reported in some FF infants. Human milk fat is characterized by high contents of palmitic and oleic acids, the former heavily concentrated in the Sn-2 position on the triglyceride molecule and the latter in the 1- and 3-positions of the triglyceride molecule (Lien, 1994). Fat absorption is higher from human milk than from current infant formulas, despite the similarities between the fatty acid profiles (Innis et al., 1994). This may partly be explained by the unique triacylglycerol structure of human milk. Palmitic acid (16:0) is abundant in human milk and is an important source of energy. Most of the 16:0 in human milk is located in the Sn-2 position of the triacylglycerol molecules, in contrast to cow's milk and vegetable oils which have 40 % and 5-20 %, respectively, of the 16:0 in the sn2-position. As such, hydrolysis of human milk fat result in 16:0 mainly as sn2-monoacylglycerol, which are well absorbed. Whereas hydrolysis of fat in infant formula 16:0 from vegetable oils will be present as free fatty acids, which tend to bind calcium and form insoluble calcium-soaps in the intestine that may cause harder stools and constipation Lien, 1994).

OPO Sn-2 oil is a structured triglyceride where palmitic acid bonded to the middle position (sn-2) of the glycerol backbone and oleic acid (18:1n-9) is bonded to the Sn 1 and 3 positions. OPO Sn-2 oil is an FDA GRAS approved oil the OPO oil in a proprietary oil blend that contains Coconut, Soybean and High Oleic Sunflower Oil. Several studies have evaluated the safety and efficacy of infant formula containing Sn-2 oils. Kennedy et al evaluated the efficacy of a term infant formula with a higher proportion of palmitate in the sn-2 position and found that compared to standard formula, the sn-2 enriched formula resulted in reduced stool soap fatty acids, softer

stools more like those of breast-fed, and higher whole body bone mineral content (Kennedy et al., 1999). Yao et al evaluated the efficacy of a formula with sn-2 oil and formulas with sn-2 oil plus oligofructose versus standard infant formula, and found that formula with sn-2 led to reduced stool calcium-soaps, softer stools; the addition of oligofructose further improved stool consistency (Yao et al., 2014). Other studies have reported that infants fed high sn-2 oil formulas compared to standard formula has reduced crying duration and frequency (Litmanovitz et al., 2014).

6.6 Pre-biotics

Human milk contains an abundance of complex glycans (saccharides), that are produced by the mammary gland. Human milk oligosaccharides (HMOs) are a family of glycans that include: glycoproteins, glycopeptides, and glycolipids (Smilowitz et al., 2014). These glycans are not digested and instead reach the large intestine intact, where they support the growth of good bacteria, enhance the intestine epithelial barrier, and bind bacteria, viruses, and toxins. Colostrum contains as much as 20–25 g/L HMO (Coppa et al., 1999) and mature milk contains 5–20 g/L HMO (Coppa et al., 1999; Davidson et al., 2004). HMOs are the third largest component in human milk after lactose and lipids and are present at >100 fold than are found in bovine milk (Zirkovic et al., 2011, Bode L. 2012). Due to limited availability and lack of technological advancements, alternative ingredients (prebiotics) were explored to match the functionality of HMOs. Prebiotics provide similar functional benefits as HMOs and are defined as “a selectively fermented ingredient that allows specific changes, both in the composition and/or activity in the gastrointestinal microbiota that confers benefits upon host well-being and health” (Roberfroid, 2007). Over the past decade, infant formulas with different combinations of added prebiotics have been clinically tested. These studies have found that: a mixture of galacto- and fructooligosaccharides added to term infant formula stimulated the growth of Bifidobacteria and Lactobacilli was well tolerated and resulted in softer stools (Moro et al., 2002); a formula with polydextrose (PDX) and galactooligosaccharides (GOS) and a formula with polydextrose (PDX), galactooligosaccharides (GOS), and lactulose (LOS), supported normal growth and resulted in softer stools (Ziegler et al., 2007); a term infant formula with polydextrose (PDX) and

galacto-oligosaccharides (GOS) increased total bifidobacteria and resulted in softer stools (Scalabrin et al., 2012). Taken together, these studies demonstrate that infant formulas with added pre-biotics are well tolerated by infants and support age appropriate growth.

6.7 Lutein

Carotenoids are yellow, orange, and red pigments synthesized by plants and include the compounds α-carotene, β-carotene, β-cryptoxanthin, lutein, zeaxanthin, and lycopene. Carotenoids function as antioxidants (Krinsky and Johnson, 2005), lutein is selectively taken up into the macula of the eye, where it absorbs potentially damaging blue light and helps maintain visual function (Landrum and Bone, 2001). Carotenoids cannot be synthesized in the body and therefore must be consumed in the diet. Human milk contains lutein from the mother's diet in ranges from 3mcg/L to 232 mcg/L (Canfield et al., 2003) 2 whereas infant formula contains much smaller and more variable amounts from trace ingredients. The safety of lutein supplement infant formula was tested in a 16 week study (Capeding et al., 2010). A total of 232 infants were randomized to one of two formulas: term infant formula or term infant formula with 200 mcg/L lutein. The lutein supplemented formula supported age appropriate growth and there were no significant differences in growth/weight gain or formula intake between the two groups (Capeding et al., 2010).

6.8 Microencapsulated DHA and ARA

Long chain polyunstaturated fatty acids (LCPFUAs) such as docosahexaenoic acid (DHA) and arachidonic aid (AA) play important roles in human biology, specifically in lipid-rich membranes of the central nervous system such as those in the eye and brain (Clandinin et al., 1980, Clandinin et al., 1980, Fleiser et al., 1983). A lack of sufficient LCPUFA intake may modify the growth and function of the central nervous system (Bourre et al., 1989, Neuringer M et al., 1989). Human milk provides the breast fed infant with DHA and ARA at mean (±SD) concentrations of $0.32 \pm 0.22\%$ and $0.47 \pm 0.13\%$ by weight for DHA and ARA, respectively (Brenna et al 2007). Infants fed formula without added LCPUFAs have been shown to have significantly lower plasma or red blood cell concentrations of DHA and AA compared to

breastfed infants or infants fed formulas supplemented with DHA/ARA (Austead et al., 1997, Birch et al., 2000). The safety of DHA/ARA supplemented infant formulas has been established in several randomized, double-blinded, placebo-controlled trials evaluating the efficacy of these LCPUFAs on infant cognitive function (Birch et al., 2000, Agostoni et al., 1995, O'Connor et al., 2001, Makrides et al., 2000).

7.0 OBJECTIVES

Primary Efficacy Objective:

- Compare the growth of infants randomized to the experimental infant formula for term infants (Formula B) versus growth of infants randomized to a commercially available term infant formula (Formula A).

Secondary Efficacy Objectives:

- To describe among the formula groups:
  - Gastrointestinal tolerance (stool characteristics, stool composition, fussiness, gas)
  - Markers of inflammation (tumor necrosis factor-alpha, interleukin 2, 4, 5, 6, 8, 10, 12, 13, & 17; interleukin 2 receptor, interleukin 1 beta, and interferon gamma).

Primary Safety objective

- To compare the frequency of adverse events (AEs) between the formula groups (Formula A versus Formula B).

8.0 STUDY DESIGN

8.1 Approximate Duration of Subject Participation

Subjects will participate in the study for 4 months.

8.2 Approximate Number of Subjects

Approximately 256 healthy term infants (128 per group, 64 per gender per group) will be enrolled to complete a minimum of 180 evaluable infants (90 per group, 45 per gender per group).

9.0 SELECTION OF SUBJECTS

The institutional review board (IRB) will review and approve the protocol and informed consent form for this study. Each parent/guardian must participate in the informed consent process and sign and date the current IRB-approved informed consent form for this protocol before any protocol-required procedures are performed.

9.1 Inclusion Criteria

Infants will be eligible to participate if they meet all of the following conditions.
At birth the infant must be:

1. Healthy, term (no less than 37 weeks, 0 days and no greater than 42 weeks, 0 days), singleton infant
2. Have a birth weight of ≥ 2500 grams

At the time of the baseline/enrollment visit, infants must be:

3. Designated as healthy by a physician
4. ≤14 days post-natal age (Date of Birth = Day 0)
5. Weight for age ≥ 5th and ≤ 95th percentile for age according to World Health Organization (WHO) growth charts
6. Length for age ≥ 5th and ≤ 95th percentile for age according to World Health Organization (WHO) growth charts
7. Head circumference for age ≥ 5th and ≤ 95th percentile for age according to World Health Organization (WHO) growth charts
8. Weight for length for age ≥ 5th and ≤ 95th percentile for age according to World Health Organization (WHO) growth charts
9. Exclusively consuming and tolerating a cow's milk infant formula at time of enrollment; only infants whose parent(s) or legal guardian(s) have decided to feed infant formula as sole soure of nutrition, will be approached for potential study enrollment
10. Have parent(s) or legal guardian(s) who agree to feed the study formula as the sole source of nutrition for the duration of the study
11. Have parent(s) or legal guardian(s) who have read and voluntarily signed an Informed Consent form approved by the Institutional Review Board prior to any participation in the study.

9.2 Exclusion Criteria

Infants will be ineligible if they have any of the following conditions that are judged by a physician to interfere with the infant's normal growth, development, and/or tolerance to an infant formula:

1. Show evidence of anatomic and physiologic defects of the respiratory tract, or other congenital defects (as determined by the clinician);
2. Show evidence of chronic hepatic, gastrointestinal, renal, cardiac, pulmonary, or neurological diseases;
3. Have a maternal history with known adverse effects on the fetus and/or the newborn infant, such as diabetes (gestational diabetes is acceptable if infant's birth weight is < 4300 g), active tuberculosis, perinatal infection, or substance abuse
4. Have a family history of cow's milk protein intolerance/allergy
5. Are an infant from a multiple birth (twin, triplet, etc.)

10.0 PRIOR AND CONCOMITANT MEDICATION AND TREATMENT

Infants previously breast-fed can be enrolled provided they have discontinued breastfeeding at study entry, and parents agree that they have voluntarily chosen to exclusively formula-feed the study subject during the study. If formula fed, infants must be currently receiving and tolerating cow's milk formula. At each study visit, site personnel will interview parent(s)/guardian(s) to obtain information about all concomitant therapy that was administered since the previous study visit. Concomitant medications include prescription medications, over-the-counter medications, and herbal supplements. This information will be recorded in the subject's medical record. If the infant was administered medication or treatment for a condition that may have an effect on the infants' growth and formula tolerance, the investigator or medical staff must assess the potential medical implications to determine whether the study subject remains eligible to continue in the study.

If a study subject was fed a formula other than the assigned study formula, this variation from protocol must be noted on the Protocol Deviation Log.

11.0 PROCEDURES

11.1 Screening

Each infant will be screened for all inclusion and exclusion criteria. If an infant complies with all inclusion and exclusion criteria and the parent(s)/guardian(s) sign the IRB-approved ICF, the infant will be randomized to receive a study formula and assigned a unique subject number. If the infant does not comply with one or more of the inclusion or exclusion criteria, the infant will be defined as a screen failure.

11.2 Study Visit Procedures

Visit 1

- Informed consent process
- Inclusion/Exclusion criteria
- Demography
- Randomization
- Infant feeding history
- Physical Exam
- Medical History
- Anthropometry (see Appendix A for Anthropometry Data Collection Procedures)
- Stool Characteristics and Tolerance Questionnaire
- Infant Characteristics Questionnaire
- 3-day Formula/Diet Record
- Telephone contact
- Medications
- Adverse events
- Dispensing of study formula

Visit 2

- Medical History
- Anthropometry
- Stool Characteristics and Tolerance Questionnaire
- Infant Characteristics Questionnaire
- 3-day Formula/Diet Record

- Medication
- Adverse events
- Dispensing and collecting of study formula

Visit 3

- Medical History
- Anthropometry
- Stool Characteristics and Tolerance Questionnaire
- Infant Characteristics Questionnaire
- 3-day Formula/Diet Record
- Medication
- Adverse events
- Dispensing and collecting of study formula

Visit 4: Telephone contact

- Medication
- Adverse events

Visit 5

- Medical History
- Anthropometry
- Stool Characteristics and Tolerance Questionnaire
- Infant Characteristics Questionnaire
- 3-day Formula/Diet Record
- Medication
- Adverse event
- Dispensing and collecting of study formula

Visit 6: Telephone contact

- Medication
- Adverse events

Visit 7

- Medical History
- Anthropometry

- Stool Characteristics and Tolerance Questionnaire
- Infant Characteristics Questionnaire
- 3-day Formula/Diet Record
- Medication
- Adverse events
- Dispensing and collecting of study formula

Visit 8: Telephone contact

- Medication
- Adverse events

Visit 9

- Medical History
- Anthropometry
- Stool Characteristics and Tolerance Questionnaire
- Infant Characteristics Questionnaire
- 3-day Formula/Diet Record
- Blood Collection
- Stool Collection
- Medication
- Adverse events
- Collection of all unused/partially used study formula

12.0 INVESTIGATIONAL PRODUCT AND ADMINISTRATION

12.1 Study Formulas

Infants will be randomized to one of two formulas:

1. Formula B: An infant formula for term infants containing alpha-lactalbumin enriched whey, OPO Sn-2 oil, osteopontin, lactoferrin, pre-biotics (PD, GOS, FOS), lutein, microencapsulated DHA/ARA; 100 kcal/5 fl. oz, 2.2 g protein/100kcal (Manufactured by Building Block Nutritionals, LLC)
2. Formula A: A commercially available infant formula for term infants; 100 kcal/5 fl. oz, 2.0 g protein/100kcal (Enfamil Premium® 0-12 months by Mead Johnson Nutrition, LLC)

See Appendix B for details of formula nutrient composition and Appendix C for formula ingredients.

12.2 Type and Amount

Each infant will consume either Formula B or Formula A ab libitum for 16 weeks. Parent(s) or guardian(s) will be discouraged from feeding their infant any foods other than the assigned study formula.

12.3 Administration

The study formula will be provided in powder form. Each of the infant formulas will be packaged in composite cans. Cans will be labeled with a unique clinical product number to mask the identity of each clinical product. Study personnel will not be aware of the identity of the products. Nutrient and stability testing will be conducted to ensure that each formula meets strict quality requirements for release. Instructions for mixing the formula to 20kcal/oz will be given to the parent(s)/guardian(s).

12.4 Formula Storage by Investigator

Formula will be labeled for clinical trial use only and kept dry, protected from sunlight, and at room temperature (60-85° F, 16-29° C). The principal investigator is responsible for keeping all unassigned and returned formula in a locked storage room with controlled staff access.

12.5 Formula Storage by Parent/Guardian

The parent(s)/guardian(s) will be instructed to handle formula with clean hands. Prior to opening, the can should be cleaned. Formula may be consumed at room temperature. The parent(s)/guardian(s) will be instructed to never microwave or freeze formula. Prepared formula may be warmed in a bowl of warm water. Parents will be instructed that prepared formula not consumed by the infant can be stored in the refrigerator for no more than 24 hours, and then should be thrown away.

Parents will be instructed to return all unopened cans of formula at the completion of the study. See Appendix D for sample investigational product label.

12.6 Subject Compliance

Compliance with study feedings will be monitored approximately every two weeks throughout the study. A standard set of interview questions will be administered at each clinic visit and during telephone follow-up between clinic visits to inquire about consumption of study formula and all other feedings including other formulas.

13.0 SAFETY

Safety assessments will involve the monitoring and recording of all AEs and serious adverse events (SAEs), laboratory results, and periodic anthropometric measurements and physical assessments. Additional safety evaluations may be performed when medically indicated in the opinion of the investigator.

14.0 EFFICACY

14.1 Primary Efficacy Endpoint

Growth rate (g/d) over 16 weeks.

14.2 Secondary Efficacy Endpoints:

Gastrointestinal tolerance (stool characteristics, stool composition, spit-up, gas)

Markers of inflammation (tumor necrosis factor-alpha, interleukin 2, 4, 5, 6, 8, 10, 12, 13, & 17; interleukin 2 receptor, interleukin 1 beta, and interferon gamma)

15.0 LABORATORY DETERMINATIONS

Central laboratories will be used for all laboratory determinations unless a special test or emergency testing is required. The central laboratories that have been contracted to perform these tasks adhere to Good Clinical Practices and will provide supplies and shipping materials for all laboratory determinations. Refer to the central laboratory manual(s) for additional information.

15.1 Biological samples

A single blood sample (of at least 0.5 mililiters but not more than 0.8 milliliters) will be collected from each subject via heel stick at the last study visit (visit 9) to assess markers of inflammation (tumor necrosis factor-alpha, interleukin 2, 4, 5, 6, 8, 10, 12, 13, & 17; interleukin 2 receptor,

interleukin 1 beta, and interferon gamma). Stool samples will also be collected at the last study visit (visit 9) to assess stool composition (soap fatty acids).

Biological samples will be used exclusively for the purposes outlined in this protocol and for no other purpose.

16.0 STATISTICS

16.1 Statistical Methods

16.1.1 Primary Efficacy Endpoint

The primary efficacy endpoint, mean daily weight gain (g/d) over a 16-week study period, will be compared between Formula groups A and B by the calculation of the 95% two-sided confidence interval on the difference between the two means. If the confidence interval does not include 3.0 g/d the formulations will be declared equivalent.

Infant growth will also be descriptively summarized on the basis of the following sex-specific z-scores based on World Health Organization growth charts: weight-for age, weight-for-length, length-for-age, and head circumference-for-age z-scores. For each subject, a line listing of all raw measures of weight, length, head circumference, and all z-scores (weight-for-age z-score, length-for-age z-score, weight-for-length z-score, and head circumference-for-age z-score) will be provided. Descriptive statistics will be used to summarize weight (kg), length (cm), and head circumference (cm) and all z-score data (weight-for age, weight-for-length, length-for-age, and head circumference-for-age) by formula group for each visit.

16.1.2 Adverse Events

A line listing of each subject with an adverse event will be generated. At the group level, the number and percentage of subjects having each AE will be summarized for each formula group.

16.1.3 Secondary Endpoints

Secondary endpoints including formula intake volume, stool characteristics and GI tolerance and infant characteristics, will be summarized using descriptive statistics and qualitatively compared to age appropriate reference values when appropriate.

16.1.4 General Methods

Continuous variables will be summarized using the appropriate descriptive statistics: n, mean, standard deviation, median, minimum, and maximum. The geometric mean will be presented for log-transformed variables. Frequency and percentage of observed values will be reported for categorical measures. A line listing of all data, sorted by subject and when appropriate by time, will be generated.

Analysis Populations

- Intent to Treat (ITT): Subjects with a Randomization Number who consumed at least one feeding of the assigned formula
- Per-Protocol (PP): A subset of the ITT population. It will consist of all subjects who complete the feeding protocol with no more than 3 complete days of non-study formula feeding, without major protocol violations. Subjects who consume a single non-study feeding more than 10 times during the duration of the 16 week study, will also be excluded from the PP population.
- Safety Population: The safety population will be comprised of any subjects who are randomized.

Classification into ITT or PP populations will be conducted prior to the database lock. All listings will be provided for the ITT population. A separate listing will be included on the set of subjects who are randomized but never take any feeding formula before discontinuing from the study.

Since this study has a primary aim at demonstrating equivalence, the PP population will represent the primary analysis population to evaluate the treatment groups in terms of "efficacy". All clinical outcomes (primary and secondary) will be subject to analyses using both the PP and ITT population.

16.2 Statistical Power and Sample Size Considerations

Assuming a standard deviation in weight gain of 5.6 g/d (Nelson et al., 1989) and 80% power, 90 subjects per group (180 total) will be sufficient to demonstrate equivalence (one sided alpha = 0.025). Assuming a 25% attrition rate, a total of 256 subjects will be enrolled in this trial. Within each formula group, infants will be balanced by sex (equal number of males and females in each

formula group). At the conclusion of the study, a post-hoc power analysis on weight gain will be conducted.

17.0 SUBJECT IDENTIFICATION

Subjects will be randomized and assigned a unique subject number. A subject number will never be reassigned or reused for any reason. The investigator will maintain a master log linking the subject number to the subject's name. The investigator will follow all applicable privacy laws in order to protect a subject's privacy and confidentiality. Information that could identify a subject will be masked on study material.

18.0 INVESTIGATIONAL PRODUCT ACCOUNTABILITY

18.1 Formula Inventory

The study site will maintain an Accountability Record of all formula received, dispensed, returned, or otherwise disposed of during the study. All dispensed and unused cans of study formula as well as empty or partially empty cans of study formula must be returned by the study subject's parent(s)/guardian(s) to the Investigator at each study visit. See Appendix E for details on investigational product accountability.

18.2 Formula Disposition

If an infant discontinues participation in the study, the remaining formula will be returned to the study site. All investigational study formula returned will be disposed of in accordance with the instructions provided by the Sponsor.

19.0 RANDOMIZATION

Formula fed infants will be randomly allocated to one of the two study formulas (Formula A or B) via IWRS (Interactive Web-Based Randomization System). Randomization will be blocked by formula group and stratified by infant sex to allow for an equal number of males and females in each formula group.

20.0 ADVERSE EVENTS

20.1 Definitions

An **adverse event** (AE) is any untoward, undesired, or unplanned event in the form of signs, symptoms, disease, or laboratory or physiologic observations occurring in a person administered an investigational product in a clinical study. The event does not need to be causally related to an investigational product or participation in the clinical study. Any illness that a study subject develops during the study must be recorded on the AE case report form. Whenever possible, an illness should be recorded as a diagnosis rather than a series of symptoms.

Standardized definitions for stooling, spit-up, and crying (agreed upon by the investigators and medical monitor; see Appendix F) will be used to report such symptoms.

All AEs must be assigned one of the following intensity scores:

- Mild: Transient or mild discomfort (<48 hours); no medical intervention/therapy required
- Moderate: Mild to moderate limitation in activity, some assistance may be needed; no or minimal medical intervention/therapy required
- Severe: Marked limitation in activity, some assistance usually required; medical intervention/therapy required, hospitalization possible
- Serious: please see section 21.0.

All AEs must be assigned causality. The relationship of each AE to the investigational product must be recorded on a binary scale, answering yes or no to the following question: “Is there a reasonable possibility of a causal relationship between investigational product and the AE?”

- Yes: There is a reasonable causal relationship to the investigational product, (i.e., related)
- No: There is not a reasonable causal relationship to the investigational product

As a guideline for determining if an adverse event is related to the investigational product (**investigational product related)**, the following questions should be considered:

- Does a reasonable causal relationship exist between the AE and the investigational product based on clinical judgment and knowledge of the treatment?
- Is there a temporal relationship between the investigational product and the appearance of the AE?

- Is there biologic plausibility for a relationship between the AE and the investigational product?
- Does the subject have an underlying medical condition or is the subject taking concomitant therapies or medications that could contribute to the AE?
- Where applicable, does the AE abate on discontinuation of the investigational product (dechallenge)?
- Where applicable, does the AE reappear on repeat exposure to the investigational product (rechallenge)?

A **protocol-related adverse event** is an AE occurring during a clinical study that is not related to the investigational product, but is considered by the investigator or the medical monitor (or designee) to be related to the research conditions, (i.e., related to the fact that a subject is participating in the study).

21.0 SERIOUS ADVERSE EVENTS

A serious adverse event (SAE) is defined as an AE that:

- Results in death
- Is life-threatening, ie, the subject was, in the opinion of the investigator, at immediate risk of death from the event as it occurred (it does not include an event that, had it occurred in a more severe form, might have caused death)
- Results in a significant, persistent or permanent change, impairment, damage, or disruption in the subject's body function/structure, physical activities, and/or quality of life
- Requires in-subject hospitalization or prolongs hospitalization
- Is another medically significant event that, based upon appropriate medical judgment, may jeopardize the subject and may require medical or surgical intervention to prevent one of the outcomes listed above (eg, allergic bronchospasm requiring intensive treatment in an emergency department or home, blood dyscrasias, or convulsions that do not result in hospitalization, or the development of drug dependency or drug abuse).

22.0 ADVERSE EVENT AND SERIOUS ADVERSE EVENT RECORDING AND REPORTING

Determination of AEs should be based on the signs or symptoms detected during the physical examination and on clinical evaluation of the subject. Adverse events (AEs) will be coded using the Medical Dictionary for Drug Regulatory Affairs (MedDRA).

AEs and SAEs will be collected from the signing of the informed consent form to the end of study visit 9. The investigator will instruct the subject to report AEs and SAEs during this time period.

During the time period specified above, the investigator will:

- Record all AEs and SAEs in the subject’s medical record.
- Record all AEs, SAEs, and the treatment of the AE/SAE on a case report form.
- Report all SAEs to the study sponsor as directed and to the IRB as appropriate.

An AE/SAE’s causal relationship to the product has no bearing on its reportability. The investigator must follow up on all AEs and SAEs until the events have subsided, until values have returned to baseline, or, in case of permanent impairment, until the condition has stabilized to a level acceptable to the sponsor/investigator.

All SAEs and follow-up information must be reported to the Study Sponsor and clinical research organization (CRO) medical monitor within 24 hours after learning of the event and per instructions as included in the Investigator Site File.

A listing of AEs and SAEs by subject will be provided in the clinical study report (CSR).

23.0 SUBJECT DISCONTINUATION OR WITHDRAWAL

A study subject may withdraw or discontinue the study at any time and for any reason. Reasons why a subject may discontinue or be withdrawn from the study include, but are not limited to, AE, parent(s)/guardian(s) request, investigator request, etc. When a subject discontinues or is withdrawn from the study, the investigator will complete all procedures designated for Visit 9 and notify the sponsor.

24.0 INFORMED CONSENT

The informed consent Form (ICF) used during the informed consent process must be the current IRB approved ICF.

Before any protocol-required procedures are performed, the parent(s)/guardian(s) must:

- Be informed of all aspects of the study.
- Be given time to ask questions and time to consider the decision to participate.
- Voluntarily agree to participate in the study.
- Sign and date an IRB/-approved informed consent form.

25.0 PROTOCOL AMENDMENTS

Any change in the study protocol requires a protocol amendment. An investigator must not make any changes to the study protocol without IRB and sponsor. All protocol amendments must be reviewed and approved by the IRB following the same process as the original protocol.
This study will be conducted as described in this protocol, except for an emergency situation in which the protection, safety, and well-being of the subject requires immediate intervention, based on the judgment of the investigator (or a responsible, appropriately trained professional designated by the investigator). In the event of a significant deviation from the protocol due to an emergency, accident, or mistake, the investigator or designee must contact the Sponsor, or their agent, at the earliest possible time by telephone. This will allow an early joint decision regarding the subject's continuation in the study. The investigator and the Sponsor will document this decision. The IRB/IEC will be informed of all protocol changes by the investigator in accordance with the IRB/EC established procedure. No deviations from the protocol of any type will be made without complying with all the IRB established procedures. Any and all protocol deviations will be reported to the IRB.

26.0 QUALITY CONTROL AND ASSURANCE

The sponsor or it's agent performs quality control and assurance checks on all clinical studies that it sponsors. Before enrolling any subjects in this study, the sponsor or it's agent and the investigator will review the protocol, the eCRF and eCRF instructions, the procedure for obtaining informed consent, all protocol procedures, and the procedure for reporting AEs and

SAEs. The sponsor or it's agent will monitor the conduct of the study on a periodic basis. During these site visits, information recorded in the eCRF is verified against source documents.

27.0 DIRECT ACCESS, DATA HANDLING, AND RECORD-KEEPING

The investigator will permit study-related monitoring, audits, IRB review, and regulatory inspections by providing direct access to source data and documents. All information will be recorded on source documents. All required data will be recorded in the case report forms (CRFs). All CRF data must be submitted to the sponsor throughout and at the end of the study. Electronic data capture will be used to record and transmit data electronically to the sponsor. The CRF data are stored in a database and processed electronically. The sponsor medical monitor reviews the data for safety information. The data are reviewed for legibility, completeness, and logical consistency. Automated validation programs identify missing data, out-of-range data, and other data inconsistencies. Requests for data clarification are forwarded to the investigative site for resolution.

Periodic monitoring visits will include a review of patient records, eCRFs, and other study related documentation. While on site, the monitor will be responsible for the following activities:

- performing source document verification (SDV) for 100% of patients at each site
- reviewing eCRFs for accuracy and completeness of information, missing data including omission of specific individual data elements and any concomitant drugs, intercurrent illness, serious adverse events, missing patient visits or examinations
- verifying (via a signature) the investigator or an appropriate designee's review of the eCRFs.

28.0 RECORDS RETENTION

The investigator shall retain and preserve 1 copy of all data collected or databases generated in the course of the study, specifically including but not limited to those defined by Good Clinical Practice (GCP) as essential, for two years after completion of the study. At the end of such period, the investigator shall notify the sponsor in writing of his or her intent to destroy all such material. The sponsor shall have 30 days to respond to the investigator's notice, and the sponsor shall have a further opportunity to retain such materials at the sponsor's expense. The sponsor will provide the FDA with 1 electronic copy of all data.

Subject's medical files should be retained in accordance with applicable legislation and in accordance with the maximum period of time permitted by the hospital, institution or private practice. The documents can be retained for a longer period, however, if required by the applicable regulatory requirements or by agreement with the Sponsor.
To comply with these requirements, the investigator will not dispose of any records relevant to this study without either (1) written permission from the Sponsor, or (2) providing an opportunity for the Sponsor to collect such records. The investigator shall take responsibility for maintaining adequate and accurate hard copy source documents of all observations and data generated during this study, including the hard copy or discs received from the Sponsor of the final data. Such documentation is subject to inspection by the Sponsor or its agents, the FDA and/or other regulatory agencies.

29.0 REFERENCES

Agostoni C, Trojan S, Bellù R, Riva E, Giovannini M. Neurodevelopmental quotient of healthy term infants at 4 months and feeding practice: the role of long-chain polyunsaturated fatty acids. Pediatr Res. 1995;38(2):262–266.

Ashkar S, Weber GF, Panoutsakopoulou V, Sanchirico ME, Jansson M, Zawaideh S et al. Eta-1 (osteopontin): an early component of type-1 (cell-mediated) immunity. Science. 2000; 287:860–864.

Auestad N, Montalto MB, Hall RT, et al. Visual acuity, erythrocyte fatty acid composition, and growth in term infants fed formulas with long chain polyunsaturated fatty acids for one year. Ross Pediatric Lipid Study. Pediatr Res. 1997;41(1):1–10.

Ballard O and Morrow, A. Human mik composition: Nutrient and bioactive factors. Pediatr Clin North Am. 2013;60(1):49-74.

Birch EE, Garfield S, Hoffman DR, Uauy R, Birch DG. A randomized controlled trial of early dietary supply of long-chain polyunsaturated fatty acids and mental development in term infants. Dev Med Child Neurol. 2000;42(3):174–181.

Bode L. Human milk oligosaccharides: every baby needs a sugar mama. Glycobiology 2012;22:1147–62.

Brenna JT, Varamini B, Jensen RG, Diersen-Schade DA, Boettcher JA, Arterburn LM. Docosahexaenoic and arachidonic acid concentrations in human breast milk worldwide. Am J Clin Nutr. 2007;85(6):1457-64.

Brodbeck U, Denton WL, Tanahashi N, Ebner KE. The isolation and identification of the B protein of lactose synthetase as alpha-lactalbumin. J Biol Chem. 1967; 242: 1391-1397.

Canfield LM, Clandinin MT, Davies DP, Fernandez MC, Jackson J, Hawkes J, Goldman WJ, Pramuk K, Reyes H, Sablan B, Sonobe T, Bo X. Multinational study of major breast milk carotenoids of healthy mothers. Eur J Nutr. 2003;42(3):133-41.

Capeding R, Gepanayao CP, Calimon N, Lebumfacil J, Davis AM, Stouffer N, Harris BJ. Lutein-fortified infant formula fed to healthy term infants: evaluation of growth effects and safety. Nutr J. 2010; 21; 9:22.

Cheng JB, Wang JQ, Bu DP, Liu GL, Zhang CG, Wei HY, et al. Factors affecting the lactoferrin concentration in bovine milk. J Dairy Sci. 2008;91(3):970–6.

Clandinin M, Chappell J, Leong S. Intrauterine fatty acid accretion rates in human brain: implication for fatty acid requirements. Early Hum Dev. 1980; 4:121–130.

Clandinin M, Chappell J, Leong S. Extrauterine fatty acid accretion rates in human brain: implication for fatty acid requirements. Early Hum Dev. 1980;4:131–138.

Coppa GV, Pierani P, Zampini L, Carloni I, Carlucci A, Gabrielli O. Oligosaccharides in human milk during different phases of lactation. Acta Paediatr. 1999; 88(430):S89-S94.

Davidson B, Meinzen-Derr JK, Wagner CL, Newburg DS, Morrow AL. Fucosylated oligosaccharides in human milk in relation to gestational age and stage of lactation. Adv Exp Med Biol. 2004; 554():427-30.

DHHS. (2000a, May 11, 2004). Growth chart training module. Accurately Weighing and Measuring Infants, Children and Adolescents: Technique. Health Resources and Services Agency, Maternal and Child Health Bureau. Retrieved February 3, 2017, from the World Wide Web: http://www.cdc.gov/nccdphp/dnpa/growthcharts/training/modules/modules.htm

Dupont C, Rivero M, Grillon C, Belaroussi N, Kalindjian A, Marin V. Eur J Clin Nutr. 2010 Jul;64(7):765-7.

Fleisler S, Anderson RE. Chemistry and metabolism of lipids in the vertebrate retina. Prog Lipid Res. 1983;22:79–131.

Innis SM1, Dyer R, Nelson CM. Evidence that palmitic acid is absorbed as sn-2 monacylglycerol from human milk by breast-fed infants. Lipids. 1994;29(8):541-5.

Jackson JG, Janszen DB, Lonnerdal B, Lien EL, Pramuk KP, Kuhlman CF. A multinational study of alpha-lactalbumin concentration in human milk. J Nutr Biochem. 2004; 15: 517-521.

Johnston WH, Ashley C, Yeiser M, Harris CL, Stolz SI, Wampler JL, Wittke A, and Cooper TA. Growth and tolerance of formula with lactoferrin in infants through one year of age: double-blind, randomized, controlled trial. BMC Pediatr. 2015; 15: 173.

Kennedy K1, Fewtrell MS, Morley R, Abbott R, Quinlan PT, Wells JC, Bindels JG, Lucas A. Double-blind, randomized trial of a synthetic triacylglycerol in formula-fed term infants: effect on stool biochemistry, stool characteristics, and bone mineralization. Am J Clin Nutr. 1999 Nov;70(5):920-7.

King J, Cummings G, Guo N, Trivedi L, Readmond B, Keane V, et al. A double-blind, placebo-controlled, pilot study of bovine lactoferrin supplementation in bottle-fed infants. J Pediatr Gastroentrol Nutr. 2007;44:245–51.

Krinsky NI, Johnson EJ. Carotenoid actions and their relation to health and disease. Mol Aspects Med. 2005;26(6):459-516.

Landrum JT, Bone RA. Lutein, zeaxanthin, and the macular pigment. Arch Biochem Biophys. 2001.1; 385(1):28-40.

Lien EL. The role of fatty acid composition and positional distribution in fat absorption in infants. J Pediatr. 1994;125:S62-8.

Lien EL, Davis AM, and Multicenter group. Growth and Safety of a reduced protein formula enriched with bovine alpha-lactalbumin in term infants. 2004; J Pediatr Gastroenterol Nutr. 170-176.

Litmanovitz I, Bar-Yoseph F, Lifshitz Y, Davidson K, Eliakim A, Regev RH, Nemet D. Reduced crying in term infants fed high beta-palmitate formula: a double-blind randomized clinical trial. BMC Pediatr. 2014 Jun 19;14:152.

Lohman T, Roche AF, Martorell R. *Anthropometric standardization reference manual.* Champaign, IL: Human Kinetics Books; 1988.

Lonnerdal B. Digestibility and absorption of protein in infants. In: Protein Metabolism During Infancy, ed. Raiha NCR. Vevey: Raven Press. 1994; 53-65.

Lonnerdal B. Infant formula and infant nutrition: bioactive proteins of human milk and implications for composition of infant formulas. Am J Clin Nutr. 2014;99(3):712S–7S.

Lönnerdal B, Kvistgaard AS, Peerson JM, Donovan SM, Peng YM. Growth, nutrition, and cytokine response of breast-fed infants and infants fed formula with added bovine osteopontin. J Pediatr Gastroenterol Nutr. 2016 Apr;62(4):650-7.

Makrides M, Neumann MA, Simmer K, Gibson RA. A critical appraisal of the role of dietary long-chain polyunsaturated fatty acids on neural indices of term infants: a randomized, controlled trial. Pediatrics. 2000;105(1 pt 1):32–38.

Manzoni P, Stolfi I, Messner H, Cattani S, Laforgia N, Romeo MG, Bollani L, Rinaldi M, Gallo E, Quercia M, Maule M, Mostert M, Decembrino L, Magaldi R, Mosca F, Vagnarelli F, Memo L, Betta PM, Stronati M, Farina D, Italian Task Force for the Study and Prevention of Neonatal Fungal Infections–the Italian Society of Neonatology. Bovine lactoferrin prevents invasive fungal infections in very low birth weight infants: a randomized controlled trial. Pediatrics. 2012 Jan; 129(1):116-23.

Moro G, Minoli I, Mosca M, Fanaro S, Jelinek J, Stahl B, Boehm G. Dose-related bifidogenic effects of galacto- and fructo-oligosaccharides in formula-fed term infants. J Pediatr Gastroenterol Nutr. 2002 Mar;34(3):291-5.

Nelson SE, Rogers RR, Ziegler EE, Fomon SJ: Gain in weight and length during early infancy. Early Human Development. 1989; 19 (4):223-239.

Neuringer M, Connor W, Lin D, Barstad L, Luck S. Biochemical and functional effects of prenatal and postnatal ω-3 fatty acid deficiency on retina and brain in rhesus monkeys. *Proc Natl Acad Sci.* 1989*;*83:285–294.

Ochoa TJ, Cleary TG. Effect of lactoferrin on enteric pathogens. Biochimie. 2009;91(1):30–34.

O'Connor DL, Hall R, Adamkin D, et al. Ross Preterm Lipid Study Growth and development in preterm infants fed long-chain polyunsaturated fatty acids: a prospective, randomized controlled trial. Pediatrics. 2001;108(2):359–371

Pabst HF, Spady DW, Pilarski LM, Carson MM, Beeler JA, Krezolek MP. Differential modulation of the immune response by breast- or formula-feeding of infants. Acta Paediatrica. 1997:1291–1297.

Roberfroid M. Prebiotics: the concept revisited. J Nutr. 2007;137(3 Suppl 2):830S–837S.

Scalabrin DM, Mitmesser SH, Welling GW, Harris CL, Marunycz JD, Walker DC, Bos NA, Tölkkö S, Salminen S, Vanderhoof JA. New prebiotic blend of polydextrose and galacto-oligosaccharides has bifidogenic effect in young infants. J Pediatr Gastroenterol Nutr. 2012; 54(3):343-52.

Schack L, Lange A, Kelsen J, Agnholt J, Christensen B, Petersen TE, et al. Considerable variation in the concentration of osteopontin in human milk, bovine milk, and infant formulas Journal of Dairy Science. 2009; 92:5378–5385.

Smilowitz JT, Lebrilla CB, Mills DA, German JB, Freeman SL. Breast milk oligosaccharides: structure-function relationships in the neonate. Ann Rev Nutr. 2014; 34: 143–169.

Trabulsi J, Capeding R, Lebumfacil J, Ramanujam K, Feng P, McSweeney S, Harris B, DeRusso P. Effect of an α-lactalbumin-enriched infant formula with lower protein on growth.
Eur J Clin Nutr. 2011; 65(2):167-74.

Yao M, Lien EL, Capeding MRZ, Fitzgerald M, Ramanujam K, Yuhas R, Northington R, Lebumfacil J, Wang L, DeRusso PA. Effects of term infant formulas containing high sn-2

palmitate with and without oligofructose on stool composition, stool characteristics, and bifidogenicity. J Pediatr Gastroenterol Nutr. 2014; 59(4): 440–448.

Ziegler E1, Vanderhoof JA, Petschow B, Mitmesser SH, Stolz SI, Harris CL, Berseth CL. Term infants fed formula supplemented with selected blends of prebiotic grow normally and have soft stools similar to those reported in breast-fed infants. J Pediatr Gastroenterol Nutr. 2007 Mar;44(3):359-64.

30.0 APPENDICES

30.1 Appendix A: Anthropometry Data Collection Procedures

All sites will be trained on standardized techniques for the measure of weight, length, and head circumference (Lohman, 1988).

Weight

Two people are involved with infant weight measures. One measurer will weigh the infant and read the weight as it is obtained. The other measurer will immediately note the measurement in the infant's chart.

The infant's clothing and diaper are removed. The infant should be positioned in the center of the scale tray. Infants should be weighed to the nearest 10 grams on a calibrated scale. Record the weight as soon as it is completed. Then the infant should be re-positioned and the weight measurement repeated and noted in writing. After the infant is removed from the scale tray, the weights should be compared and they should agree within 10 grams. If the difference between the weights exceeds the tolerance limit of 10 grams, the infant should be re-positioned and reweighed a third time. The two weights that are within 10 grams of one another should be recorded. If there are NOT two weights within 10 grams of one another, instrument error may be influencing the measure; the investigator should calibrate the scale, check the position of the scale and infant, etc. and then re-weigh the infant.

Summary:

- Remove infant's clothing and diaper
- Center the infant on the scale tray
- Weigh infant to nearest 10 grams
- Write the weight on the infant's chart
- Reposition and repeat weighing the infant
- Compare weights
- Weight should agree within 10 grams (tolerance of the measure)

Length

Length will be measured in the recumbent position with a calibrated lengthboard. The

lengthboard must have 1) a fixed headpiece and 2) a moveable footpiece, which is perpendicular to the surface of the table that the length board is on.
Length measurements for infants should be obtained while the infant is dressed in light underclothing or a diaper. The infant's shoes must be removed. Hair ornaments should be removed from the top of the head.

The infant should be placed on his/her back in the center of the length board so that the infant is lying straight and his/her shoulders and buttocks are flat against the measuring surface. The infant's eyes should be looking straight up. Both legs should be fully extended and the toes should be pointing upward with feet flat against the foot piece.

Two people must be involved with infant length measures. One measurer holds the infant’s head, with the infant looking vertically upward and the crown of the head in contact with the headpiece in the Frankfort Horizontal Plane). The head of the infant is firmly but gently held in position. The measurer gently cups the infant’s ears while holding the head in proper alignment. Make sure the infant's chin is not tucked in against his chest or stretched too far back.

While one measurer holds the infant’s head in the proper position, the second measurer aligns the infant’s trunk and legs, extends both legs, and brings the footpiece firmly against the heels. The measurer places one hand gently but firmly on the infant’s knees to maintain full extension of the legs. The infant’s toes are pointing upward with the soles of the feed perpendicular to the horizontal backpiece of the measuring device. It is imperative that both legs are fully extended for an accurate and reproducible length measurement.

The measurer at the feet should read aloud to the recorder the length measurement to the nearest 0.1 cm. The length should be recorded on the data form as soon as it is completed. Then the infant should be repositioned and the length measurement repeated and noted in writing. After the infant is removed from the lengthboard, the length measurements should be compared and they should agree within 0.5 cm.

If the difference between the two length measures exceeds 0.5 cm, the infant should be repositioned and remeasured a third time. The third measure of length should be within 0.5 cm

of either the first or second length measure; and the two measures within 0.5cm should be recorded. If there are NOT two measures of length within 0.5cm of one another, instrument error may be influencing the infantometer; the investigator should calibrate the infantometer, re-position the infant and re-measure the infant. The two measures that are within 0.5cm of one another should be recorded.

Summary:

- Use a calibrated lengthboard with a fixed headpiece and movable footpiece which is perpendicular to the surface of the table
- Measure infant without shoes and wearing light underclothing or diaper
- Measure length to 0.1 cm
- Record measurement on chart
- Reposition and remeasure infant
- Measurements should agree to 0.5 cm

Head Circumference

The goal of the head circumference measure is to locate the maximum circumference of the head. Head circumference or OFC [occipital frontal circumference] is measured over the most prominent part on the back of the head (occiput) and just above the eyebrows (supraorbital ridges), i.e. the largest circumference of the head. Any braids, barrettes, or other hair decorations that will interfere with the measurement should be removed. The infant may be held in the arms or on the lap of the parent/guardian if they prefer.

The tape is positioned across the frontal bones just above the eyebrows, above the ears, and around the biggest part of the back of the head (the occiput). The goal is to locate the maximum tissues. The measurement is read to the nearest 0.1 cm and recorded on the chart. The tape should be repositioned and the head circumference re-measured. The measures should agree within 0.2 cm. If the difference between the measures exceeds 0.2 cm, the infant should be repositioned and re-measured a third time. The two measures that are within 0.2cm of one another should be recorded.

Summary:

- Use a flexible, non-stretchable tape
- The goal is to locate the maximum circumference of the head
- Position the tape just above the eyebrows on the supraorbital ridge, above the ears, and around the biggest part on the back of the head (the occiput)
- Pull tape snugly to compress the hair
- Read the measurement to the nearest 0.1 cm
- Write measurement on the chart
- Reposition tape and re-measure the head circumference
- Measures should agree within 0.2 cm

Adapted from: Department of Health and Human Services, 2000

30.2 Appendix B: Formula Composition

	Building Block Nutritionals	Enfamil Premium 0-12 months
Nutrients/100 calories		
Protein, g	2.2	2.0
Fat, g	5.4	5.3
OPO Sn-2 oil	0.7	*
Carbohydrate, g	10.7	11.3
Dietary Fiber, mg	130.2	**
Galactoligosaccharide, mg	29.5	**
Polydextrose, mg	30	**
Fructoligosaccharide, mg	70.7	*
Linoleic Acid, mg	850	800
Vitamins:		
Vitamin A, IU	300	300
Vitamin D, IU	60	60
Vitamin E, IU	2	2
Vitamin K, mcg	9	9
Vitamin B1, mcg	80	80
Vitamin B2, mcg	140	140
Vitamin B3, mcg	60	60
Vitamin B6, mcg	0.3	0.3
Vitamin B12, mcg	1000	1000
Folic acid, mcg	16	16
Pantothenic Acid, mcg	600	500
Biotin, mcg	3	3
Vitamin C, mg	12	12
Minerals:		
Calcium, mg	78	78
Phosphorus, mg	43	43
Magnesium, mg	8	8
Iron, mg	1.8	1.8
Zinc, mg	1.0	1.0
Manganese, mcg	15	15
Copper, mg	75	75
Iodine, mcg	10	15
Sodium, mg	27	27
Potassium, mg	108	108
Chloride, mg	63	63
Selenium, mcg	2.8	2.8

	Building Block Nutritionals	Enfamil Premium 0-12 months
Choline, mg	24	24
Inositol, mg	6	6
B-carotene	19	*
Lutein	18	*
Nucleotides, mg	3.9	**
L-Carnitine, mg	1.4	**
Taurine, mg	5.8	**
Osteopontin, mg	18	*
Lactoferrin, mg	7.8	*
Docosahexaenoic acid (DHA) mg	9.7	**
Arachidonic acid (ARA) mg	19.4	**
Data from Enfamil™ Web Sites as of Jan 25, 2017<br>*Not Added to Formula<br>**Nutritional Information not available		

Label for Enfamil Premium 0-12 months

30.3 Appendix C: Infant Formula Ingredients

Formula A

Nonfat Milk, Lactose, Vegetable Oil (Palm Olein, Coconut, Soy and High Oleic Sunflower Oils), Whey protein concentrate, Polydextrose*, Galactoligosaccharides* and lessthan 1% Mortierella Alpina Oil** Crypthecodinium Cohnii Oil** Calcium Carbonate, Potassium citrate, Ferrous sulfate, Potassium chloride, Magnesium oxide, Sodium chloride, Zinc sulfate, Cupric Sulfate, Manganese sulfate, Sodium Selenite, Soy lecithin, Choline chloride, Ascorbic acid, Niacinamide, Calcium Pantothenate, Vitmin A palmitate, Vitamin B12, Vitamin D3, Riboflavin, Thiamin Hydrochloride, Vitamin B6 Hydrochloride, Folic acid, Vitamin K1, Biotin, Inositol, Vitamin E acetate, Nucleotides (Cytidine 5'-monophosphate, Disodium Uridine 5'-monophosphate, Adenosine 5'-monophosphate,Disodium Guanosine 5'-monophosphate) Taurine and L-Carnitine.

*A Type of Prebiotic
**A Source of Arachidonic acid (ARA)
**A Source of Docosahexaenoic acid (DHA)

Formula B

Nonfat Milk, Lactose, Vegetable Oil (OPO Sn-2 Oil, Coconut, Soy and High Oleic Sunflower Oils), Demineralized Whey, Whey protein concentrate, Alpha lactlbumin, and lessthan 1% Polydextrose*, Galactoligosaccharides*, Fructooligosaccharides*, Mortierella Alpina Oil Powder** Crypthecodinium Cohnii Oil Powder** Calcium Carbonate, Dicalcium Phosphate, Potassium citrate, Ferrous sulfate, Potassium chloride, Potassium Iodide, Magnesium chloride, DiMagnesium phosphate, Sodium citrate, Zinc sulfate, Copper Sulfate, Manganese sulfate, Sodium Selenite, Soy lecithin, L-Choline bitartrate, Ascorbic acid, Niacinamide, Calcium Pantothenate, Vitmin A palmitate, Vitamin B12, Vitamin D3, Riboflavin, Thiamin Hydrochloride, Vitamin B6 Hydrochloride, Folic acid, Vitamin K1, Biotin, Inositol, Vitamin E acetate, Nucleotides (Cytidine 5'-monophosphate, Disodium Uridine 5'-monophosphate, Adenosine 5'-monophosphate,Disodium Guanosine 5'-monophosphate), Beta carnotene, Lutein, Osteopontin, Lactoferrin, Taurine and L-Carnitine

*A Type of Prebiotic
**A Source of Arachidonic acid (ARA)
**A Source of Docosahexaenoic acid (DHA)

30.4 Appendix D: Sample Investigational Product Labels

30.5 Appendix E: Investigational Product Accountability

Completing the Investigational Product (IP) Account Forms (Site Inventory)

The initial IP shipment is initiated by BBN upon receipt of the required site regulatory documentation. IP is supplied by BBN and shipped by a qualified distributor to each clinical study site. IP is shipped via courier on a Monday, Tuesday, or Wednesday. Shipments on Thursday should only occur with prior clearance from BBN. Shipments for Saturday delivery will not be sent.

The responsible site designee is to document each shipment of IP received and each case/can of IP dispensed in the supplied Site IP Accountability Log (Appendix G). In order to fully account for each case/can of IP received and used throughout the study, multiple pages of the accountability logs may be needed. Pagination is documented in the designated page number spaces located at the bottom right-hand corner of the form.

Each shipment received is documented on the Site IP Accountability Log at the time of receipt. All cases of IP must be opened to verify that all IP cans are in tact and are numbered with the same number as on the case. Any cases/cans damaged in shipment will not be available to dispense and will be reported to Paidion upon receipt. Any revisions or errors on the Site IP Accountability Log must be crossed out with a single line, initialed and dated. The following fields are collected on the Site IP Accountability Log for each shipment received:

- Date shipment received
- Case number(s) and can number(s)
- Number of cases received
- Number of cases dispensed
- Patient ID
- Number of cases available
- Number of cases/cans unused but not available
- Monitor verification

When all lines on the Site IP Accountability Log are completed, record the total number of cases received and dispensed at the bottom of the form. Transfer the total into a new log sheet to continue tracking accountability. The site designee is to sign and date at the bottom of each page to verify the accuracy and completeness of the information.

Patient level accountabilities are recorded on the Patient IP Accountability Log (Appendix H). The following fields are collected on the Patient IP Accountability Log for each case dispensed:

- Investigator Name

- Site number
- Patient ID
- Patient initials, if applicable
- Date of dispensing
- Case/can number(s)
- Number of dispensed cases
- Number of used cases/cans returned
- Data of return
- Principal Investigator, Study Nurse, or designee initials and date
- Monitor verification

Completed Accountability Logs must be filed in the appropriate section of the Investigator Site File or designated location.

All used, partially used, or compromised IP must be retained in the original container until reconciliation is completed by the Sponsor or an authorized representative and shipment instructions and/or destruction permission is granted. Please store all used cases/cans with IP label in order to facilitate accountability.

30.6 Appendix F: Standardized definitions for common Adverse Events that may or may not be related to formula tolerance (stooling, spit-up, crying, skin issues)

All AEs must be assigned an intensity score and causality (related or not related to the investigational product; see **section 20.1** for details).

ADVERSE EVENT	DEFINITION
**Stooling Issues**	
Difficulty having bowel movement	Crying, fussing, or turning red when having a bowel movement
Hard stools	Healthcare professional diagnosis; pellet or hard rock-like stools
Constipation	Less than 3 bowel movements in 7 days
Acute diarrhea	Runny or watery stools for less than 2 weeks
Chronic diarrhea	Runny or watery stools for more than 2 weeks or ≥3 separate episodes of acute diarrhea in 2 weeks
**Spit-up, Vomiting, GERD issues**	
Regurgitation	Milk comes up into mouth but never out of mouth AND infant DOES NOT arch his/her back as if in pain, stop drinking even if hungry, or cry, wheeze, or cough related to feedings
Infantile spit up	Milk comes out of the mouth after feeding (typically non-forceful), and the amount that comes out is less than half of the feeding volume; non-projectile
Vomiting	Milk comes out of the mouth after feeding (typically forceful), and the amount that comes out is more than half of the feeding volume
Gastroesophageal Reflux Disease (GERD)	Baby arch his/her back as if in pain, stop drinking even if hungry, or cry, wheeze or cough related to feedings (with or without milk coming up into mouth)
**Crying issues**	
Crying/Neonatal abnormal crying	Infant cries for 3 or more hours per day
Infantile colic/Infant colic	Infant cries inconsolably for 3 or more hours per day, at least 3 days per week, AND for at least 3 weeks
**Skin issues**	
Diaper rash	Contact/irritant dermatitis in the diaper area, with erythema and/or skin breakdown on the exposed convex skin surface (rate as Mild, Moderate, Severe using definitions outlined below)<br>**Mild**: Baby has an area of pinkness in the diaper area

ADVERSE EVENT	DEFINITION
	**Moderate**: Baby has definite pinkness in a large area with some small areas of definite redness<br>**Severe**: Baby has intense redness over a large area of the perianal region, or any area of redness with skin breakdown in the diaper area
Atopic dermatitis/Eczema	Any new skin lesions, not in the diaper area. Score for any erythema, edema/papulation, and/or excoriation on scale from 0-3 each (9 points max).<br>For each item, 0=none, 1= mild, 2=moderate, 3= severe.<br>**Mild:** 1 or 2 points<br>**Moderate:** 3 to 5 points<br>**Severe:** 6 to 9 points

30.7 Appendix G: Site Investigational Product Accountability Log

SITE INVESTIGATIONAL PRODUCT ACCOUNTABILITY LOG

INVESTIGATOR	PROTOCOL NUMBER	SITE NUMBER
	BBN-IF-001-01	

					IP Receipt	IP Dispensing		Inventory		Verification	
Date	Case #	# of Cans	Can #'s	Do Can and Case #s match? (Yes/No)	# of Cases Received:	# of Cases Dispensed:	Subject ID	Number of Cases Available:	# of Cases Unused but NOT AVAILABLE*	Site Designee: (Initial/Date)	Monitor Verification: (Initial/Date)
					TOTALS:						

Printed Name of Site Personnel | Signature of Site Personnel | Date

1. Complete one line for each entry. Draw a single line through any fields which are not applicable (e.g. Complete "IMP Receipt" section, draw line through "IMP Preparation section").
2. At the time of IP receipt, complete Date, Case #, verify # of cans in case, Can #'s, verify all cans numbers are identical and match case #, and the field listed under "IP Receipt".
3. At the time of dispensing, complete Date, Case #, and the fields listed under "IP Dispensing"
4. The site designee should initial and date each entry, and sign and date the form after all lines are completed. The form should be maintained in the Investigator Site File; a copy will be collected by the CRA.

30.8 Appendix H: Subject Investigational Product Accountability Log

SUBJECT IP ACCOUNTABILITY LOG

INVESTIGATOR	PROTOCOL NUMBER	SITE NUMBER	SUBJECT ID
	BBN-IF-001		

	IP Dispensing		IP Return			Verification	
Date	Case/Can No.	No. of Cases	Case/Can No.	No. of Cans	Comment	Site Designee: (Initial/Date)	Monitor Verification: (Initial/Date)

Printed Name of Site Personnel

Signature of Site Personnel

Date

1. Complete one line for each entry. Draw a single line through any fields which are not applicable (e.g. Complete “IP Dispensing” section, draw line through “IP Return section”).
2. At the time of dispensing, complete Date and all fields under “IP Dispensing”.
3. At the time of return, complete Date and all fields under “IP Return”.
4. The site designee should initial and date each entry, and sign and date the form after all lines are completed. The form should be maintained in the Investigator; a copy will be collected by the CRA.

30.9 Appendix I: BBN-IF-001 Study Infant Formula Preparation Instructions

This study infant formula preparation instruction is appropriate for both Study Infant Formula A and Infant Formula B.

Proper hygiene, study formula preparation, dilution, use and storage are important to the well-being of your baby.

Ask your baby's doctor about the need to use cooled, boiled water when preparing the study formula and whether you need to boil utensils, bottles, nipples and rings in water before each use. If you are concerned about lead or other harmful substances in your water, talk to your healthcare professional before making study formula with tap water.

HOW TO MIX BBN-IF-001 STUDY INFANT FORMULAS:
For proper mixing, follow these steps:

1. Wash your hands thoroughly with soap and warm water.
2. Following the measurement guidelines below, add the appropriate amount of water and scoops of study Formula A or Formula B powder to the bottle:

Measure water	Add scoop(s) of unpacked level powder using enclosed scoop	Finished bottle (approx.)
2 fl oz	1 scoop	2 fl oz
4 fl oz	2 scoops	4 fl oz
6 fl oz	3 scoops	6 fl oz
8 fl oz	4 scoops	8 fl oz

3. Put the cap on the bottle and shake
4. Feed prepared study formula immediately (within 2 hrs of preparation) or cover and store in the refrigerator for no longer than 24 hours.
   Warning: Do Not Use a Microwave to Warm Formula. Serious Burns May Result

Growth and Safety Study of an Infant Formula for Healthy Term Infants

Date of Protocol: 07 January 2021
Version 5.2

Investigational Product:	Infant Formula
Sponsor:	Building Block Nutritionals, LLC
Sponsor Contact:	James McGrath, Jr<br>Executive Vice President<br>200 Garrett St. #S<br>Charlottesville, VA 22902<br>Phone: 628-400-7617<br>Email: jmcgrath@bbnutritionals.com
Medical Monitor:	Kumar Ilangovan, MD<br>Paidion Research, Inc.<br>240 Leigh Farm Road<br>Suite 175, Durham, NC 27707<br>Phone: 919-885-1911<br>Email: kumar.ilangovan@paidion.com

Signature of Approval of Protocol Version 5.2 dated 07 January 2021 and Acknowledgement of Responsibilities

SPONSOR'S APPROVAL AND AUTHORIZATION

Jim McGrath

Sponsor Name (Printed)

EVP

1/7/2021

Sponsor Role/Position

Date

INVESTIGATOR'S AGREEMENT (*Acknowledgment of Responsibilities*)

This protocol is the property of Building Block Nutritionals, LLC. I understand that the information within it is confidential and is provided to me for review by myself, my staff, and applicable ethics committees. I understand that the protocol must be kept in a confidential manner and must be returned to the Sponsor, Building Block Nutritionals, LLC, or destroyed per Building Block Nutritionals, LLC instructions, upon request. No part of this protocol may be reproduced in any form without written authorization from Building Block Nutritionals, LLC. By accepting this protocol, I agree that the information contained herein will not be disclosed to a third party without written authorization from Building Block Nutritionals, LLC.

I have read and understood the protocol and agree that it contains all of the necessary information to carry out the study.

I agree to conduct this trial in accordance with all stipulations of the protocol and in accordance with the following: Good Clinical Practice, the ethical principles that have their origin in the Declaration of Helsinki; Title 21 of the Code of Federal Regulations, Parts 50 (Protection of Human Subjects), and 56 (Institutional Review Boards), and 312 (Investigational New Drug Application); and International Council for Harmonisation E6 (Guideline for Good Clinical Practice).

I agree that I will not modify this protocol without obtaining the prior approval of the Sponsor and of the institutional review board or independent ethics committee, except when necessary to protect the safety, rights, or welfare of subjects.

Principal Investigator Name (Printed)

Signature

Date

Site Address or Site Number

1 PROTOCOL SYNOPSIS

**Sponsor:** Building Block Nutritionals, LLC
**Title of Study:** Growth and Safety Study of an Infant Formula for Healthy Term Infants
**Protocol Number:** BBN-IF-001
**Rationale:**<br>A goal of infant formula development is to mimic human milk (HM) both in nutrient composition as well as physiologic outcomes. We have developed two infant formulas for term infants that more closely resembles the composition of human milk. The purpose of this study is to demonstrate that this formulation meets nutritional requirements and supports age appropriate growth of healthy term infants.
**Objectives:**<br>The primary efficacy objective is to compare the growth of infants randomized to a commercially available term infant formula (Brand Formula) versus growth of infants randomized to two experimental infant formulas for term infants (BBN-001 w/ OPN Formula and BBN-102 w/o OPN).<br><br>The secondary efficacy objective is to compare formula intake volume between formula groups.<br><br>The exploratory objective is to compare serum markers of inflammation between formula groups.<br><br>The primary safety objective is to compare the frequency of adverse events (AEs) between the formula groups.<br><br>The secondary safety objective is to compare the gastrointestinal tolerance (stool composition, bowel movements, stool consistency, gas, fussiness, and ICQ scales) between formula groups.<br><br>*Analyses between cohorts (Brand Formula, BBN-001* w/ OPN Formula*, and BBN-102 w/o OPN) will occur separately depending on finalization of Generally Recognized as Safe (GRAS) approval of specific ingredients in BBN-001 w/ OPN Formula.*
**Study Design:**<br>This study is a randomized, controlled, double-blind study of healthy term formula-fed (FF) infants.<br>The first phase of the study, infants will receive either a new infant formula formulated for healthy term infants (BBN-001 w/ OPN Formula) or a commercially available infant formula for healthy term infants (Brand Formula) in a 1:1 ratio.<br>After completion of the first phase, in a second phase of the study, infants will be randomized to receive either Brand Formula or BBN-102 w/o OPN in a 1:8 ratio.<br>In both study arms, infants will consume the study formula for a total of 16 weeks. Throughout the study, infant growth and tolerance to the formulas will be assessed.

Criteria for Inclusion:

Infants will be eligible to participate if they meet all of the following conditions.

At birth the infant must be:

1. Healthy, term (early term/no less than 37 weeks, 0 days through late term/no greater than 41 weeks, 6 days), singleton infant
2. Have a birth weight of □ 2500 grams

At the time of the baseline/enrollment visit, infants must be:

3. Designated as healthy by a physician
4. :S 14 days post-natal age (Date of Birth = Day 0)
5. Weight for age □ 5th and :S 95th percentile for age according to sex-specific World Health Organization (WHO) growth charts for infants and children ages 0 to 2 years of age
6. Length for age □ 5th and :S 95th percentile for age according to sex-specific charts World Health Organization (WHO) growth charts for infants and children ages 0 to 2 years of age
7. Head circumference for age □ 5th and :S 95th percentile for age according to sex-specific World Health Organization (WHO) growth charts for infants and children ages 0 to 2 years of age
8. Weight for length for age □ 5th and :S 95th percentile for age according to sex-specific World Health Organization (WHO) growth charts for infants and children ages 0 to 2 years of age
9. Exclusively consuming and tolerating a cow's milk infant formula at time of enrollment; only infants whose parent(s) or legal guardian(s) have decided to feed infant formula as the sole source of nutrition will be approached for potential study enrollment
10. Have parent(s) or legal guardian(s) who agree to feed the study formula to the study subject as his/her sole source of nutrition for the duration of the study
11. Have parent(s) or legal guardian(s) who have read and voluntarily signed an Informed Consent form approved by the Institutional Review Board prior to any participation in the study

Criteria for Exclusion:

Infants will be ineligible if they have any of the following conditions that are judged by a physician to interfere with the infant's normal growth, development, and/or tolerance to an infant formula:

1. Show evidence of anatomic and physiologic defects of the respiratory tract, or other congenital defects (as determined by the clinician)
2. Show evidence of chronic hepatic, gastrointestinal, renal, cardiac, pulmonary, or neurological diseases
3. Have a maternal history with known adverse effects on the fetus and/or the newborn infant, such as diabetes (gestational diabetes is acceptable if infant's birth weight is < 4300 g), active tuberculosis, perinatal infection, or substance abuse
4. Have a family history of cow's milk protein intolerance/allergy
5. Are an infant from a multiple birth (twin, triplet, etc.)

Investigational Product, Dose, and Mode of Administration:

Infants will consume ad libitum per day one of the following formulas:

Brand Formula: A commercially available infant formula for term infants; 100 kcal/5 fl. oz, 2 g protein/100 kcal (Enfamil Infant 0-12 months by Mead Johnson Nutrition, LLC)

BBN-001 w/ OPN Formula: An infant formula for term infants containing alpha- lactalbumin enriched whey, OPO Sn-2 oil, osteopontin, lactoferrin, pre-biotics (PD, FOS, GOS), lutein, microencapsulated DHA/ARA; 100 kcal/5 fl. oz, 2.2 g protein/100kcal (Manufactured by Building Block Nutritionals, LLC)
BBN-102 w/o OPN : An infant formula for term infants containing alpha-lactalbumin enriched whey, OPO Sn-2 oil, lactoferrin, pre-biotics (FOS, GOS), lutein, DHA/ARA; 100 kcal/5 fl. oz, 2.5 g protein/100 kcal (Manufactured by Building Block Nutritionals, LLC)

Primary Efficacy and Safety Evaluations:
Weight gain velocity (g/d)
Adverse Events will be collected throughout the 16-week study

Statistical Methods:
For phase one of the study, sample size estimation is based on a test for non-inferiority between Brand Formula and BBN-001 w/ OPN Formula. The criterion for non-inferiority in weight gain is that the difference in the two formula-fed groups' (Brand Formula and B) mean weight gain velocity (g/d) is significantly less than 3 g/d. Assuming a standard deviation in weight gain of 5.6 g/d (Nelson et al., 1989) and 80% power, 90 subjects per group (180 subjects total) will be sufficient to demonstrate non-inferiority (one sided alpha = 0.025). Assuming a 25% attrition rate, a total 256 subjects will be enrolled in the Brand Formula and BBN-001 w/ OPN Formula groups.

For phase two of the study, sample size estimation is based on the primary endpoint, weight gain velocity (g/d) over a 16-week study period. Weight gain velocity will be compared between Brand Formula and BBN-102 w/o OPN using a non-inferiority margin of 3 g/d. A blinded interim analysis of 81 subjects who completed 16 weeks of the study was performed in phase one of the study to estimate the standard deviation of weight gain. Based on this analysis, it is assumed that the standard deviation in weight gain velocity is 6.0 g/d.
Assuming this standard deviation in weight gain of 6.0 g/d and approximately 90% power, approximately 168 subjects (104 in the Brand Formula group and 64 in the BBN-102 w/o OPN group) will be sufficient to demonstrate non-inferiority (one-sided alpha=0.025). Assuming a 25% attrition rate, a total of approximately 96 subjects will be enrolled in phase two.

The plan is to use the 129 subjects randomized to Brand Formula in the first phase of the study, assuming a 25% attrition rate and 96 subjects meet the Per Protocol population criteria. This data has and will remain blinded through the enrollment of the study. In phase two of the study, infants will be randomized to Brand Formula or BBN-102 w/o OPN in a 1:8 ratio. Assuming a 25% attrition rate and to obtain approximately 90% power, approximately 10 subjects randomized to Brand Formula and 86 subjects randomized to BBN-102 w/o OPN will be enrolled in phase two of the study. With the addition of the 129 subjects from phase one of the study, there will be approximately a total of 139 subjects enrolled in the Brand Formula group and 86 subjects enrolled in the BBN-102 w/o OPN group. Infants will be stratified by sex to achieve balance of males and females within each formula group.

TABLE OF CONTENTS

1 PROTOCOL SYNOPSIS 3
TABLE OF CONTENTS 6
LIST OF APPENDICES 8
LIST OF ABBREVIATIONS 9
2 STUDY FLOWCHART 11
3 ETHICS 12
3.1 Institutional Review Board 12
3.2 Ethical Conduct of Study 12
3.3 Informed Consent 12
4 BACKGROUND INFORMATION AND RATIONALE 12
4.1 Introduction 12
4.2 Alpha-lactalbumin 13
4.3 Lactoferrin 13
4.4 Osteopontin 14
4.5 OPO Sn-2 oil 14
4.6 Pre-biotics 15
4.7 Lutein 16
4.8 DHA and ARA 16
5 OBJECTIVES 16
6 STUDY DESIGN 17
6.1 Approximate Duration of Subject Participation 17
6.2 Approximate Number of Subjects 17
7 SELECTION OF SUBJECTS 17
7.1 Inclusion Criteria 17
7.2 Exclusion Criteria 18
8 PRIOR AND CONCOMITANT MEDICATION AND TREATMENT 19
9 PROCEDURES 20
9.1 Screening 20
9.2 Study Visit Procedures 20
10 INVESTIGATIONAL PRODUCT AND ADMINISTRATION 23
10.1 Study Formulas 23
10.2 Type and Amount 23
10.3 Administration 23
10.4 Formula Storage by Investigator 23
10.5 Formula Storage by Parent/Guardian 23

10.6 Subject Compliance 24
11 SAFETY 24
12 EFFICACY 24
12.1 Primary Efficacy Endpoint 24
12.2 Secondary Efficacy Endpoints 24
12.3 Exploratory Endpoint 24
13 LABORATORY DETERMINATIONS 24
13.1 Biological samples 25
14 STATISTICS 25
14.1 Statistical Methods 25
14.1.1 Primary Efficacy Endpoint 25
14.1.2 Safety Endpoints 25
14.1.3 General Methods 25
14.2 Statistical Power and Sample Size Considerations 26
14.3 Interim Analyses and Data Monitoring 27
15 SUBJECT IDENTIFICATION 27
16 INVESTIGATIONAL PRODUCT ACCOUNTABILITY 27
16.1 Formula Inventory 27
16.2 Formula Disposition 28
17 RANDOMIZATION 28
18 ADVERSE EVENTS 28
18.1 Definitions 28
19 SERIOUS ADVERSE EVENTS 29
20 ADVERSE EVENT AND SERIOUS ADVERSE EVENT RECORDING AND REPORTING 29
21 SUBJECT DISCONTINUATION OR WITHDRAWAL 30
22 INFORMED CONSENT 30
23 PROTOCOL AMENDMENTS 31
24 QUALITY CONTROL AND ASSURANCE 31
25 DIRECT ACCESS, DATA HANDLING, AND RECORD-KEEPING 31
26 RECORDS RETENTION 32
REFERENCES 33

LIST OF APPENDICES

Appendix A – Anthropometry Data Collection Procedures

Appendix B – Formula Composition

Appendix C – Formula Ingredients

Appendix D – Sample Formula Labels

Appendix E – Investigational Product Accountability

Appendix F – Standardized Definitions for Common Adverse Events

Appendix G – Formula Preparation Instructions

Appendix H – Version History and Summary of Changes

LIST OF ABBREVIATIONS

AE	Adverse event
ARA	Arachidonic acid
BBN	Building Block Nutritionals, LLC
bLF	bovine lactoferrin
CFR	Code of Federal Regulations
CI	confidence interval
cm	centimeter
CRF	Case report form
CRO	Clinical research organization
CSA	Clinical study agreement
CSR	Clinical study report
DHA	Docosahexaenoic acid
eCRF	Electronic case report form
FDA	Food and Drug Administration
FF	formula-fed
fl oz	fluid ounce
Formula A	Experimental Formula A (Building Block Nutritionals, LLC)
Formula B	Commercially Available Milk Infant Formula (Enfamil Infant 0-12 months)
Formula C	Experimental Formula C (Building Block Nutritionals, LLC)
FOS	Fructo-oligosaccharide
g	gram
GCP	Good Clinical Practice
GERD	Gastroesophageal Reflux Disease
GI	Gastrointestinal
GOS	Galacto-oligosaccharide
GRAS	Generally recognized as safe
HM	Human milk
HMO	Human milk oligosaccharide
ICF	Informed Consent Form
ICQ	Infant Characteristics Questionnaire
IEC	Independent ethics committee
IP	Investigational product
IRB	Institutional Review Board
ITT	Intent to treat
kcal	kilocalorie
kg	kilogram
L	liter
LCPFUA	long chain polyunsaturated fatty acid
LOS	lactulose
mcg	microgram
MedDRA	Medical Dictionary for Drug Regulatory Affairs
mg	milligram

OFC	occipital frontal circumference
OPN	Osteopontin
oz	ounce
PP	Per protocol
PWD	Powder
RTSM	Randomization and Trial Supply Management
SAE	Serious adverse event
SDV	Source document verification

2 STUDY FLOWCHART

Shaded columns indicate visit occurs at study site

**Study Visit**	**1**	**2**	**3**	**4**	**5**	**6**	**7**	**8**	**9**
**Study Day**	**0**	**15** ±3 days	**30** ±3 days	**45** ±3 days	**60** ±3 days	**75** ±3 days	**90** ±3 days	**105** ±3 days	**120** ±3 days
Informed consent	X								
Inclusion/Exclusion criteria	X								
Demography	X								
Randomization	X								
Infant feeding history	X								
Physical exam	X								
Medical history	X								
Maternal smoking history	X								
Anthropometry[1]	X	X	X		X		X		X
Stool Characteristics and Tolerance Questionnaire	X	X	X	X	X	X	X	X	X
Infant Characteristics Questionnaire (ICQ)	X	X	X		X		X		X
3-day Formula/Diet Record[2]	X	X	X		X		X		X
Blood Collection[3]									X
Dispense stool collection kit[4]							X		
Stool collection[4]									X
Telephone contact[5]	X			X		X		X	
Concomitant medications	X	X	X	X	X	X	X	X	X
Adverse events	X	X	X	X	X	X	X	X	X
Dispense study formula	X	X	X		X		X		
Collect unused study formula									X

[1]Anthropometry includes assessment of weight, length, and head circumference (see Appendix A for procedure details).

[2]Initial 3-day Formula/Diet Record will be recorded for 3 days following the first study visit. Remainder of 3-day Formula/Diet Records will be recorded for 3 days prior to each subsequent scheduled study visit.

[3]Blood collection to assess markers of inflammation (tumor necrosis factor-alpha, interleukin 2, 4, 5, 6, 8, 10, 12, 13, & 17; interleukin 2 receptor, interleukin 1 beta, and interferon gamma).

[4]Stool collection to assess stool composition. Stool collection supplies will be distributed to parents at Visit 7 for collection of all stools in the 3 days just before Visit 9. The stool collection kit is returned at Visit 9.

[5]Initial telephone contact will occur 3 days after enrollment to check compliance with study feeding and inquire about subject well-being. Remainder of telephone contacts will occur mid-way between clinic visits. Telephone contact notes will be recorded in the study subject's medical record. In the event a 3-day telephone contact falls on a holiday or weekend, the contact will be made on the next available business day.

3 ETHICS

3.1 Institutional Review Board

Clinical documentation for this study, including the clinical protocol, protocol amendments, and informed consent forms will be reviewed and approved by the IRB of each study site.

3.2 Ethical Conduct of Study

The study will be performed in accordance with the ethical principles that have their origin in the Declaration of Helsinki. The trial will be conducted in compliance with protocol, Good Clinical Practice, and other applicable regulatory requirements.

3.3 Informed Consent

Written informed consent will be obtained from the parent(s) or guardian(s) of each infant using the IRB-approved informed consent form before any study formulas are fed to the study subject or data are collected.

4 BACKGROUND INFORMATION AND RATIONALE

4.1 Introduction

Human milk (HM) is universally considered the gold standard for infant feeding. HM is a dynamic, multi-faceted fluid containing nutrients and bioactive factors needed for infant health and development. If an infant cannot be breastfed, the American Academy of Pediatrics recommends infant formula as the next best feeding alternative. A goal of infant formula development is to mimic HM in both nutrient composition as well as physiologic outcomes. For years, infant formulas have not been able to provide many of the bioactive factors found in HM.

The proteins of human milk are divided into the whey and casein fractions, with each comprised of specific proteins and bioactive peptides that confer nutritional and functional benefits (Ballard and Morrow, 2013). The most abundant proteins are casein, alpha-lactalbumin, lactoferrin, secretory immunoglobulin IgA, lysozyme, serum albumin, and osteopontin (Ballard and Morrow, 2013; Schack et al. 2009; Lonnerdal et al. 2014).

With a goal of providing infants who receive infant formula a nutritional product closer in composition to HM, improvements in infant formula composition are warranted. **This study is designed to evaluate the ability of BBN-001 w/ osteopontin (OPN) and BBN-102 w/o OPN to support age appropriate growth in healthy term infants.**

4.2 Alpha-lactalbumin

Alpha-lactalbumin is the predominant whey protein found in HM with a concentration of 2 to 3 g/L (Jackson et al., 2004); however, it is a relatively minor component of bovine milk whey. Alpha-lactalbumin has both a biochemical role and a nutritional role for the mother-infant dyad. During lactation, the mammary gland produces alpha-lactalbumin and galactosyltransferase. These two proteins form the enzyme complex lactose synthase, which catalyzes the synthesis of lactose from glucose and galactose (Brodbeck et al., 1967). The nutritional value of alpha-lactalbumin lies in its high proportion of essential amino acids, specifically tryptophan, cysteine, and lysine (Lonnerdal, 1994). Standard term infant formulas containing whey typically provide some (~1.2 g/L) bovine alpha-lactalbumin (Lien et al., 2004). Term infant formulas enriched with the whey protein alpha-lactalbumin have been developed and are currently marketed today.

Alpha-lactalbumin-rich formula retains a 60:40 whey to casein ratio and has an amino acid profile closer to the amino acid profile of HM. The higher biological value of alpha-whey protein also allows for a reduction of total protein closer to that of HM (14 g protein/L alpha-lactalbumin rich formula versus 16 g protein/L in standard term infant formulas). Lien et al. (2003) were the first to conduct a growth and safety study comparing the alpha-lactalbumin-rich formula to standard term infant formula. This study found that all parameters of growth for both groups were within age appropriate reference ranges, growth velocity was comparable between groups, and the alpha-lactalbumin-rich formula was well tolerated. Formula with alpha-lactalbumin enriched way have been marketed for over a decade and several other studies have documented the safety and suitability of infant formulas containing this whey (Trabulsi et al., 2011, DuPont et al., 2010). Alpha-lactalbumin-enriched whey protein concentrates used in this study are allowed to be used in infant formula in European Union (EU—no legal definition) and China (GB11674- 2010), are acceptable under Codex standard (289-1995) and approved as GRAS by the US FDA.

4.3 Lactoferrin

Lactoferrin is an iron-binding protein that is found in human milk. It plays a role in iron homeostasis, gastrointestinal defense against microbial infection, and has anti-inflammatory and immune modulatory effects (Lonnerdal 2014). It is found in the highest concentrations in colostrum but persists throughout the entire first year. Cow's milk, which is used to make standard infant formulas, contains small amounts of bovine lactoferrin (bLF; 30– 485 mg/L), but for the most part, unless added, does not contain appreciable amounts of bLF (Cheng et al., 2008). Human and bovine lactoferrin share 70% sequence homology and have been shown to have similar biologic effects (Ochoa et al., 2008). The safety and efficacy of infant formula supplemented with 850 mg/L bLF (King et al., 2007) was evaluated in a small (n = 52) 12-month placebo-controlled trial of healthy term infants. This study found that infants receiving formula supplemented with lactoferrin tolerated the formula, had significantly lower respiratory illness and higher blood hematocrit concentrations compared to infants fed standard formula (King et al., 2007). More recently a large-scale growth and safety study of infant formula supplemented with bLF was conducted. A total of 480 infants were enrolled and randomized to receive one of three formulas: 1) standard cow milk formula (control), 2)

an investigational formula with bLf at 600 mg/L, or 3) an investigational formula with 1000 mg lactoferrin/L, for the first year of life.
The investigational formulas also contained a pre-biotic blend of blend of polydextrose (PDX) and galactooligosaccharides (GOS). This study found that all formulas were well tolerated and there were no group differences in growth rate (g/d) from 14-365 days of age among the study groups. Infants receiving the experimental formulas had softer stools from day 30 to day 180 (Johnston et al., 2015). A study of preterm infant formula supplemented with lactoferrin also reported the formula was well tolerated, supported age appropriate growth, and resulted in decreased fungal infections (Manzoni et al., 2012). Taken together, these studies demonstrate the safety and efficacy of lactoferrin supplemented infant formula.

4.4 Osteopontin

Osteopontin (OPN) is a bioactive protein found in human and bovine milk; it is thought to play a role in immunity, wound healing, and bone remodeling (Lonnerdal, 2014). The concentration of OPN in HM averages 138mg/L (Schack et al., 2009) and cow's milk typically contains 1/10 the concentrations. Standard infant formulas contain 5.3 – 13.0mg/L bovine osteopontin (Schack et al., 2009). OPN is a key cytokine in the regulation of the Th1/Th2 balanced immune response (Ashkar et al., 2000), protecting infants against infections by inducing a Th1 response. Indeed, breast-fed infants have been found to have greater induction of a Th1-like response after immunization against measles, mumps, and rubella, compared to formula fed infants (Pabst et al., 1997). A randomized clinical trial was recently conducted to evaluate effects of adding a bovine OPN fraction to formula. Formula fed infants were randomized to one of three formula groups: standard formula, (F0), standard formula with bovine OPN at 65 mg/L bovine OPN (F65) or standard formula with 130 mg/L bovine OPN (F130) mg/L (50% and 100% of human milk level, respectively) from 1 to 6 months of age. Growth and biological outcomes of formula fed infants were compared with a reference group of breast-fed (BF) infants. Among the formula-fed groups, the pro-inflammatory cytokine TNF-a was significantly lower in the F65 and F130 groups than in the F0 group, suggesting that OPN downregulates inflammatory cytokines in formula fed infants (Lonnerdal et al., 2016). There were no differences in growth,formula intake, or tolerance among the formula fed groups (Lonnerdal et al., 2016). In addition, several fold higher levels of no observed adverse event levels (NOAEL) for bovine osteopontin have been established using in vitro, and in vivo animal studies (Kvistgaard et al 2014).

4.5 OPO Sn-2 oil

It has long been recognized that there are differences in bowel habits and gastrointestinal (GI) symptoms in HM fed versus formula-fed (FF) infants. Infants fed HM are reported to have more frequent and softer stools than FF infants. The fat blends used in infant formulas may contribute to harder and less frequent stools reported in some FF infants. Human milk fat is characterized by high contents of palmitic and oleic acids, the former heavily concentrated in the Sn-2 position on the triglyceride molecule and the latter in the 1- and 3-positions of the triglyceride molecule (Lien, 1994). Fat absorption is higher from human milk than from current infant formulas, despite the similarities between the fatty acid profiles (Innis et al., 1994). This may partly be explained by the unique triacylglycerol structure of human milk. Palmitic acid (16:0) is abundant in human milk and is an important source of energy. Most of the 16:0 in human milk is located in the Sn-2

position of the triacylglycerol molecules, in contrast to cow's milk and vegetable oils which have 40% and 5-20%, respectively, of the 16:0 in the sn2-position. As such, hydrolysis of human milk fat result in 16:0 mainly as sn2-monoacylglycerol, which are well absorbed. Hydrolysis of fat in infant formula 16:0 from vegetable oils will be present as free fatty acids, which tend to bind calcium and form insoluble calcium-soaps in the intestine that may cause harder stools and constipation (Lien, 1994).

OPO Sn-2 oil is a structured triglyceride where palmitic acid bonded to the middle position (sn-2) of the glycerol backbone and oleic acid (18:1n-9) is bonded to the Sn 1 and 3 positions. OPO Sn-2 oil is an FDA GRAS approved oil of a proprietary blend that contains Coconut, Soybean and High Oleic Sunflower Oil. Several studies have evaluated the safety and efficacy of infant formula containing Sn-2 oils. Kennedy et al. evaluated the efficacy of a term infant formula with a higher proportion of palmitate in the sn-2 position and found that compared to standard formula, the sn-2 enriched formula resulted in reduced stool soap fatty acids, softer stools more like those of breast-fed infants, and higher whole body bone mineral content (Kennedy et al., 1999). Yao et al. evaluated the efficacy of a formula with sn-2 oil and formulas with sn-2 oil plus oligofructose versus standard infant formula and found that formula with sn-2 led to reduced stool calcium-soaps and softer stools, and the addition of oligofructose further improved stool consistency (Yao et al., 2014). Other studies have reported that infants fed high sn-2 oil formulas compared to standard formula demonstrated reduced crying duration and frequency (Litmanovitz et al., 2014).

4.6 Pre-biotics

Human milk contains an abundance of complex glycans (saccharides), that are produced by the mammary gland. Human milk oligosaccharides (HMOs) are a family of glycans that includes: glycoproteins, glycopeptides, and glycolipids (Smilowitz et al., 2014). These glycans are not digested and instead reach the large intestine intact, where they support the growth of good bacteria, enhance the intestine epithelial barrier, and bind bacteria, viruses, and toxins. Colostrum contains as much as 20–25 g/L HMO (Coppa et al., 1999) and mature milk contains 5–20 g/L HMO (Coppa et al., 1999; Davidson et al., 2004). HMOs are the third-largest component in human milk after lactose and lipids and are present at > 100-fold than are found in bovine milk (Zirkovic et al., 2011, Bode L. 2012). Due to limited availability and lack of technological advancements, alternative ingredients (prebiotics) were explored to match the functionality of HMOs. Prebiotics provide similar functional benefits as HMOs and are defined as "a selectively fermented ingredient that allows specific changes, both in the composition and/or activity in the gastrointestinal microbiota that confers benefits upon host well-being and health" (Roberfroid, 2007). Over the past decade, infant formulas with different combinations of added prebiotics have been clinically tested. These studies have found that: a mixture of galacto- and fructooligosaccharides added to term infant formula stimulated the growth of Bifidobacteria and Lactobacilli, was well tolerated, and resulted in softer stools (Moro et al., 2002); a formula with polydextrose (PDX) and galactooligosaccharides (GOS) and a formula with polydextrose (PDX), galactooligosaccharides (GOS), and lactulose (LOS), supported normal growth and resulted in softer stools (Ziegler et al., 2007); a term infant formula with polydextrose (PDX) and galacto-oligosaccharides (GOS) increased total bifidobacteria and resulted in softer stools (Scalabrin et al., 2012).

Taken together, these studies demonstrate that infant formulas with added prebiotics are well tolerated by infants and support age-appropriate growth.

4.7 Lutein

Lutein is one of the naturally occurring carotenoids contained in human milk. Carotenoids are yellow, orange, and red pigments synthesized by plants and include the compounds a-carotene, B-carotene, B-cryptoxanthin, lutein, zeaxanthin, and lycopene. Carotenoids function as antioxidants (Krinsky and Johnson, 2005), and lutein is selectively taken up into the macula of the eye where it absorbs potentially damaging blue light and helps maintain visual function (Landrum and Bone, 2001). Carotenoids cannot be synthesized in the body and therefore must be consumed in the diet. Human milk contains lutein from the mother's diet in ranges from 3 mcg/L to 232 mcg/L (Canfield et al., 2003), whereas infant formula contains much smaller and more variable amounts from trace ingredients. The safety of lutein supplement infant formula was tested in a 16-week study (Capeding et al., 2010). A total of 232 infants were randomized to one of two formulas: term infant formula or term infant formula with 200 mcg/L lutein. The lutein-supplemented formula supported age-appropriate growth and there were no significant differences in growth/weight gain or formula intake between the two groups (Capeding et al., 2010).

4.8 DHA and ARA

Long chain polyunstaturated fatty acids (LCPFUAs) such as docosahexaenoic acid (DHA) and arachidonic acid (ARA) play important roles in human biology, specifically in lipid-rich membranes of the central nervous system such as those in the eye and brain (Clandinin et al., 1980, Clandinin et al., 1980, Fleiser et al., 1983). A lack of sufficient LCPUFA intake may modify the growth and function of the central nervous system (Bourre et al., 1989, Neuringer M et al., 1989). Human milk provides the breastfed infant with DHA and ARA at mean (± SD) concentrations of 0.32 ± 0.22% and 0.47 ± 0.13% by weight for DHA and ARA, respectively (Brenna et al., 2007). Infants fed formula without added LCPUFAs have been shown to have significantly lower plasma or red blood cell concentrations of DHA and ARA compared to breastfed infants or infants fed formulas supplemented with DHA/ARA (Austead et al., 1997, Birch et al., 2000). The safety of DHA/ARA-supplemented infant formulas has been established in several randomized, double-blinded, placebo-controlled trials evaluating the efficacy of these LCPUFAs on infant cognitive function (Birch et al., 2000, Agostoni et al., 1995, O'Connor et al., 2001, Makrides et al., 2000).

5 OBJECTIVES

Primary Efficacy Objective:
Compare the growth of infants randomized to a commercially available term infant formula (Brand Formula) versus growth of infants randomized to the experimental infant formula for term infants (BBN-001 w/ OPN or BBN-102 w/o OPN).

Secondary Efficacy Objective:
Compare the formula intake volume between the formula groups.

Exploratory Objective:
Compare the markers of inflammation (tumor necrosis factor-alpha, interleukin 2, 4, 5, 6, 8, 10, 12, 13, & 17; interleukin 2 receptor, interleukin 1 beta, and interferon gamma) between the formula groups.

Primary Safety Objective:
To compare the frequency of adverse events (AEs) between the formula groups.

Secondary Safety Objective:
To compare the gastrointestinal tolerance (stool composition, bowel movements, stool consistency, gas, fussiness, and ICQ scales) between the formula groups.

6 STUDY DESIGN

6.1 Approximate Duration of Subject Participation

Subjects will participate in the study for 16 weeks.

6.2 Approximate Number of Subjects

In phase one, approximately 256 healthy term infants (128 per group, 64 per gender per group) will be enrolled to complete a minimum of 180 evaluable infants (90 per group, 45 per gender per group).

In phase two, approximately 96 healthy term infants (10 in the Brand Formula group and 86 in the BBN-102 w/o OPN group will be enrolled. The 129 subjects randomized to Brand Formula in the phase one of the study will be combined with the Brand Formula subjects in phase two to have a minimum of 139 infants in Brand Formula and 86 in BBN-102 w/o OPN . Infants will be stratified by sex to achieve balance of males and females within each formula group.

7 SELECTION OF SUBJECTS

The institutional review board (IRB) will review and approve the protocol and informed consent form for this study. Each parent/guardian must participate in the informed consent process and sign and date the current IRB-approved informed consent form for this protocol before any protocol-required procedures are performed.

7.1 Inclusion Criteria

Infants will be eligible to participate if they meet all of the following conditions. At birth the infant must be:

1. Healthy, term (no less than 37 weeks, 0 days and no greater than 42 weeks, 0 days), singleton infant
2. Have a birth weight of □ 2500 grams

At the time of the baseline/enrollment visit, infants must be:

3. Designated as healthy by a physician
4. :S 14 days post-natal age (Date of Birth = Day 0)
5. Weight for age □ 5th and :S 95th percentile for age according to World Health Organization (WHO) growth charts for infants and children ages 0 to 2 years of age
6. Length for age □ 5th and :S 95th percentile for age according to World Health Organization (WHO) growth charts for infants and children ages 0 to 2 years of age
7. Head circumference for age □ 5th and :S 95th percentile for age according to World Health Organization (WHO) growth charts for infants and children ages 0 to 2 years of age
8. Weight for length for age □ 5th and :S 95th percentile for age according to World Health Organization (WHO) growth charts for infants and children ages 0 to 2 years of age
9. Exclusively consuming and tolerating a cow's milk infant formula at time of enrollment; only infants whose parent(s) or legal guardian(s) have decided to feed infant formula as sole source of nutrition will be approached for potential study enrollment
10. Have parent(s) or legal guardian(s) who agree to feed the study formula as the sole source of nutrition for the duration of the study
11. Have parent(s) or legal guardian(s) who have read and voluntarily signed an Informed Consent form approved by the Institutional Review Board prior to any participation in the study.
12. Infants may be considered for enrollment again with a new baseline evaluation.

7.2 Exclusion Criteria

Infants will be ineligible if they have any of the following conditions that are judged by a physician to interfere with the infant's normal growth, development, and/or tolerance to an infant formula:

1. Show evidence of anatomic and physiologic defects of the respiratory tract, or other congenital defects (as determined by the clinician)
2. Show evidence of chronic hepatic, gastrointestinal, renal, cardiac, pulmonary, or neurological diseases
3. Have a maternal history with known adverse effects on the fetus and/or the newborn infant, such as diabetes (gestational diabetes is acceptable if infant's birth weight is < 4300 g), active tuberculosis, perinatal infection, or substance abuse
4. Have a family history of cow's milk protein intolerance/allergy
5. Are an infant from a multiple birth (twin, triplet, etc.)

8 PRIOR AND CONCOMITANT MEDICATION AND TREATMENT

Infants who were previously breastfed can be enrolled provided they have discontinued breastfeeding at study entry, and parents agree that they have voluntarily chosen to exclusively formula-feed the study subject during the study. If formula-fed, infants must be currently receiving and tolerating cow's milk formula. At each study visit, site personnel will interview parent(s)/guardian(s) to obtain information about all concomitant therapy that was administered since the previous study visit. Concomitant medications include prescription medications, over-the-counter medications, and herbal supplements. Routine childhood immunizations will not be recorded as concomitant medications. This information will be recorded in the subject's medical record. If the infant was administered medication or treatment for a condition that may have an effect on the infants' growth and formula tolerance, the Investigator or medical staff must assess the potential medical implications to determine whether the study subject remains eligible to continue in the study. Use of an investigational product (i.e., therapeutic drug or vaccine) during a patient's participation in the study is prohibited. Participation in an observational or non-pharmaceutical/non-interventional trial (i.e. device trial) is allowed.

If a study subject was fed a formula other than the assigned study formula, this variation from protocol must be noted on the Protocol Deviation Log.

9 PROCEDURES

9.1 Screening

Each infant will be screened for all inclusion and exclusion criteria. If an infant complies with all inclusion and exclusion criteria and the parent(s)/guardian(s) sign the IRB-approved ICF, the infant will be randomized to receive a study formula and assigned a unique subject number. If the infant does not comply with one or more of the inclusion or exclusion criteria, the infant will be defined as a screen failure.

9.2 Study Visit Procedures

Visit 1 (Day 0)

- Informed consent process
- Inclusion/Exclusion criteria
- Demography
- Randomization
- Infant feeding history
- Physical exam
- Medical history
- Maternal smoking history
- Anthropometry (see Appendix A for data collection procedures)
- Stool Characteristics and Tolerance Questionnaire
- Infant Characteristics Questionnaire (ICQ)
- 3-day Formula/Diet Record
- Telephone contact
- Medications
- Adverse events
- Dispensing of study formula

Visit 2 (Day 15 ±3 days)

- Medical History
- Anthropometry
- Stool Characteristics and Tolerance Questionnaire
- Infant Characteristics Questionnaire
- 3-day Formula/Diet Record
- Medication
- Adverse events
- Dispensing of study formula

Visit 3 (Day 30 ±3 days)

- Medical history
- Anthropometry
- Stool Characteristics and Tolerance Questionnaire
- Infant Characteristics Questionnaire
- 3-day Formula/Diet Record
- Medication
- Adverse events
- Dispensing of study formula

Visit 4: Telephone contact (Day 45 ±3 days)

- Stool Characteristics and Tolerance Questionnaire
- Medication
- Adverse events

Visit 5 (Day 60 ±3 days)

- Medical history
- Anthropometry
- Stool Characteristics and Tolerance Questionnaire
- Infant Characteristics Questionnaire
- 3-day Formula/Diet Record
- Medication
- Adverse event
- Dispensing of study formula

Visit 6: Telephone contact (Day 75 ±3 days)

- Stool Characteristics and Tolerance Questionnaire
- Medication
- Adverse events

Visit 7 (Day 90 ±3 days)

- Medical history
- Anthropometry
- Stool Characteristics and Tolerance Questionnaire
- Infant Characteristics Questionnaire
- 3-day Formula/Diet Record
- Dispense stool collection kit
- Medication
- Adverse events
- Dispensing of study formula

Visit 8: Telephone contact (Day 105 ±3 days)

- Stool Characteristics and Tolerance Questionnaire
- Medication
- Adverse events

Visit 9 (Day 120 ±3 days)

- Medical history
- Anthropometry
- Stool Characteristics and Tolerance Questionnaire
- Infant Characteristics Questionnaire
- 3-day Formula/Diet Record
- Blood Collection
- Stool collection
- Medication
- Adverse events
- Collection of all unused/partially used study formula

10 INVESTIGATIONAL PRODUCT AND ADMINISTRATION

10.1 Study Formulas

Infants will consume ad libitum per day one of the following:

1. **Brand Formula:** A commercially available infant formula for term infants; 100 kcal/5 fl. oz, 2 g protein/100 kcal (Enfamil Infant 0-12 months by Mead Johnson Nutrition, LLC)
2. **BBN-001 w/ OPN**: An infant formula for term infants containing alpha-lactalbumin enriched whey, OPO Sn-2 oil, osteopontin, lactoferrin, pre-biotics (PD, GOS, FOS), lutein, microencapsulated DHA/ARA; 100 kcal/5 fl. oz, 2.2 g protein/100kcal (Manufactured by Building Block Nutritionals, LLC)
3. **BBN-102 w/o OPN :** An infant formula for term infants containing alpha-lactalbumin enriched whey, Sn-2 oil, lactoferrin, pre-biotics (GOS, FOS), lutein, DHA/ARA; 100 kcal/5 fl. oz, 2.5 g protein/100 kcal (Manufactured by Building Block Nutritionals, LLC)

See Appendix B for details of formula nutrient composition and Appendix C for formula ingredients.

10.2 Type and Amount

Each infant will consume either Brand Formula, BBN-001 w/ OPN, or BBN-102 w/o OPN ad libitum for 16 weeks. Parent(s) or guardian(s) are discouraged from feeding their infant any foods other than the assigned study formula.

10.3 Administration

The study formula will be provided in powder form. Each of the infant formulas will be packaged in composite cans. Cans will be labeled with a unique clinical product number to mask the identity of each clinical product. Study personnel will not be aware of the identity of the products. Nutrient and stability testing will be conducted to ensure that each formula meets strict quality requirements for release. Instructions for mixing the formula to 20 kcal/oz will be given to the parent(s)/guardian(s).

10.4 Formula Storage by Investigator

Formula will be labeled for clinical trial use only and kept dry, protected from sunlight, and at room temperature (60-85° F, 16-29° C). The Principal Investigator is responsible for keeping all unassigned and returned formula in a locked storage room with controlled staff access.

10.5 Formula Storage by Parent/Guardian

The parent(s)/guardian(s) will be instructed to handle formula with clean hands. Prior to opening, the can should be cleaned. Formula may be consumed at room temperature. The parent(s)/guardian(s) will be instructed to never microwave or freeze formula. Prepared formula may be warmed in a bowl of warm water. Parents will be instructed that prepared formula not consumed by the infant can be stored in the refrigerator for no more than 24 hours, and then should be thrown away.

Parents will be instructed to return all unopened cans of formula at the completion of the study. See Appendix D for sample investigational product label.

10.6 Subject Compliance

Compliance with study feedings will be monitored approximately every two weeks throughout the study. A standard set of interview questions will be administered at each clinic visit and during telephone follow-up between clinic visits to inquire about consumption of study formula and all other feedings including other formulas.

11 SAFETY

Safety assessments will involve the monitoring and recording of all AEs and serious adverse events (SAEs), stool composition, gastrointestinal tolerance, periodic anthropometric measurements, and physical assessments. Additional safety evaluations may be performed when medically indicated in the opinion of the Investigator.

12 EFFICACY

12.1 Primary Efficacy Endpoint

Mean daily weight gain (g/d) over 16 weeks.

12.2 Secondary Efficacy Endpoints

- Anthropometric measurements (head circumference gain velocity, length gain velocity) and Z-scores (weight for age, length for age, weight for length for age, head circumference for age)
- Formula intake volume

12.3 Exploratory Endpoint

Markers of inflammation (tumor necrosis factor-alpha, interleukin 2, 4, 5, 6, 8, 10, 12, 13, & 17; interleukin 2 receptor, interleukin 1 beta, and interferon gamma).

13 LABORATORY DETERMINATIONS

Central laboratories will be used for all laboratory determinations unless a special test or emergency testing is required. The central laboratories that have been contracted to perform these tasks adhere to Good Clinical Practices and will provide supplies and shipping materials for all laboratory determinations. Refer to the central laboratory manual(s) for additional information.

13.1 Biological samples

A single blood sample (1.0 milliliters) will be collected from each subject via heel stick at the last study visit (visit 9) to assess markers of inflammation (tumor necrosis factor- alpha, interleukin 2, 4, 5, 6, 8, 10, 12, 13, & 17; interleukin 2 receptor, interleukin 1 beta, and interferon gamma). Stool samples will also be collected at the last study visit (visit 9) to assess stool composition (soap fatty acids).

Biological samples will be used exclusively for the purposes outlined in this protocol and for no other purpose.

14 STATISTICS

14.1 Statistical Methods

Analyses between cohorts (Brand Formula, BBN-001 w/ OPN, and BBN-102 w/o OPN) will occur separately depending on finalization of Generally Recognized as Safe (GRAS) approval of specific ingredients in BBN-001 w/ OPN formula.

14.1.1 Primary Efficacy Endpoint

The primary efficacy endpoint, mean daily weight gain (g/d) over a 16-week study period, will be compared between formula groups by the calculation of the 95% two-sided confidence interval on the difference between the two means. Non-inferiority will be determined if the lower limit of the two-sided 95% CI for the formula difference is greater than -3, assuming a non-inferiority margin of 3 mg/d.

Infant growth will also be descriptively summarized on the basis of the following sex-specific z- scores based on World Health Organization growth charts for infants and children ages 0 to 2 years of age: weight-for age, weight-for-length, length-for-age, and head circumference-for-age z-scores. For each subject, a line listing of all raw measures of weight, length, head circumference, and all z-scores (weight-for-age z-score, length-for-age z-score, weight-for-length z-score, and head circumference-for-age z-score) will be provided. Descriptive statistics will be used to summarize weight (kg), length (cm), and head circumference (cm) and all z-score data (weight-for age, weight-for-length, length-for-age, and head circumference-for-age) by formula group for each visit. A test for non-inferiority will also be performed for length gain (cm/d) velocity and head circumference gain (cm/d) velocity.

14.1.2 Safety Endpoints

A line listing of each subject with an adverse event will be generated. At the group level, the number and percentage of subjects having each AE will be summarized for each formula group. Secondary endpoints including stool characteristics, GI tolerance, and infant characteristics will be summarized using descriptive statistics and qualitatively compared to age appropriate reference values when appropriate.

14.1.3 General Methods

Continuous variables will be summarized using the appropriate descriptive statistics: n, mean, standard deviation, median, minimum, and maximum. The geometric mean will be presented for log-transformed

variables. Frequency and percentage of observed values will be reported for categorical measures. A line listing of all data, sorted by subject and when appropriate by time, will be generated.

Analysis Populations

- Intent to Treat (ITT): Subjects who are randomized to one of the formula feeding groups.
- Per Protocol (PP): A subset of the ITT population. It will consist of all subjects who complete the feeding protocol without major protocol violations and who consume a single non-study formula feeding no more than 10 times during the duration of 16-week study as documented on the Other Than Formula Feeding Log. Additionally, subjects who consume more than 3 complete days of non-study formula, defined as greater than 50% of the number of feedings in a 24-hour period from non-study formula, will also be excluded from the PP population.
- Safety Population: The safety population will be comprised of any subjects who are randomized and consume at least one feeding of the assigned formula.

Classification into ITT or PP populations will be conducted prior to the database lock. All listings will be provided for the ITT population. A separate listing will be included on the set of subjects who are randomized but never take any feeding formula before discontinuing from the study.

Since this study has a primary aim at demonstrating non-inferiority, the PP population will represent the primary analysis population to evaluate the treatment groups in terms of "efficacy". All clinical outcomes (primary and secondary) will be subject to analyses using both the PP and ITT population.

14.2 Statistical Power and Sample Size Considerations

In phase one, assuming a standard deviation in weight gain of 5.6 g/d (Nelson et al., 1989) and 80% power, 90 subjects per group (180 total) will be sufficient to demonstrate non-inferiority (one sided alpha = 0.025). Assuming a 25% attrition rate, a total of 256 subjects will be enrolled in this trial. Within each formula group, infants will be balanced by sex (equal number of males and females in each formula group). At the conclusion of the study, a post-hoc power analysis on weight gain will be conducted.

In phase two, the sample size for this study will be based on a non-inferiority test comparing weight gain velocity between Brand Formula and BBN-102 w/o OPN at Week 16. A non- inferiority margin of 3 g/d is set for the difference in weight gain for the two formula-fed groups (BBN-001 w/ OPN and BBN-102 w/o OPN).

A blinded interim analysis of 81 subjects who completed 16 weeks of the study was performed in phase one of the study to estimate the standard deviation of weight gain. Based on this analysis, it is assumed that the standard deviation in weight gain is 6.0 g/d. Assuming this standard deviation in weight gain of 6.0 g/d and approximately 90% power, approximately 168 (104 in the Brand Formula group and 64 in the BBN-102 w/o OPN group) will be sufficient to demonstrate non-inferiority (one-sided alpha = 0.025). Assuming a 25% attrition rate, approximately a total of 96 subjects will be enrolled in phase two of this trial.

The plan is to use the 129 subjects randomized to Brand Formula in phase one of the study, assuming a 25% attrition rate and 96 subjects meet the Per Protocol population criteria. This data has and will remain blinded through the enrollment of phase two of the study. In phase two of the study infants will be randomized to Brand Formula or BBN-102 w/o OPN in a 1:8 ratio. Assuming a 25% attrition rate and to obtain approximately 90% power, approximately 10 subjects randomized to Brand Formula and 86 subjects randomized to BBN-102 w/o OPN will be enrolled in phase two of the study. With the addition of the 129 subjects from phase one of the study, there will be approximately a total of 139 subjects enrolled in the Brand Formula group and 86 subjects enrolled in the BBN-102 w/o OPN group. Infants will be stratified by sex to achieve balance of males and females within each formula group.

14.3 Interim Analyses and Data Monitoring

In phase one, an interim analysis will be conducted in a blinded manner when 128 infants (50% of the total goal sample size) had enrolled and completed Visit 5. Weight gain velocity (g/d) for all infants were reported descriptively (minimum, maximum, mean, median, standard deviation, first and third quartiles) for the following time intervals: enrollment to Visit 3, enrollment to Visit 5, Visit 3 to Visit 5. The purpose of the interim analysis was to determine whether or not the assumed standard deviation in weight gain velocity (5.6 g/d) based on reference data by Nelson et al., 1989, was appropriate for our study population. No unblinding or inferential statistics will be performed at the interim; as such, no alpha spending procedures were required.

An interim analysis will not be performed for phase two of the study.

15 SUBJECT IDENTIFICATION

Subjects will be randomized and assigned a unique subject number. A subject number will never be reassigned or reused for any reason. The Investigator will maintain a master log linking the subject number to the subject's name. The Investigator will follow all applicable privacy laws in order to protect a subject's privacy and confidentiality. Information that could identify a subject will be masked on study material.

16 INVESTIGATIONAL PRODUCT ACCOUNTABILITY

16.1 Formula Inventory

The study site will maintain an Accountability Record of all formula received, dispensed, returned, or otherwise disposed of during the study. All dispensed and unused cans of study formula must be returned by the study subject's parent(s)/guardian(s) to the Investigator at the end of study. See Appendix E for details on investigational product accountability.

16.2 Formula Disposition

If an infant discontinues participation in the study, the remaining formula will be returned to the study site. All investigational study formula returned will be disposed of in accordance with the instructions provided by the Sponsor.

17 RANDOMIZATION

Formula-fed infants will be randomly allocated to one of the three study formulas (BBN-001 w/ OPN, Brand Formula, and BBN-102 w/o OPN) via RTSM (Randomization and Trial Supply Management) System. Randomization will be blocked by formula group and stratified by infant sex to achieve balance of males and females in each formula group.

18 ADVERSE EVENTS

18.1 Definitions

An adverse event (AE) is any untoward, undesired, or unplanned event in the form of signs, symptoms, disease, or laboratory or physiologic observations occurring in a person administered an investigational product in a clinical study. The event does not need to be causally related to an investigational product or participation in the clinical study. Any illness that a study
subject develops during the study must be recorded on the AE case report form. Whenever possible, an illness should be recorded as a diagnosis rather than a series of symptoms.

Standardized definitions for stooling, spit-up, and crying (agreed upon by the Investigators and Medical Monitor; see Appendix F) will be used to report such symptoms.

All AEs must be assigned one of the following intensity scores:

- **Mild:** Transient or mild discomfort (< 48 hours); no medical intervention/therapy required
- **Moderate**: Mild to moderate limitation in activity, some assistance may be needed; no or minimal medical intervention/therapy required
- **Severe:** Marked limitation in activity, some assistance usually required; medical intervention/therapy required, hospitalization possible
- **Serious:** please see section 19.0.

All AEs must be assigned causality. The site PI or his/her designee will make a determination about the causality or relatedness of an AE to the investigational product (i.e. study formula) using the following categories:
Related: An adverse event (AE) that has a clear temporal association with investigational product administration (e.g., within 24 hours) and there is clear evidence of a causal relationship between the investigational product and the event.

Possibly Related: An AE with a temporal association with the investigational product, but other etiologies are possible. There is a reasonable possibility that the investigational product caused the event.

Unlikely Related: An AE with a temporal association with the investigational product, but other etiologies are more likely. Relatedness to the investigational product cannot definitely be ruled out, but there is less probability that the investigational product caused the event.

Not Related: An AE with no temporal association with the investigational product or one clearly related to other etiologies such as concomitant medications or conditions, or the subject's known clinical state.

As a guideline for determining if an adverse event is related to the investigational product (investigational product related), the following questions should be considered:

- Does a reasonable causal relationship exist between the AE and the investigational product based on clinical judgment and knowledge of the treatment?
- Is there a temporal relationship between the investigational product and the appearance of the AE?
- Is there biologic plausibility for a relationship between the AE and the investigational product?
- Does the subject have an underlying medical condition or is the subject taking concomitant therapies or medications that could contribute to the AE?
- Where applicable, does the AE abate on discontinuation of the investigational product (dechallenge)?
- Where applicable, does the AE reappear on repeat exposure to the investigational product (rechallenge)?

A protocol-related adverse event is an AE occurring during a clinical study that is not related to the investigational product, but is considered by the Investigator or the Medical Monitor (or designee) to be related to the research conditions, (i.e., related to the fact that a subject is participating in the study).

19 SERIOUS ADVERSE EVENTS

A serious adverse event (SAE) is defined as an AE that:

- Results in death
- Is life-threatening, i.e., the subject was, in the opinion of the Investigator, at immediate risk of death from the event as it occurred (it does not include an event that, had it occurred in a more severe form, might have caused death)
- Results in a significant, persistent or permanent change, impairment, damage, or disruption in the subject's body function/structure, physical activities, and/or quality of life
- Requires in-subject hospitalization or prolongs hospitalization
- Is another medically significant event that, based upon appropriate medical judgment, may jeopardize the subject and may require medical or surgical intervention to prevent one of the outcomes listed above (e.g., allergic bronchospasm requiring intensive treatment in an emergency department or home, blood dyscrasias, or convulsions that do not result in hospitalization, or the development of drug dependency or drug abuse)

20 ADVERSE EVENT AND SERIOUS ADVERSE EVENT RECORDING AND REPORTING

Determination of AEs should be based on the signs or symptoms detected during the physical examination and on clinical evaluation of the subject. Adverse events (AEs) will be coded using the Medical Dictionary for Drug Regulatory Affairs (MedDRA).

AEs and SAEs will be collected from the signing of the informed consent form to the end of study visit 9. The Investigator will instruct the subject to report AEs and SAEs during this time period.

During the time period specified above, the Investigator will:

- Record all AEs and SAEs in the subject's medical record.
- Record all AEs, SAEs, and the treatment of the AE/SAE on a case report form.
- Report all SAEs to the Study Sponsor as directed and to the IRB as appropriate.

An AE/SAE's causal relationship to the product has no bearing on its reportability. The Investigator must follow up on all AEs and SAEs until the events have subsided, until values have returned to baseline, or, in case of permanent impairment, until the condition has stabilized to a level acceptable to the Sponsor/Investigator.

All SAEs and follow-up information must be reported to the Study Sponsor and clinical research organization (CRO) Medical Monitor within 24 hours after learning of the event and per instructions as included in the Investigator Site File.

A listing of AEs and SAEs by subject will be provided in the clinical study report (CSR).

21 SUBJECT DISCONTINUATION OR WITHDRAWAL

A study subject may withdraw or discontinue the study at any time and for any reason. Reasons why a subject may discontinue or be withdrawn from the study include, but are not limited to, AE, parent(s)/guardian(s) request, investigator request, etc. When a subject discontinues or is withdrawn from the study, the Investigator will complete all procedures designated for Visit 9 and notify the Sponsor.

22 INFORMED CONSENT

The informed consent form (ICF) used during the informed consent process must be the current IRB approved ICF.

Before any protocol-required procedures are performed, the parent(s)/guardian(s) must:

- Be informed of all aspects of the study.
- Be given time to ask questions and time to consider the decision to participate.
- Voluntarily agree to participate in the study.
- Sign and date an IRB-approved informed consent form.

23 PROTOCOL AMENDMENTS

Any change in the study protocol requires a protocol amendment. An investigator must not make any changes to the study protocol without IRB and Sponsor. All protocol amendments must be reviewed and approved by the IRB following the same process as the original protocol.

This study will be conducted as described in this protocol, except for an emergency situation in which the protection, safety, and well-being of the subject requires immediate intervention, based on the judgment of the Investigator (or a responsible, appropriately trained professional designated by the Investigator). In the event of a significant deviation from the protocol due to an emergency, accident, or mistake, the Investigator or designee must contact the Sponsor, or their agent, at the earliest possible time by telephone. This will allow an early joint decision regarding the subject's continuation in the study. The Investigator and the Sponsor will document this decision. The IRB/IEC will be informed of all protocol changes by the Investigator in accordance with the IRB/IEC established procedure. No deviations from the protocol of any type will be made without complying with all the IRB established procedures. Any and all protocol deviations will be reported to the IRB.

24 QUALITY CONTROL AND ASSURANCE

The Sponsor or its agent performs quality control and assurance checks on all clinical studies that it sponsors. Before enrolling any subjects in this study, the Sponsor or its agent and the Investigator will review the protocol, the electronic case report form (eCRF) and eCRF instructions, the procedure for obtaining informed consent, all protocol procedures, and the procedure for reporting AEs and SAEs. The Sponsor or its agent will monitor the conduct of the study on a periodic basis. During these site visits, information recorded in the eCRF is verified against source documents.

25 DIRECT ACCESS, DATA HANDLING, AND RECORD-KEEPING

The Investigator will permit study-related monitoring, audits, IRB review, and regulatory inspections by providing direct access to source data and documents. All information will be recorded on source documents. All required data will be recorded in the case report forms (CRFs). All CRF data must be submitted to the Sponsor throughout and at the end of the study. Electronic data capture will be used to record and transmit data electronically to the Sponsor.

The CRF data are stored in a database and processed electronically. The Sponsor Medical Monitor reviews the data for safety information. The data are reviewed for legibility, completeness, and logical consistency. Automated validation programs identify missing data, out-of-range data, and other data inconsistencies. Requests for data clarification are forwarded to the investigative site for resolution.

Periodic monitoring visits will include a review of patient records, eCRFs, and other study related documentation. While on site, the Monitor will be responsible for the following activities:

- performing source document verification (SDV) for 100% of patients at each site
- reviewing eCRFs for accuracy and completeness of information, missing data including omission of specific individual data elements and any concomitant drugs, intercurrent illness, serious adverse events, missing patient visits or examinations
- verifying (via a signature) the Investigator or an appropriate designee's review of the eCRFs

26 RECORDS RETENTION

The Investigator shall retain and preserve 1 copy of all data collected or databases generated in the course of the study, specifically including but not limited to those defined by Good Clinical Practice (GCP) as essential, for two years after completion of the study. At the end of such period, the Investigator shall notify the Sponsor in writing of his or her intent to destroy all such material. The Sponsor shall have 30 days to respond to the Investigator's notice, and the Sponsor shall have a further opportunity to retain such materials at the Sponsor's expense. The Sponsor will provide the FDA with 1 electronic copy of all data. Subject's medical files should be retained in accordance with applicable legislation and in accordance with the maximum period of time permitted by the hospital, institution or private practice. The documents can be retained for a longer period, however, if required by the applicable regulatory requirements or by agreement with the Sponsor.

To comply with these requirements, the Investigator will not dispose of any records relevant to this study without either (1) written permission from the Sponsor, or (2) providing an opportunity for the Sponsor to collect such records. The Investigator shall take responsibility for maintaining adequate and accurate hard copy source documents of all observations and data generated during this study, including the hard copy or discs received from the Sponsor of the final data. Such documentation is subject to inspection by the Sponsor or its agents, the FDA and/or other regulatory agencies.

REFERENCES

Agostoni C, Trojan S, Bellù R, Riva E, Giovannini M. Neurodevelopmental quotient of healthy term infants at 4 months and feeding practice: the role of long-chain polyunsaturated fatty acids. Pediatr Res. 1995;38(2):262–266.

Auestad N, Montalto MB, Hall RT, et al. Visual acuity, erythrocyte fatty acid composition, and growth in term infants fed formulas with long chain polyunsaturated fatty acids for one year.
Ross Pediatric Lipid Study. Pediatr Res. 1997;41(1):1–10.

Ballard O and Morrow, A. Human mik composition: Nutrient and bioactive factors. Pediatr Clin North Am. 2013;60(1):49-74.

Birch EE, Garfield S, Hoffman DR, Uauy R, Birch DG. A randomized controlled trial of early dietary supply of long-chain polyunsaturated fatty acids and mental development in term infants. Dev Med Child Neurol. 2000;42(3):174–181.

Bode L. Human milk oligosaccharides: every baby needs a sugar mama. Glycobiology 2012;22:1147–62.

Brenna JT, Varamini B, Jensen RG, Diersen-Schade DA, Boettcher JA, Arterburn LM. Docosahexaenoic and arachidonic acid concentrations in human breast milk worldwide. Am J Clin Nutr. 2007;85(6):1457-64.

Brodbeck U, Denton WL, Tanahashi N, Ebner KE. The isolation and identification of the B protein of lactose synthetase as alpha-lactalbumin. J Biol Chem. 1967; 242: 1391-1397.

Canfield LM, Clandinin MT, Davies DP, Fernandez MC, Jackson J, Hawkes J, Goldman WJ, Pramuk K, Reyes H, Sablan B, Sonobe T, Bo X. Multinational study of major breast milk carotenoids of healthy mothers. Eur J Nutr. 2003;42(3):133-41.

Capeding R, Gepanayao CP, Calimon N, Lebumfacil J, Davis AM, Stouffer N, Harris BJ. Lutein-fortified infant formula fed to healthy term infants: evaluation of growth effects and safety. Nutr J. 2010; 21; 9:22.

Cheng JB, Wang JQ, Bu DP, Liu GL, Zhang CG, Wei HY, et al. Factors affecting the lactoferrin concentration in bovine milk. J Dairy Sci. 2008;91(3):970–6.

Clandinin M, Chappell J, Leong S. Intrauterine fatty acid accretion rates in human brain: implication for fatty acid requirements. Early Hum Dev. 1980; 4:121–130.

Clandinin M, Chappell J, Leong S. Extrauterine fatty acid accretion rates in human brain: implication for fatty acid requirements. Early Hum Dev. 1980;4:131–138.

Coppa GV, Pierani P, Zampini L, Carloni I, Carlucci A, Gabrielli O. Oligosaccharides in human milk during different phases of lactation. Acta Paediatr. 1999; 88(430):S89-S94.

Davidson B, Meinzen-Derr JK, Wagner CL, Newburg DS, Morrow AL. Fucosylated oligosaccharides in human milk in relation to gestational age and stage of lactation. Adv Exp Med Biol. 2004; 554():427-30.

DHHS. (2000a, May 11, 2004). Growth chart training module. Accurately Weighing and Measuring Infants, Children and Adolescents: Technique. Health Resources and Services Agency, Maternal and Child Health Bureau. Retrieved February 3, 2017, from the World Wide Web: http://www.cdc.gov/nccdphp/dnpa/growthcharts/training/modules/modules.htm

Dupont C, Rivero M, Grillon C, Belaroussi N, Kalindjian A, Marin V. Eur J Clin Nutr. 2010 Jul;64(7):765-7.

Fleisler S, Anderson RE. Chemistry and metabolism of lipids in the vertebrate retina. Prog Lipid Res. 1983;22:79–131.

Innis SM1, Dyer R, Nelson CM. Evidence that palmitic acid is absorbed as sn-2 monacylglycerol from human milk by breast-fed infants. Lipids. 1994;29(8):541-5.

Jackson JG, Janszen DB, Lonnerdal B, Lien EL, Pramuk KP, Kuhlman CF. A multinational study of alpha-lactalbumin concentration in human milk. J Nutr Biochem. 2004; 15: 517-521.

Johnston WH, Ashley C, Yeiser M, Harris CL, Stolz SI, Wampler JL, Wittke A, and Cooper TA. Growth and tolerance of formula with lactoferrin in infants through one year of age: double- blind, randomized, controlled trial. BMC Pediatr. 2015; 15: 173.

Kennedy K1, Fewtrell MS, Morley R, Abbott R, Quinlan PT, Wells JC, Bindels JG, Lucas A. Double-blind, randomized trial of a synthetic triacylglycerol in formula-fed term infants: effect on stool biochemistry, stool characteristics, and bone mineralization. Am J Clin Nutr. 1999 Nov;70(5):920-7.

King J, Cummings G, Guo N, Trivedi L, Readmond B, Keane V, et al. A double-blind, placebo- controlled, pilot study of bovine lactoferrin supplementation in bottle-fed infants. J Pediatr Gastroentrol Nutr. 2007;44:245–51.

Krinsky NI, Johnson EJ. Carotenoid actions and their relation to health and disease. Mol Aspects Med. 2005;26(6):459-516.

Landrum JT, Bone RA. Lutein, zeaxanthin, and the macular pigment. Arch Biochem Biophys. 2001.1; 385(1):28-40.

Lien EL. The role of fatty acid composition and positional distribution in fat absorption in infants. J Pediatr. 1994;125:S62-8.

Lien EL, Davis AM, and Multicenter group. Growth and Safety of a reduced protein formula enriched with bovine alpha-lactalbumin in term infants. 2004; J Pediatr Gastroenterol Nutr. 170- 176.

Litmanovitz I, Bar-Yoseph F, Lifshitz Y, Davidson K, Eliakim A, Regev RH, Nemet D. Reduced crying in term infants fed high beta-palmitate formula: a double-blind randomized clinical trial. BMC Pediatr. 2014 Jun 19;14:152.

Lohman T, Roche AF, Martorell R. Anthropometric standardization reference manual. Champaign, IL: Human Kinetics Books; 1988.

Lonnerdal B. Digestibility and absorption of protein in infants. In: Protein Metabolism During Infancy, ed. Raiha NCR. Vevey: Raven Press. 1994; 53-65.

Lonnerdal B. Infant formula and infant nutrition: bioactive proteins of human milk and implications for composition of infant formulas. Am J Clin Nutr. 2014;99(3):712S–7S.

Makrides M, Neumann MA, Simmer K, Gibson RA. A critical appraisal of the role of dietary long-chain polyunsaturated fatty acids on neural indices of term infants: a randomized, controlled trial. Pediatrics. 2000;105(1 pt 1):32–38.

Manzoni P, Stolfi I, Messner H, Cattani S, Laforgia N, Romeo MG, Bollani L, Rinaldi M, Gallo E, Quercia M, Maule M, Mostert M, Decembrino L, Magaldi R, Mosca F, Vagnarelli F, Memo L, Betta PM, Stronati M, Farina D, Italian Task Force for the Study and Prevention of Neonatal Fungal Infections–the Italian Society of Neonatology. Bovine lactoferrin prevents invasive fungal infections in very low birth weight infants: a randomized controlled trial. Pediatrics. 2012 Jan; 129(1):116-23.

Moro G, Minoli I, Mosca M, Fanaro S, Jelinek J, Stahl B, Boehm G. Dose-related bifidogenic effects of galacto- and fructo-oligosaccharides in formula-fed term infants. J Pediatr Gastroenterol Nutr. 2002 Mar;34(3):291-5.

Nelson SE, Rogers RR, Ziegler EE, Fomon SJ: Gain in weight and length during early infancy. Early Human Development. 1989; 19 (4):223-239.

Neuringer M, Connor W, Lin D, Barstad L, Luck S. Biochemical and functional effects of prenatal and postnatal ro-3 fatty acid deficiency on retina and brain in rhesus monkeys. Proc Natl Acad Sci. 1989;83:285–294.

Ochoa TJ, Cleary TG. Effect of lactoferrin on enteric pathogens. Biochimie. 2009;91(1):30–34.

O'Connor DL, Hall R, Adamkin D, et al. Ross Preterm Lipid Study Growth and development in preterm infants fed long-chain polyunsaturated fatty acids: a prospective, randomized controlled trial. Pediatrics. 2001;108(2):359–371

Roberfroid M. Prebiotics: the concept revisited. J Nutr. 2007;137(3 Suppl 2):830S–837S.

Scalabrin DM, Mitmesser SH, Welling GW, Harris CL, Marunycz JD, Walker DC, Bos NA, Tölkkö S, Salminen S, Vanderhoof JA. New prebiotic blend of polydextrose and galacto- oligosaccharides has bifidogenic effect in young infants. J Pediatr Gastroenterol Nutr. 2012; 54(3):343-52.

Schack L, Lange A, Kelsen J, Agnholt J, Christensen B, Petersen TE, et al. Considerable variation in the concentration of osteopontin in human milk, bovine milk, and infant formulas Journal of Dairy Science. 2009; 92:5378–5385.

Smilowitz JT, Lebrilla CB, Mills DA, German JB, Freeman SL. Breast milk oligosaccharides: structure-function relationships in the neonate. Ann Rev Nutr. 2014; 34: 143–169.

Trabulsi J, Capeding R, Lebumfacil J, Ramanujam K, Feng P, McSweeney S, Harris B, DeRusso P. Effect of an a-lactalbumin-enriched infant formula with lower protein on growth. Eur J Clin Nutr. 2011; 65(2):167-74.

Yao M, Lien EL, Capeding MRZ, Fitzgerald M, Ramanujam K, Yuhas R, Northington R, Lebumfacil J, Wang L, DeRusso PA. Effects of term infant formulas containing high sn-2 palmitate with and without oligofructose on stool composition, stool characteristics, and bifidogenicity. J Pediatr Gastroenterol Nutr. 2014; 59(4): 440–448.

Ziegler E1, Vanderhoof JA, Petschow B, Mitmesser SH, Stolz SI, Harris CL, Berseth CL. Term infants fed formula supplemented with selected blends of prebiotic grow normally and have soft stools similar to those reported in breast-fed infants. J Pediatr Gastroenterol Nutr. 2007 Mar;44(3):359-64.

Appendix A: Anthropometry Data Collection Procedures

All sites will be trained on standardized techniques for the measure of weight, length, and head circumference (Lohman, 1988).

Weight
Two people are involved with infant weight measures. One measurer will weigh the infant and read the weight as it is obtained. The other measurer will immediately note the measurement in the infant's chart.

The infant's clothing and diaper are removed. The infant should be positioned in the center of the scale tray. Infants should be weighed to the nearest 10 grams on a calibrated scale. Record the weight as soon as it is completed. Then the infant should be re-positioned and the weight measurement repeated and noted in writing. After the infant is removed from the scale tray, the weights should be compared, and they should agree within 10 grams. If the difference between the weights exceeds the tolerance limit of 10 grams, the infant should be re-positioned and reweighed a third time. The two weights that are within 10 grams of one another should be recorded. If there are NOT two weights within 10 grams of one another, instrument error may be influencing the measure; the Investigator should calibrate the scale, check the position of the scale and infant, etc. and then re-weigh the infant.

Summary:

- Remove infant's clothing and diaper
- Center infant on the scale tray
- Weigh infant to nearest 10 grams
- Write the weight on the infant's chart
- Reposition and repeat weighing infant
- Compare weights
- Weight should agree within 10 grams (tolerance of the measure)

Length
Length will be measured in the recumbent position with a calibrated lengthboard. The lengthboard must have 1) a fixed headpiece and 2) a moveable foot piece, which is perpendicular to the surface of the table that the length board is on.

Length measurements for infants should be obtained while the infant is dressed in light underclothing or a diaper. The infant's shoes must be removed. Hair ornaments should be removed from the top of the head.

The infant should be placed on his/her back in the center of the lengthboard so that the infant is lying straight and his/her shoulders and buttocks are flat against the measuring surface. The infant's eyes should be looking straight up. Both legs should be fully extended and the toes should be pointing upward with feet flat against the foot piece.

Two people must be involved with infant length measures. One measurer holds the infant's head, with the infant looking vertically upward and the crown of the head in contact with the headpiece in the Frankfort Horizontal Plane. The head of the infant is firmly but gently held in position. The measurer gently cups the infant's ears while holding the head in proper alignment. Make sure the infant's chin is not tucked in against his chest or stretched too far back.

While one measurer holds the infant's head in the proper position, the second measurer aligns the infant's trunk and legs, extends both legs, and brings the footpiece firmly against the heels. The measurer places one hand gently but firmly on the infant's knees to maintain full extension of the legs. The infant's toes are pointing upward with the soles of the feet perpendicular to the horizontal backpiece of the measuring device. It is imperative that both legs are fully extended for an accurate and reproducible length measurement.

The measurer at the feet should read aloud to the recorder the length measurement to the nearest 0.1 cm. The length should be recorded on the data form as soon as it is completed. Then the infant should be repositioned and the length measurement repeated and noted in writing. After the infant is removed from the lengthboard, the length measurements should be compared and they should agree within 0.5 cm.

If the difference between the two length measures exceeds 0.5 cm, the infant should be repositioned and remeasured a third time. The third measure of length should be within 0.5 cm of either the first or second length measure; and the two measures within 0.5 cm should be recorded. If there are NOT two measures of length within 0.5 cm of one another, instrument error may be influencing the infantometer; the Investigator should calibrate the infantometer, re-position the infant and re-measure the infant. The two measures that are within 0.5cm of one another should be recorded.

Summary:

- Use a calibrated lengthboard with a fixed headpiece and movable footpiece which is perpendicular to the surface of the table
- Measure infant without shoes and wearing light underclothing or diaper
- Measure length to 0.1 cm
- Record measurement on chart
- Reposition and remeasure infant
- Measurements should agree to 0.5 cm

Head Circumference

The goal of the head circumference measure is to locate the maximum circumference of the head. Head circumference or OFC (occipital frontal circumference) is measured over the most prominent part on the back of the head (occiput) and just above the eyebrows (supraorbital ridges), i.e. the largest circumference of the head. Any braids, barrettes, or other hair decorations that will interfere with the measurement should be removed. The infant may be held in the arms or on the lap of the parent/guardian if they prefer.

The tape is positioned across the frontal bones just above the eyebrows, above the ears, and around the biggest part of the back of the head (the occiput). The goal is to locate the maximum tissues. The measurement is read to the nearest 0.1 cm and recorded on the chart. The tape should be repositioned and the head circumference re-measured. The measures should agree within 0.2 cm. If the difference between the measures exceeds 0.2 cm, the infant should be repositioned and re-measured a third time. The two measures that are within 0.2 cm of one another should be recorded.

Summary:

- Use a flexible, non-stretchable tape
- The goal is to locate the maximum circumference of the head
- Position the tape just above the eyebrows on the supraorbital ridge, above the ears, and around the biggest part on the back of the head (the occiput)
- Pull tape snugly to compress the hair
- Read the measurement to the nearest 0.1 cm
- Write measurement on the chart
- Reposition tape and re-measure the head circumference
- Measures should agree within 0.2 cm

Adapted from: Department of Health and Human Services, 2000

Appendix B: Formula Composition

	**Enfamil Infant 0-12 months (Brand Formula)**	**Building Block Nutritionals (BBN-001 w/ OPN)**	**Building Block Nutritionals (BBN-102 w/o OPN)**
**Nutrients/100 calories**			
Protein, g	2	2.2	2.5
Fat, g	5.3	5.4	5.1
OPO Sn-2 oil	*	0.7	0.7
Carbohydrate, g	11.3	10.7	10.5
Dietary Fiber, mg	**	130.2	330
Galactoligosaccharide, mg	*	29.5	300
Polydextrose, mg	**	30	*
Fructoligosaccharide, mg	*	70.7	30
Linoleic Acid, mg	780	850	850
**Vitamins:**			
Vitamin A, IU	300	300	300
Vitamin D, IU	60	60	60
Vitamin E, IU	2	2	2
Vitamin K, mcg	9	9	9
Vitamin B1, mcg	80	80	80
Vitamin B2, mcg	140	140	140
Vitamin B3, mcg	1000	1000	1000
Vitamin B6, mcg	60	60	60
Vitamin B12, mcg	0.3	0.3	0.3
Folic acid, mcg	16	16	16
Pantothenic Acid, mcg	500	600	600
Biotin, mcg	3	3	3
Vitamin C, mg	12	12	12
**Minerals:**			
Calcium, mg	78	78	78
Phosphorus, mg	43	43	43
Magnesium, mg	8	8	8
Iron, mg	1.8	1.8	1.8
Zinc, mg	1	1.0	1
Manganese, mcg	15	15	15
Copper, mg	75	75	75
Iodine, mcg	15	10	15
Sodium, mg	27	27	27
Potassium, mg	108	108	108
Chloride, mg	63	63	63
Selenium, mcg	2.8	2.8	2.8
**Other:**			
Choline, mg	24	24	24
Inositol, mg	6	6	6

	**Enfamil Infant 0-12 months (Brand Formula)**	**Building Block Nutritionals (BBN-001 w/ OPN)**	**Building Block Nutritionals (BBN-102 w/o OPN)**
B-carotene, mcg	*	19	19
Lutein, mcg	*	18	10
Nucleotides, mg	**	3.9	4
L-Carnitine, mg	**	1.4	1.3
Taurine, mg	**	5.8	6
Lactoferrin, mg	*	18	9
Docosahexaenoic acid (DHA) mg	**	7.8	9.6
Arachidonic acid (ARA) mg	**	9.7	19
Data from Enfamil™ Can Label Lot Number: ZP9L4H Expiration: 1 JUN 21<br>*Not Added to Formula<br>**Nutritional Information not available			

Label for Enfamil™ 0-12 months

Appendix C: Formula Ingredients

Brand Formula
Nonfat Milk, Lactose, Vegetable Oil (Palm Olein, Coconut, Soy and High Oleic Sunflower Oils), Whey protein concentrateand and less than 2%: Galactoligosaccharides*, Polydextrose*, Mortierella Alpina Oil, Schizochytrium Sp. Oil, Calcium Carbonate, Calcium Phosphate, Potassium citrate, Ferrous sulfate, Potassium chloride, Magnesium oxide, Sodium chloride, Zinc sulfate, Cupric Sulfate, Manganese sulfate, Potassium Iodide, Sodium Selenite, Soy lecithin, Choline chloride, Ascorbic acid, Niacinamide, Calcium Pantothenate, Vitamin A palmitate, Vitamin B12, Vitamin D3, Riboflavin, Thiamin Hydrochloride, Vitamin B6 Hydrochloride, Folic acid, Vitamin K1, Biotin, Inositol, Vitamin E acetate, Taurine and L-Carnitine.
*A Type of Prebiotic
**A Source of Arachidonic acid (ARA)
**A Source of Docosahexaenoic acid (DHA)

BBN-001 w/ OPN Nonfat Milk, Lactose, Vegetable Oil (OPO Sn-2 Oil, Coconut, Soy and High Oleic Sunflower Oils), Demineralized Whey, Whey protein concentrate, Alpha lactlbumin, and less than 1% Polydextrose*, Galactoligosaccharides*, Fructooligosaccharides*, Mortierella Alpina Oil Powder** Crypthecodinium Cohnii Oil Powder** Calcium Carbonate, Dicalcium Phosphate, Potassium citrate, Ferrous sulfate, Potassium chloride, Potassium Iodide, Magnesium chloride, DiMagnesium phosphate, Sodium citrate, Zinc sulfate, Copper Sulfate, Manganese sulfate, Sodium Selenite, Soy lecithin, L-Choline bitartrate, Ascorbic acid, Niacinamide, Calcium Pantothenate, Vitmin A palmitate, Vitamin B12, Vitamin D3, Riboflavin, Thiamin Hydrochloride, Vitamin B6 Hydrochloride, Folic acid, Vitamin K1, Biotin, Inositol, Vitamin E acetate, Nucleotides (Cytidine 5'-monophosphate, Disodium Uridine 5'-monophosphate, Adenosine 5'-monophosphate,Disodium Guanosine 5'-monophosphate), Beta carotene, Lutein, Osteopontin, Lactoferrin, Taurine and L-Carnitine
*A Type of Prebiotic
**A Source of Arachidonic acid (ARA)
**A Source of Docosahexaenoic acid (DHA)

BBN-102 w/o OPN
Nonfat Milk, Lactose, Vegetable Oil (OPO Sn-2 Oil, Coconut, Soy and High Oleic Sunflower Oils), Demineralized Whey, Whey protein concentrate, Alpha lactlbumin, and less than 1%; Galactoligosaccharides*, Fructooligosaccharides*, Mortierella Alpina**, Crypthecodinium Cohnii*** Calcium Carbonate, Calcium Phosphate, Potassium citrate, Ferrous sulfate, Potassium chloride, Potassium Iodide, Magnesium chloride, Magnesium phosphate, Sodium citrate, Zinc sulfate, Copper Sulfate, Manganese sulfate, Sodium Selenite, Soy lecithin, Choline bitartrate, Ascorbic acid, Niacinamide, Calcium Pantothenate, Vitamin A palmitate, Vitamin B12, Vitamin D3, Riboflavin, Thiamin Hydrochloride, Vitamin B6 Hydrochloride, Folic acid, Vitamin K1, Biotin, Inositol, Vitamin E acetate, Maltodextrin, Nucleotides (Cytidine 5'-monophosphate, Disodium Uridine 5'-monophosphate, Adenosine 5'-monophosphate,Disodium Guanosine 5'-monophosphate), Beta carotene, Lutein, Lactoferrin, Taurine and L-Carnitine
*A Type of Prebiotic
**A Source of Arachidonic acid (ARA)
***A Source of Docosahexaenoic acid (DHA)

Appendix D: Sample Formula Labels

Appendix E: Investigational Product Accountability

The initial IP shipment is initiated by BBN upon receipt of the required site regulatory documentation. Resupply of IP to each clinical study site is based on current site inventory and projected needs.

The responsible site designee is to document the receipt of each shipment of IP from BBN by recording the date of receipt and verifying the usability of the IP. All cases of IP must be opened to verify that all IP cans are intact and are numbered with the same number as on the case. Any cases/cans damaged in shipment will not be available to dispense and will be reported to Paidion upon receipt.

Each IP case dispensed to a study subject is documented by recording the date of the dispensing and the number of the case that is dispensed. All unused cans of IP are to be returned to the site by the parent/legal guardian at the end of the study and retained by the site until return shipment instructions and/or destruction permission is granted by the Sponsor or authorized representative.

Appendix F: Standardized definitions for common Adverse Events

These AEs may or may not be related to formula tolerance (stooling, spit-up, crying, skin issues).

All AEs must be assigned an intensity score and causality (related or not related to the investigational product; see **section 18.1** for details).

ADVERSE EVENT	DEFINITION
**Stooling Issues**	
Difficulty having bowel movement	Crying, fussing, or turning red when having a bowel movement
Hard stools	Healthcare professional diagnosis; pellet or hard rock-like stools
Constipation	Less than 3 bowel movements in 7 days
Acute diarrhea	Runny or watery stools for less than 2 weeks
Chronic diarrhea	Runny or watery stools for more than 2 weeks or □ 3 separate episodes of acute diarrhea in 2 weeks
**Spit-up, Vomiting, GERD issues**	
Regurgitation	Milk comes up into mouth but never out of mouth AND infant DOES NOT: arch his/her back as if in pain, stop drinking even if hungry, or cry, wheeze, or cough related to feedings
Infantile spit up	Milk comes out of the mouth after feeding (typically non-forceful), and the amount that comes out is less than half of the feeding volume; non-projectile
Vomiting	Milk comes out of the mouth after feeding (typically forceful), and the amount that comes out is more than half of the feeding volume
Gastroesophageal Reflux Disease (GERD)	Baby arches his/her back as if in pain, stops drinking even if hungry, or cries, wheezes or coughs related to feedings (with or without milk coming up into mouth)
**Crying issues**	
Crying/Neonatal abnormal crying	Infant cries for 3 or more hours per day
Infantile colic/Infant colic	Infant cries inconsolably for 3 or more hours per day, at least 3 days per week, AND for at least 3 weeks
**Skin issues**	
Diaper rash	Contact/irritant dermatitis in the diaper area, with erythema and/or skin breakdown on the exposed convex skin surface (rate as Mild, Moderate, Severe using definitions outlined below)<br>**Mild**: Baby has an area of pinkness in the diaper area<br>**Moderate**: Baby has definite pinkness in a large area with some small areas of definite redness

	**Severe**: Baby has intense redness over a large area of the perianal region, or any area of redness with skin breakdown in the diaper area
Atopic dermatitis/Eczema	Any new skin lesions, not in the diaper area. Score for any erythema, edema/papulation, and/or excoriation on scale from 0-3 each (9 points max).<br>For each item, 0 = none, 1 = mild, 2 = moderate, 3 = severe.<br>**Mild:** 1 or 2 points<br>**Moderate:** 3 to 5 points<br>**Severe:** 6 to 9 points

Appendix G: Formula Preparation Instructions

This formula preparation instruction is appropriate for all Study Infant Formulas.

Proper hygiene, study formula preparation, dilution, use and storage are important to the well- being of your baby.

Ask your baby's doctor about the need to use cooled, boiled water when preparing the study formula and whether you need to boil utensils, bottles, nipples and rings in water before each use. If you are concerned about lead or other harmful substances in your water, talk to your healthcare professional before making study formula with tap water.

HOW TO MIX BBN-IF-001 STUDY INFANT FORMULAS:

For proper mixing, follow these steps:

1. Wash your hands thoroughly with soap and warm water.
2. Following the measurement guidelines below, add the appropriate amount of water and scoops of study formula powder to the bottle:

Measure water	Add scoop(s) of unpacked level powder using enclosed scoop	Finished bottle (approx.)
2 fl oz	1 scoop	2 fl oz
4 fl oz	2 scoops	4 fl oz
6 fl oz	3 scoops	6 fl oz
8 fl oz	4 scoops	8 fl oz

3. Put the cap on the bottle and shake.
4. Feed prepared study formula immediately (within 2 hours of preparation) or cover and store in the refrigerator for no longer than 24 hours.
   Warning: Do Not Use a Microwave to Warm Formula. Serious Burns May Result

Appendix H: Version History and Summary of Changes

Protocol	Version	Date
BBN-IF-001	1.0	27 July 2017
BBN-IF-001	2.0	09 August 2017
BBN-IF-001	3.0	14 February 2018
BBN-IF-001	4.0	02 September 2020
BBN-IF-001	5.0	25 September 2020
BBN-IF-001	5.1	06 November 2020
BBN-IF-001	5.2	07 January 2021

Clinical protocol BBN-IF-001 (version 5.1 06 November 2020) has been amended to update the serum markers of inflammation from a secondary efficacy endpoint to an exploratory endpoint, clarify that the 3-day telephone contacts that fall on a holiday or weekend can be performed at the next available business day, clarify that use of an investigational product during a subject's participation in the study is prohibited and correct the blood sample volume to 1.0 milliliters.

Changes in grammar, spelling, punctuation, format and minor editorial changes are not enumerated in this summary of changes. The most relevant changes are in the table below

SECTIONS	DESCRIPTION OF CHANGES
1.0 Protocol Synopsis, 5.0 Objectives, 12.0 Efficacy	Updated comparison of serum markers of inflammation between formula groups from a secondary efficacy endpoint to an exploratory endpoint to match the planned statistical analysis.
2.0 Study Flowchart	Added following statement to footnote 5: "In the event a 3-day telephone contact falls on a holiday or weekend, the contact will be made on the next available business day."
8.0 Prior and Concomitant Medication and Treatment	Added following statement: "Use of an investigational product (i.e., therapeutic drug or vaccine) during a patient's participation in the study is prohibited. Participation in an observational or non-pharmaceutical/non-interventional trial (i.e. device trial) is allowed.
13.0 Laboratory Determinations	Corrected the blood sample volume wording from "A single blood sample (of at least 0.5 milliliters but no more than 0.8 milliliters)…" to "A single blood sample (1.0 milliliters)…" .

DocuSign Envelope ID: 09C751E8-FA45-44F5-939E-B2F82C69093A

Statistical Analysis Plan

Protocol Title	**Growth and Safety Study of an Infant Formula for Healthy Term Infants**
Protocol Numbers	**BBN-IF-001**
Version:	2.0
Date:	16 October 2019
Sponsor:	Building Block Nutritionals, LLC<br>200 Garrett St # S<br>Charlottesville, VA 22902
Authors	Ryan McBride<br>Instat Services

This study will be conducted in compliance with the protocol, Good Clinical Practice and all other applicable regulatory requirements, including the archiving of essential documents.

DocuSign Envelope ID: 09C751E8-FA45-44F5-939E-B2F82C69093A

Document History

Version Number	Author	Date	Change
V1	Ryan McBride	12 Sep 2019	Version 1; NA
V2	Ryan McBride	16 Oct 2019	Explanation of multiple testing procedure for secondary efficacy endpoints. Inflammation markers moved to exploratory analysis.

Signatures / Approvals

	Ryan McBride	10/22/2019
Biostatistician, Instat Services	Name (Print or Type)	Date
	James McGrath	10/22/2019
CEO, BBN Nutritionals	Name (Print or Type)	Date

DocuSign Envelope ID: 09C751E8-FA45-44F5-939E-B2F82C69093A

Table of Contents

Document History....2
Signatures / Approvals....2
Table of Contents....3
List of Abbreviations....5
1. Introduction....6
2. Study Rationale and Objectives....6
2.1. Study Rationale....6
2.2. Study Objectives....6
2.2.1. Primary Efficacy Objective....6
2.2.2. Secondary Efficacy Objective....7
2.2.3. Exploratory Objective....7
2.2.4. Primary Safety Objective....7
2.2.5. Secondary Safety Objective....7
3. Study Design....7
4. FLOWCHART....8
4.1. Study Flowchart....8
5. Determination of Sample Size....9
6. Statistical Methods....9
6.1. Reporting conventions....10
6.2. Handling of repeat and unscheduled assessments....11
7. Analysis Populations....11
8. Subject Characteristics....12
8.1. Subject Disposition....12
8.2. Demographic and Baseline Characteristics....12
8.3. Protocol Deviations....12
9. Efficacy Analysis....13
9.1. Baseline Values....13
9.2. Handling of Dropouts or Missing Data....13
9.3. Multiple Testing Procedure....13
9.4. Interim Analysis and Data Monitoring....14
10. Methods of Efficacy Analysis....14

DocuSign Envelope ID: 09C751E8-FA45-44F5-939E-B2F82C69093A

10.1. Primary Efficacy Analysis 14
10.2. Secondary Efficacy Analysis 15
10.2.1. Anthropometric measurements 15
10.2.2. Sex-specific Z-scores 16
10.2.3. Formula Intake Volume 17
10.3. Exploratory Analysis 17
10.3.1. Markers of Inflammation 17
11. Pharmacokinetic Analysis 18
12. Safety Analysis 18
12.1. Gastrointestinal characteristics 18
12.2. Adverse Events 20
12.2.1. Adverse Events of Special Interest 21
12.3. Physical Examination 22
12.4. Prior and Concomitant Medications 22
12.5. Planned Tables, Figures, and Listings 24
12.5.1. Planned Tables and Figures 24
12.5.2. Planned Listings 26
13. References 27
Appendix A: Infant Characteristics Questionnaire 28

DocuSign Envelope ID: 09C751E8-FA45-44F5-939E-B2F82C69093A

List of Abbreviations

Abbreviation	Term
AE	Adverse Event
ARA	Arachidonic acid
ATC Class	Anatomical/Therapeutic/Chemical Class
BBN	Building Block Nutritionals, LLC
CFR	Code of Federal Regulations
CRF	Case report form
CI	Confidence Interval
DHA	Docosahexaenoic acid
CSR	Clinical Study Report
EDC	Electronic Data Capture
EOS	End of Study
EOSI	Events of Special Interest
Formula A	Commercially Available Milk Infant Formula (Enfamil Premium® 0-12 months)
Formula B	Experimental Formula (Building Block Nutritionals, LLC)
FOS	Fructo-oligosaccharide
GCP	Good Clinical Practice
GI	Gastrointestinal
GOS	Galacto-oligosaccharide
GRAS	Generally recognized as safe
HM	Human milk
ICF	Informed Consent Form
ICQ	Infant Characteristics Questionnaire
IEC	Independent ethics committee
IP	Investigational product
IRB	Institutional Review Board
ITT	Intent to Treat (population)
MedDRA	Medical Dictionary for Regulatory Activities
MMRM model	Mixed Model, Repeat Measures model
PP	Per-Protocol (population)
PT	Preferred Term
RTF	Rich Text Format
SERT	Safety Evaluation of Response Treatment
SOC	System Organ Class
TEAE	Treatment Emergent Adverse Event
TFL	Tables, Figures, Listings
WHO	World Health Organization

1. Introduction

Building Blocks Nutritionals (BBN), LLC has developed an infant formula for term infants that more closely resembles the composition of human milk than other commercially available infant formula products. The purpose of this randomized, controlled, double-blind study is to demonstrate that the BBN formulation meets nutritional requirements and supports age appropriate growth of healthy term infants. The study background, design and subject assessments are described in the study protocol, BBN-IF-001.

The statistical methods to be implemented during the analyses of data collected within the scope of this study will be outlined in this document. The purpose of this plan is to provide specific guidelines from which the statistical analysis will proceed. Any deviations from this plan will be documented in the clinical study report.

2. Study Rationale and Objectives

2.1. Study Rationale

Human milk (HM) is universally considered the gold standard for infant feeding. HM is a dynamic, multi-faceted fluid containing nutrients and bioactive factors needed for infant health and development. If an infant cannot be breastfed, the American Academy of Pediatrics recommends infant formula as the next best feeding alternative. A goal of infant formula development is to mimic HM in both nutrient composition as well as physiologic outcomes. For years, infant formulas have not been able to provide many of the bioactive factors found in HM. With a goal of providing infants who receive infant formula a nutritional product closer in composition to HM, improvements in infant formula composition are warranted. **The current study is designed to confirm that an infant formula for term infants that contains osteopontin alpha- lactalbumin enriched whey, OPO Sn-2 oil, lactoferrin, prebiotics, lutein, microencapsulated DHA/ARA, designed to more closely match breast milk, will support growth in healthy, term infants.**

2.2. Study Objectives

2.2.1. Primary Efficacy Objective

The primary objective of this study is to compare the growth of infants randomized to the experimental infant formula for term infants (Formula B) versus growth of infants randomized to a commercially available term infant formula (Formula A).

DocuSign Envelope ID: 09C751E8-FA45-44F5-939E-B2F82C69093A

2.2.2. Secondary Efficacy Objective

The secondary efficacy objectives of this study is to compare formula intake volume between the formula groups (Formula A vs Formula B).

2.2.3. Exploratory Objective

The exploratory objective is to compare the markers of inflammation (tumor necrosis factor-alpha, interleukin 2, 6, and 10) between the formula groups (Formula A versus Formula B).

2.2.4. Primary Safety Objective

The primary safety objective is to compare the frequency of adverse events (AEs) between the formula groups (Formula A versus Formula B).

2.2.5. Secondary Safety Objective

The secondary safety objectives of this study are:

- Gastrointestinal tolerance (stool composition, bowel movements, stool consistency, gas, fussiness, and ICQ scales)

3. Study Design

This study is a randomized, controlled, double-blind, study of healthy term formula fed (FF) infants. FF infants will be randomized to receive either a new infant formula formulated for healthy term infants (Formula B), or a commercially available infant formula for healthy term infants (Formula A). Infants will consume the formula for a total of 16-weeks; infant growth, serum markers of nutritional status, and tolerance to the formulas will be assessed throughout the four-month study period.

The study duration from Day 1 through the EOS visit is approximately 120 days. Each infant will be screened for all inclusion and exclusion criteria. If an infant complies with all inclusion and exclusion criteria and the parent(s)/guardian(s) sign the IRB-approved Informed Consent Form (ICF), the infant will be randomized to receive a study formula and assigned a unique subject number. If the infant does not comply with one or more of the inclusion or exclusion criteria, the infant will be defined as a screen failure.

Nine visits are scheduled at 15-day intervals with at 3-day visit window.

A flowchart of the study visits in included on the following page.

4. FLOWCHART

Shaded columns indicate visit occurs at study site

4.1. Study Flowchart

**Study Visit**	**1**	**2**	**3**	**4**	**5**	**6**	**7**	**8**	**9**
**Study Day**	**0**	**15** ±3 days	**30** ±3 days	**45** ±3 days	**60** ±3 days	**75** ±3 days	**90** ±3 days	**105** ±3 days	**120** ±3 days
Informed consent	X								
Inclusion/Exclusion criteria	X								
Demography	X								
Randomization	X								
Infant feeding history	X								
Physical Exam	X								
Medical History	X	X	X		X		X		X
Anthropometry[1]	X	X	X		X		X		X
Stool Characteristics and Tolerance Questionnaire	X	X	X	X	X	X	X	X	X
Infant Characteristics Questionnaire (ICQ)	X	X	X		X		X		X
3-day Formula/Diet Record[2]	X	X	X		X		X		X
Blood Collection[3]									X
Dispense stool collection kit[4]							X		
Stool Collection[4]									X
Telephone contact[5]	X			X		X		X	
Concomitant Medications	X	X	X	X	X	X	X	X	X
Adverse events	X	X	X	X	X	X	X	X	X
Dispense and collect study formula	X	X	X		X		X		X

[1]Anthropometry includes assessment of weight, length, and head circumference (see Appendix C in protocol for procedure details).

[2]Initial 3-day Formula/Diet Record will be recorded for 3 days following the first study visit. Remainder of 3-day Formula/Diet Records will be recorded for 3 days prior to each subsequent scheduled study visit.

[3]Blood collection to assess markers of inflammation (tumor necrosis factor-alpha, interleukin 2, 4, 5, 6, 8, 10, 12, 13, & 17; interleukin 2 receptor, interleukin 1 beta, and interferon gamma) and albumin.

[4]Stool collection to assess stool composition. Stool collection supplies will be distributed to parents at Visit 7 for collection of all stools in the 3 days just before Visit 9. The stool collection kit is returned at Visit 9.

[5]Initial telephone contact will occur 3 days after enrollment to check compliance with study feeding and inquire about subject well-being. Remainder of telephone contacts will occur mid-way between clinic visits. Telephone contact notes will be recorded in the study subject's medical record.

DocuSign Envelope ID: 09C751E8-FA45-44F5-939E-B2F82C69093A

5. Determination of Sample Size

Approximately 256 healthy term infants (128 per group, 64 per gender per group) will be enrolled to complete a minimum of 180 evaluable infants (90 per group, 45 per gender per group).

Formula fed infants will be randomly allocated to one of the two study formulas (Formula A or B) via IWRS (Interactive Web-Based Randomization System). Randomization will be blocked by formula group and stratified by infant sex to allow for an equal number of males and females in each formula group.

Sample size estimation is based on the primary endpoint, weight gain velocity (g/d) over a 16-week study period.

Assuming a standard deviation in weight gain of 5.6 g/d (Nelson et al., 1989) and 90% power, 90 subjects per group (180 total) will be sufficient to demonstrate equivalence (one sided alpha =0.025). Assuming a 25% attrition rate, a total of 256 subjects will be enrolled in this trial, 128 males and 128 females.

Based on discussions with regulatory agencies and internal discussions we have decided to base the results on non-inferiority. A non-inferiority margin of 3 g/d is set for the difference in weight gain for the two formula-fed groups. Based on a standard deviation in weight gain of 6.0 g/d from the interim analysis and 90 subjects per group (180 total), the power for non-inferiority is greater than 90% and is sufficient for the analyses planned.

At the conclusion of the study, a post-hoc power analysis on weight gain will be conducted.

6. Statistical Methods

The statistical analyses will be reported using summary tables, figures and listings (TFLs). Numbering for TFLs will be based on the recommended numbering convention provided by the International Conference on Harmonization. Unless noted otherwise, all statistical tests will be two-sided with a significance level of $\alpha = 0.05$. Tests will be declared statistically significant if the calculated p-value is } 0.05. Continuous variables will be summarized with means, standard deviations, medians, minimums and maximums. Categorical variables will be summarized by counts and percent of subjects

DocuSign Envelope ID: 09C751E8-FA45-44F5-939E-B2F82C69093A

in corresponding categories. Missing values are not considered for percent calculations, unless stated otherwise. In those cases, footnotes will specify the percent basis. All summary tables will be presented by formula received. Select baseline tables may also include a total summary.

Individual subject data obtained via the electronic data capture (EDC) system and from external vendors will be presented in by subject listings.

The analyses described in this plan are considered *a priori*, in that they have been defined prior to the blind being broken and the database locked. Any analysis performed after breaking the blind will be considered post-hoc and exploratory. Post-hoc analyses will be labeled as such on the output and identified in the CSR.

All analyses and tabulations will be performed using SAS® version 9.3 or higher. Tables and listings will be presented in RTF format. Upon completion, all SAS programs will be validated by an independent programmer. The validation process will be used to confirm that statistically valid methods have been implemented and that all data manipulations and calculations are accurate. Checks will be made to ensure accuracy, consistency with this plan, consistency with tables and consistency between tables and corresponding data listings.

6.1. Reporting conventions

P-values ≥0.0001 will be reported to 4 decimal places; P-values less than 0.0001 will be reported as "<0.0001". Upper case is used for P-value. The mean, standard deviation (SD), and any other statistics other than quantiles, will be reported to one decimal place greater than the original data. Quantiles, such as median, Q1, Q3 or minimum (Min) and maximum (Max) will use the same number of decimal places as the original data. Estimated parameters, not on the same scale as raw observations (e.g. regression coefficients) will be reported to 3 significant figures. Confidence limits (abbreviated CLs) will be reported as Confidence Intervals (abbreviated CIs) by separating the limits by a hyphen, e.g., xxx - xxx.

Tables and listings are programmed with the following specifications:

File format	Word 2010 document (docx) and/or bookmarked PDF
Page Orientation	Landscape
Font	Titles: Helvetica/Arial 10pt (Table number title bolded)<br><br>Report body and footnotes: Helvetica/Arial 9pt

DocuSign Envelope ID: 09C751E8-FA45-44F5-939E-B2F82C69093A

File format	Word 2010 document (docx) and/or bookmarked PDF	
Margins	Top/Left/Right: 0.875 in.	Bottom: 0.5 in.

Graphic outputs will be produced in PDF format.

6.2. Handling of repeat and unscheduled assessments

Unscheduled assessments will be assigned to the associated visit and timepoint based on date and time of assessment. The assessment closest to the scheduled visit will be used for analysis. For categorical data, the worst value will be used.

7. Analysis Populations

- Intent to Treat (ITT): Subjects who are randomized to one of the formula feeding groups.

- Per Protocol (PP): A subset of the ITT population. It will consist of all subjects who complete the feeding protocol without major protocol violations and who consume a single non-study formula feeding no more than 10 times during the duration of 16-week study as documented on the Other Than Formula Feeding Log. Additionally, subjects who consume more than 3 complete days of non-study formula, defined as greater than 50% of the number of feedings in a 24-hour period from non-study formula, will also be excluded from the PP population. Inclusion criteria states that four anthropometric Z-scores (weight for age, length for age, head circumference for age, and weight for length) had to be $\geq 5^{th}$ and $\leq 95^{th}$ percentile. If two or more of the four anthropometric Z-score inclusion criteria are not met, it will be considered a major protocol violation and the infant will be excluded from the Per Protocol population.

  Given that some sites rely on paper growth charts (hand plotting) of anthropometric measures, occasional small errors may occur with subjects just slightly outside the 5th or 95th percentile being enrolled in the study. If only one of the four anthropometric Z-score inclusion criteria are not met, it will be considered a minor protocol violation and the infant will remain in the Per Protocol population.

- Safety Population: The safety population will be comprised of any subjects who are randomized and consumed at least one feeding of the assigned formula.

DocuSign Envelope ID: 09C751E8-FA45-44F5-939E-B2F82C69093A

Classification into ITT or PP populations will be conducted prior to the database lock. All listings will be provided for the ITT population. A separate listing will be included on the set of subjects who are randomized but never intake the randomized formula before discontinuing from the study.

Since this study has a primary aim at demonstrating non-inferiority, the PP population will represent the primary analysis population to evaluate the treatment groups in terms of "efficacy".

All clinical outcomes (primary and secondary) will be subject to analyses using both the PP and ITT population.

8. Subject Characteristics

8.1. Subject Disposition

Information regarding subject disposition will be summarized for all subjects by treatment group. Summaries will include: number of subjects enrolled, number of subjects in each analysis population, number of subjects completing the study, number of subjects who discontinue the study early. For those who discontinue early, the primary reason for discontinuation will be summarized.

8.2. Demographic and Baseline Characteristics

Demographics variables will include: age, sex, ethnicity, race, and maternal smoking history. Age will be calculated by comparing date of birth to date of informed consent.

Baseline characteristics will include: anthropometry (weight, length, head circumference) and feeding history. Feeding history will include if child was ever breastfed, duration child was breastfed for, and infant's birth weight. All other feeding history information will be listed by subject.

Continuous variables will be summarized using descriptive statistics (mean, median, standard deviation, minimum, and maximum). Categorical variables will be summarized using counts and percentages.

8.3. Protocol Deviations

Protocol deviations will be collected throughout the duration of the study. Protocol deviations will be assigned a sponsor-defined category type. In addition, each deviation will be defined as major and minor. A tabular summary of all major deviations will be generated. In addition, a by-subject listing of all protocol deviations (major and minor) will be produced.

DocuSign Envelope ID: 09C751E8-FA45-44F5-939E-B2F82C69093A

9. Efficacy Analysis

The Per Protocol population will be used for the primary analysis. All clinical outcomes (primary and secondary) will be subject to analyses using both the PP and ITT populations.

The primary efficacy variable is the mean daily weight gain (g/d) over a 16-week study period.

Secondary variables for efficacy to be considered include:

- Anthropometric measurements (head circumference gain velocity, length gain velocity) and Z-scores (weight for age, length for age, weight for length for age, head circumference for age)
- Formula intake volume
- Markers of inflammation (tumor necrosis factor-alpha, interleukin 2, 6, and 10)

9.1. Baseline Values

Unless otherwise noted, baseline is defined as the last non-missing value recorded prior to the first feeding of the assigned formula.

9.2. Handling of Dropouts or Missing Data

Subjects who are enrolled in the study but never received an assigned formula will be considered dropouts. Dropouts will not be replaced.

All subjects who are randomized will be evaluated in the ITT population. Those subjects that do not complete the full study period due to protocol adherence or request to be discontinued from the study will be not be replaced.

In general, analyses will be carried out with the data available with no imputation for missing data.

9.3. Multiple Testing Procedure

To control for the study level Type I error rate, a sequential multiple testing and Hochberg procedure will be used when testing the efficacy endpoints. The primary efficacy endpoint, the mean daily weight gain (g/day) will be compared between Formula A and Formula B. If the non-inferiority criteria is met, then we will proceed to test for non-inferiority for the secondary efficacy endpoints starting with the anthropometric measurements and sex-specific z-scores using the Hochberg step-up multiple testing procedure at $\alpha=0.05$.

DocuSign Envelope ID: 09C751E8-FA45-44F5-939E-B2F82C69093A

The anthropometric measurements and sex-specific z-scores listed in section 10.2.1 will be tested for non-inferiority using Hochberg adjusted 95% confidence intervals. According to the Hochberg step-up multiple testing procedure, the anthropometric secondary endpoints will be ordered according to the adjusted confidence interval. In order, the adjusted confidence interval will be compared to the non-inferiority margin defined for each endpoint to determine if it meets the non-inferiority criteria. Once an endpoint meets the non-inferiority margin, all remaining anthropometric endpoints will be determined to meet the non-inferiority margin.

The formula intake volume endpoint will be tested after all anthropometric endpoints, assuming at least one of the anthropometric measurements or sex-specific z-scores meets the non-inferiority criteria. Formula intake volume will be tested for a significant difference between formula groups at the α level remaining from the Hochberg procedure.

9.4. Interim Analysis and Data Monitoring

An interim analysis will be conducted in a blinded manner when 128 infants (50% of the total goal sample size) have enrolled and completed the two-month time point. Weight gain velocity (g/d) for all infants will be reported descriptively (minimum, maximum, mean, median, standard deviation, first and third quartiles) for the following time intervals: enrollment to 1 month, enrollment to 2 months, 1 month to 2 months. The purpose of the interim analysis is to determine whether or not the assumed standard deviation in weight gain velocity (5.6 g/d) based on reference data by Nelson et al., 1989, is appropriate for our study population. No unblinding or inferential statistics will be performed at the interim; as such no alpha spending procedures will be required.

10. Methods of Efficacy Analysis

10.1. Primary Efficacy Analysis

The primary efficacy endpoint, mean daily weight gain (g/d) over the 16-week study period and at each study visit, will be calculated as (weight in grams at each study visit – weight in grams at baseline visit)/number of days since baseline visit. The weight is measured a minimum of two times at each study visit (a third weight is measured if the first two do not meet the threshold specified in the protocol), and the average of the two or three measurements will be used in the preceding formula. If there are less than 2 measurements recorded, then the average weight measurement will be set to missing. Descriptive statistics (mean, standard deviation, median, minimum, and maximum) of the mean daily weight gain will be presented by visit. Mean daily weight gain will be compared between Formula Groups A and B with a mixed-effect model for repeated measures (MMRM). The test for non-inferiority of the primary efficacy endpoint will have the following hypotheses:

DocuSign Envelope ID: 09C751E8-FA45-44F5-939E-B2F82C69093A

H_0: The difference in mean daily weight gain between the formula groups is less than or equal to -3 g/d at Day 120.

H_1: The difference in mean daily weight gain between the formula groups is greater than -3 g/d at Day 120.

Formula groups will be compared using repeated measures model with gender, age, baseline value, formula, visit, site, formula by site interaction, and formula by visit interaction as factors. The covariance matrix will be chosen based on the AIC criteria to choose the covariance matrix that best fits the model. Non-inferiority will be determined if the lower limit of the two-sided 95% CI for the formula difference is greater than -3, assuming a non-inferiority margin of 3 mg/d.

10.2. Secondary Efficacy Analysis

The following secondary endpoints are of interest and will be analyzed in a similar method as the primary efficacy endpoint. Summary statistics will be presented by visit for each endpoint. The following secondary efficacy endpoints will be analyzed:

Anthropometric measurements:

- Mean daily length gain (cm/d) at visit 9 (Day 120)
- Mean daily head circumference (cm/d) at visit 9 (Day 120)

Sex-specific Z-scores:

- Mean weight-for-age z-score at visit 9 (Day 120)
- Mean length-for-age z-score at visit 9 (Day 120)
- Mean weight-for-length z-score for age at visit 9 (Day 120)
- Mean head circumference-for-age z-score at visit 9 (Day 120)

The average daily volume of formula intake will be compared between formula groups at visits 1, 2, 3, 5, 7, and 9.

These secondary endpoints of interest will be analyzed using the multiple testing procedure described in section 9.3.

10.2.1. Anthropometric measurements

The raw anthropometric measures for weight, length, and head circumference will be collected at visits 1, 2, 3, 5, 7, and 9. These measures will be summarized by visit and formula group with descriptive statistics along with change from baseline. The weight, length, and head circumference are each to be measured a minimum of two times at each study visit (a third measurement is recorded if the first two do not meet the threshold specified in the protocol), and the average of the two or three measurements will be used

in the preceding SAS program. If there are less than 2 measurements recorded, then the average measurement will be set to missing.

Summaries of the derived anthropometric measures (weight gain (g/d) velocity, length gain (cm/d) velocity, and head circumference gain (cm/d) velocity) will be presented by visit and formula group. A MMRM analysis will be used to compare measures between formula groups at Day 120, where a non-inferiority margin, m, is 20% of the mean of the commercial brand formula. The test for non-inferiority of the anthropometric measures will have the following hypotheses:

H_0: The difference in the mean anthropometric measurements between the formula groups is less than or equal to -m at Day 120.

H_1: The difference in the mean anthropometric measurements between the formula groups is greater than -m at Day 120.

Formula groups will be compared using a MMRM model with gender, age, baseline value, formula, visit, site, formula by site interaction, and formula by visit interaction as factors. The covariance matrix will be chosen based on the AIC criteria to choose the covariance matrix that best fits the model. Non-inferiority will be determined if the lower limit of the two-sided 95% CI of the difference in least square means is greater than -m.

10.2.2. Sex-specific Z-scores

Infant growth will also be descriptively summarized on the basis of the following sex-specific z-scores based on the 2009 CDC growth charts for infants and children 0 to 2 years of age, which are based on the World Health Organization growth reference study. The following z-scores will be calculated: weight-for age, length-for-age, weight-for-length, and head circumference-for-age z-scores. For each subject, a line listing of all raw measures of weight, length, head circumference, and all z-scores (weight-for-age z-score, length-for-age z-score, weight-for-length z-score, head circumference-for-age, and BMI-for-age z-score) will be provided. Descriptive statistics will be used to summarize the z-score data by formula group for each visit along with change from baseline at visits 1, 2, 3, 5, 7, and 9. A SAS program and data to be used for the z-scores is found at https://www.cdc.gov/nccdphp/dnpao/growthcharts/resources/sas-who.htm.

A MMRM analysis will be used to compare measures between formula groups over time, where a non-inferiority margin, m, is 20% of the mean of the commercial brand formula.. The test for non-inferiority of the anthropometric measures will have the following hypotheses:

H_0: The difference in the mean z-score between the formula groups is less than or equal to -m at Day 120.

DocuSign Envelope ID: 09C751E8-FA45-44F5-939E-B2F82C69093A

H_1: The difference in the mean z-score between the formula groups is greater than -m at Day 120.

Formula groups will be compared using a MMRM model with gender, age, baseline value, formula, visit, site, formula by site interaction, and formula by visit interaction as factors. The covariance matrix will be chosen based on the AIC criteria to choose the covariance matrix that best fits the model. Non-inferiority will be determined if the lower limit of the two-sided 95% CI of the difference in least square means is greater than -m.

The z-scores generated based on World Health Organization growth reference standards will be plotted for each subject over time.

10.2.3. Formula Intake Volume

The volume of formula consumed in ounces is recorded at visits 1, 2, 3, 5, 7, and 9. An initial 3-day Formula/Diet Record will be recorded for 3 days following the first study visit. The remainder of 3-day Formula/Diet Records will be recorded for 3 days prior to each subsequent scheduled study visit. The average daily volume over the three days will be calculated and summarized by formula group and visit using descriptive statistics, including 95% confidence intervals. A two sample t-test will be used to test a difference between formula groups at each visit.

10.3. Exploratory Analysis

The following exploratory endpoints will be analyzed:

Markers of Inflammation

- Mean value for tumor necrosis factor-alpha at visit 9 (Day 120)
- Mean value for interleukin 2 at visit 9 (Day 120)
- Mean value for interleukin 6 at visit 9 (Day 120)
- Mean value for interleukin 10 at visit 9 (Day 120)

10.3.1. Markers of Inflammation

A single blood sample (of at least 0.5 milliliters but not more than 0.8 milliliters) will be collected from each subject via heel stick at the last study visit (visit 9, Day 120) to assess markers of inflammation (tumor necrosis factor-alpha, interleukin 2, 4, 5, 6, 8, 10, 12, 13, and 17; interleukin 2 receptor, interleukin 1 beta, and interferon gamma). Summary statistics for these biomarkers will be summarized separately by formula group and described in terms of age appropriate reference values, when available.

The tumor necrosis factor-alpha, interleukin-2, interleukin-6, and interlekin-10 markers of inflammation will be used to test the following hypotheses:

H_0: The mean values for the marker of inflammation for each formula group is equal.

H_1: The mean values for the marker of inflammation for each formula group is not equal.

Formula groups will be compared using a two sample t-test. No adjustment for alpha will be made for these exploratory endpoints.

11. Pharmacokinetic Analysis

No pharmacokinetic analysis is planned for this study.

12. Safety Analysis

All safety analysis will be based on the Safety Population. Analysis using the Safety population will be based on the formula the infant is given.

12.1. Gastrointestinal characteristics

For stool composition, stool samples will be collected at the last study visit (visit 9, Day 120) to assess total soap fatty acid concentrations, soap palmitic acid (calcium palmitate) concentrations, and calcium concentration. Dry weights for each concentration will be summarized by formula group with descriptive statistics and will be used to test the following hypotheses:

H_0: The mean values of stool sample concentrations for each formula group is equal.

H_1: The mean values of stool sample concentrations for each formula group is not equal.

Formula groups will be compared using a two-sample t-test. The soap fatty acids which are above the limit of detection will be summarized individually with descriptive statistics by formula group.

Number of bowel movements in the past 24 hours, stool consistency in the past 24 hours, fussiness in the past 24 hours, and amount of gas in the past 24 hours will be assessed and reported separately at each visit. The number of bowel movements in the past 24 hours will be collected and summarized by formula group and visit with descriptive statistics. The responses to the consistency of the bowel movement will be: 0=Watery, 1=Runny-Soft, 2=Mushy-Soft, 3=Formed-Soft, 4=Hard. There are two separate questions for fussiness in the past 24 hours. The first will be coded as 0=Not at all fussy, 1=Slightly fussy, 2=Moderately fussy, 3=Very fussy, and 4=Extremely fussy, with the other question coded as 0=Less fussy than normal, 1=About the same level of fussiness as always, and 2=More fussy than normal. Gas over the past 24 hours also has two separate

DocuSign Envelope ID: 09C751E8-FA45-44F5-939E-B2F82C69093A

questions. One will be coded as 0=None at all, 1=Slight amount of gas, 2=Moderate amount of gas, and 3=Excessive amount of gas, with the other question coded as 0=Less gas than normal, 1=About the same amount of gas as normal, and 2=More gas than normal. The score of each item will be summarized by formula group and visit. The test for treatment effect of the number of bowel movements or scores over time will have the following hypotheses:

H_0: The mean value is equal when comparing the formula groups.

H_1: The mean value is not equal when comparing the formula groups.

Formula groups will be compared using a MMRM model with gender, age, baseline value, formula, site, visit, formula by site interaction, and formula by visit interaction as factors. The covariance matrix will be chosen based on the AIC criteria to choose the covariance matrix that best fits the model. The formula by visit interaction factor will be tested first to see if the formula effect is different across time. If this interaction is statistically significant, the formula effect at each timepoint will be tested and summarized. If this interaction is not statistically significant then the interaction term will be dropped from the model and the overall formula difference will be tested and summarized. In addition, the formula effect by visit will be summarized. An alpha level of 0.10 to test the interaction effect and an alpha level of 0.05 to test the formula effect.

The Infant Characteristics Questionnaire (ICQ) was administered at visits 1, 2, 3, 5, 7, and 9. The ICQ (Appendix A) was developed as a short, factor-analytic screening device for difficultness (Bates et al., 1979). The data from the 24-item questionnaire will be summarized into four subscales: Fussy-difficult, Unadaptable, Dull, and Unpredictable. Based on the examination of the factor structure of the items, the authors of the ICQ composed scales using only the discriminating items for use with their normative sample. These scales are composed as follows:

- Fussy/Difficult: Items 1, 5, 6, 13, 22, and 24
- Unadaptable: Items 9, 10, 11, and 20
- Dull: Items 15 (reverse-coded), 16, and 23
- Unpredictable: Items 2, 3, and 4

The sum of the responses for each of the items above comprise the score for each scale. A response for each item is required to calculate a sum score, otherwise the sum score will be set to missing. Higher scores indicate a more difficult temperament. The scores of the four subscales will be summarized separately by formula group with descriptive statistics at each of the 6 visits. A MMRM analysis will be used to compare sub scale values

DocuSign Envelope ID: 09C751E8-FA45-44F5-939E-B2F82C69093A

between formula groups over time. The test for treatment effect of the scores over time will have the following hypotheses:

H_0: The mean sub scale value is equal when comparing the formula groups.

H_1: The mean sub scale value is not equal when comparing the formula groups.

Formula groups will be compared using a MMRM model with gender, age, baseline sub scale value, formula, site, visit, formula by site interaction, and formula by visit interaction as factors. The covariance matrix will be chosen based on the AIC criteria to choose the covariance matrix that best fits the model. The formula by visit interaction factor will be tested first to see if the formula effect is different across time. If this interaction is statistically significant, the formula effect at each timepoint will be tested and summarized. If this interaction is not statistically significant then the interaction term will be dropped from the model and the overall formula difference will be tested and summarized. In addition, the formula effect by visit will be summarized. An alpha level of 0.10 to test the interaction effect and an alpha level of 0.05 to test the formula effect.

The items which are included in the questionnaire but not included in scales above will be summarized individually with descriptive statistics by visit and formula group.

12.2. Adverse Events

Adverse events summaries will only consider Treatment Emergent Adverse Events (TEAEs). TEAEs are defined as those adverse events that occurred after formula intake and those existing adverse events that worsened during the study. If it cannot be determined whether the adverse event is treatment emergent due to an incomplete (partial) onset date, the adverse event will be considered treatment emergent. Verbatim terms entered into the clinical database via the EDC system will be mapped to preferred terms and system organ classes using the Medical Dictionary for Regulatory Activities (MedDRA), version 20.0.

Each adverse event summary will be displayed by formula. Summaries that are displayed by system organ class and preferred terms will be ordered by descending order of incidence of system organ class and preferred term within each system organ class. Summaries of the following types will be presented:

- Overall summary of the TEAEs which contain an overview of each item below.
- Subject count and incidence rate of TEAEs by MedDRA system organ class (SOC) and preferred term (PT).
- Subject count and incidence rate of TEAEs by MedDRA SOC, PT and highest severity. At each level of subject summarization, a subject is classified according to the highest severity if the subject reported one or more events.

DocuSign Envelope ID: 09C751E8-FA45-44F5-939E-B2F82C69093A

- Subject count and incidence rate of TEAEs by MedDRA SOC, PT and closest relationship to study drug (Related/Not Related). Related AEs are those reported as "Definitely", "Probable" or "Possibly". At each level of subject summarization, a subject is classified according to the closest relationship if the subject reported one or more events. Adverse events with missing relationship will be considered related for this summary.
- Subject count and incidence rate of Serious TEAEs by MedDRA SOC and PT.
- Subject count and incidence rate of TEAEs leading to study discontinuation by MedDRA SOC and PT.

12.2.1. Adverse Events of Special Interest

An overall summary of number and percentage of infants with any physician-reported adverse events of special interest (AEOSI) will be presented by formula group.

Subject count and incidence rate by highest severity and by causality will be presented for all physician-reported AEOSIs, A by-patient listing of all AEOSIs will be provided.

Standardized definitions for common Adverse Events that may or may not be related to formula tolerance (stooling, spit-up, crying, skin issues) were provided to all investigators. Comparisons between formula groups of the incidence of these AEOSIs will be tested using Fisher's Exact tests.

Time to first gastrointestinal event will be analyzed using Kaplan-Meier method by formula group. Kaplan-Meier estimates of the 25th, 50th, and 75th percentiles and the 95% confidence intervals of the median will be provided for each formula group.

ADVERSE EVENT	DEFINITION
**Stooling Issues**	
Difficulty having bowel movement	Crying, fussing, or turning red when having a bowel movement
Hard stools	Healthcare professional diagnosis; pellet or hard rock-like stools
Constipation	Less than 3 bowel movements in 7 days
Acute diarrhea	Runny or watery stools for less than 2 weeks
Chronic diarrhea	Runny or watery stools for more than 2 weeks or ≥3 separate episodes of acute diarrhea in 2 weeks
**Spit-up, Vomiting, GERD issues**	
Regurgitation	Milk comes up into mouth but never out of mouth AND infant DOES NOT arch his/her back as if in pain, stop drinking even if hungry, or cry, wheeze, or cough related to feedings

DocuSign Envelope ID: 09C751E8-FA45-44F5-939E-B2F82C69093A

Infantile spit up	Milk comes out of the mouth after feeding (typically non-forceful), and the amount that comes out is less than half of the feeding volume; non-projectile
Vomiting	Milk comes out of the mouth after feeding (typically forceful), and the amount that comes out is more than half of the feeding volume
Gastroesophageal Reflux Disease (GERD)	Baby arch his/her back as if in pain, stop drinking even if hungry, or cry, wheeze or cough related to feedings (with or without milk coming up into mouth)
**Crying issues**	
Crying/Neonatal abnormal crying	Infant cries for 3 or more hours per day
Infantile colic/Infant colic	Infant cries inconsolably for 3 or more hours per day, at least 3 days per week, AND for at least 3 weeks
**Skin issues**	
Diaper rash	Contact/irritant dermatitis in the diaper area, with erythema and/or skin breakdown on the exposed convex skin surface (rate as Mild, Moderate, Severe using definitions outlined below)<br>**Mild**: Baby has an area of pinkness in the diaper area

12.3. Physical Examination

Physical examinations results will be displayed in by subject listings. No summary tables of physical examination are planned.

12.4. Prior and Concomitant Medications

At each study visit, site personnel will interview parent(s)/guardian(s) to obtain information about all concomitant therapy that was administered since the previous study visit. Concomitant medications include prescription medications, over-the-counter medications, and herbal supplements. This information will be recorded in the infant's medical record.

Any medications or therapy administered and discontinued before the first feeding of randomized formula will be considered as prior medications. Concomitant medications (medications present while consuming randomized formula during the study period) will be recorded throughout the study and at early discontinuation. Prior and concomitant medication verbatim terms captured via the EDC system will be mapped to Anatomical/Therapeutic/Chemical (ATC) class and Preferred Terms using the World Health Organization (WHO) Drug Dictionary, December 2016 version.

DocuSign Envelope ID: 09C751E8-FA45-44F5-939E-B2F82C69093A

Concomitant medications will be summarized for each formula group by WHO ATC class and preferred term. These summaries will present the number and percent of infants using each medication. Subjects may have more than one medication per ATC class and preferred name. At each level of subject summarization, a subject is counted once if one or more medications at that level is reported for the subject. Each summary will be ordered by descending order of incidence of ATC class and preferred term.

Prior medications will be listed by subject.

12.5. Planned Tables, Figures, and Listings

12.5.1. Planned Tables and Figures

Number	Title
14.1.1	Subject Disposition (All Subjects)
14.1.2	Demographics and Baseline Characteristics (Safety Population)
14.1.3	Concomitant Medications (Safety Population)
14.1.4	Medical History (Safety Population)
14.2.1.1.1	Summary of Mean Daily Weight Gain (g/day) by Visit – Repeated Measures Analysis at Visit 9 (Day 120) (Per-Protocol Population)
14.2.1.1.2	Summary of Mean Daily Weight Gain (g/day) by Visit – Repeated Measures Analysis at Visit 9 (Day 120) (ITT Population)
14.2.1.2.1	Summary of Mean Daily Length Gain (cm/day) by Visit – Repeated Measures Analysis at Visit 9 (Day 120) (Per-Protocol Population)
14.2.1.2.2	Summary of Mean Daily Length Gain (cm/day) by Visit – Repeated Measures Analysis at Visit 9 (Day 120) (ITT Population)
14.2.1.3.1	Summary of Mean Daily Head Circumference Gain (cm/day) by Visit – Repeated Measures Analysis at Visit 9 (Day 120) (Per-Protocol Population)
14.2.1.3.2	Summary of Mean Daily Head Circumference Gain (cm/day) by Visit – Repeated Measures Analysis at Visit 9 (Day 120) (ITT Population)
14.2.1.4.1	Summary of Anthropometric Measurements – Actual Values and Change from Baseline by Visit (Per-Protocol Population)
14.2.1.4.2	Summary of Anthropometric Measurements – Actual Values and Change from Baseline by Visit (ITT Population)
14.2.1.5.1	Summary of Sex-Specific Weight-for-Age Z-score by Visit – Repeated Measures Analysis at Visit 9 (Day 120) (Per-Protocol Population)
14.2.1.5.2	Summary of Sex-Specific Weight-for-Age Z-score by Visit – Repeated Measures Analysis at Visit 9 (Day 120) (ITT Population)
14.2.1.6.1	Summary of Sex-Specific Length-for-Age Z-score by Visit – Repeated Measures Analysis at Visit 9 (Day 120) (Per-Protocol Population)
14.2.1.6.2	Summary of Sex-Specific Length-for-Age Z-score by Visit – Repeated Measures Analysis at Visit 9 (Day 120) (ITT Population)
14.2.1.7.1	Summary of Sex-Specific Weight-for-Length-for-Age Z-score by Visit – Repeated Measures Analysis at Visit 9 (Day 120) (Per-Protocol Population)
14.2.1.7.2	Summary of Sex-Specific Weight-for-Length-for-Age Z-score by Visit – Repeated Measures Analysis at Visit 9 (Day 120) (ITT Population)
14.2.1.8.1	Summary of Sex-Specific Head Circumference-for-Age Z-score by Visit – Repeated Measures Analysis at Visit 9 (Day 120) (Per-Protocol Population)
14.2.1.8.2	Summary of Sex-Specific Head Circumference-for-Age Z-score by Visit – Repeated Measures Analysis at Visit 9 (Day 120) (ITT Population)
14.2.1.9.1	Non-inferiority Test Results for Secondary Efficacy Growth Endpoints (Per-Protocol Population)
14.2.1.9.2	Non-inferiority Test Results for Secondary Efficacy Growth Endpoints (ITT Population)

DocuSign Envelope ID: 09C751E8-FA45-44F5-939E-B2F82C69093A

14.2.1.10	Figures of Sex-Specific Weight-for-Age Z-scores by Subject
14.2.2.1	By-Visit Summary of Formula Intake Volume (Per-Protocol Population)
14.2.2.2	By-Visit Summary of Formula Intake Volume (ITT Population)
14.2.3.1	Summary of Inflammatory Markers (Per-Protocol Population)
14.2.3.2	Summary of Inflammatory Markers (ITT Population)
14.3.1.1	Overall Treatment-Emergent Adverse Event Summary (Safety Population)
14.3.1.2	Treatment-Emergent Adverse Events by System Organ Class and Preferred Term (Safety Population)
14.3.1.3	Treatment-Emergent Adverse Events by System Organ Class, Preferred Term, and Severity (Safety Population)
14.3.1.4	Treatment-Emergent Adverse Events by System Organ Class, Preferred Term, and Relationship to Formula (Safety Population)
14.3.1.5	Serious Treatment-Emergent Adverse Events by System Organ Class and Preferred Term (Safety Population)
14.3.1.6	Treatment-Emergent Adverse Events by System Organ Class and Preferred Term Leading to Study Discontinuation (Safety Population)
14.3.2.1	Treatment-Emergent Adverse Events of Special Interest: Stooling, Spit-up, Vomiting, GERD, crying, and skin issues (Safety Population)
14.3.2.2	Treatment-Emergent Adverse Events of Special Interest: Stooling, Spit-up, Vomiting, GERD, crying, and skin issues by Severity (Safety Population)
14.3.2.3	Treatment-Emergent Adverse Events of Special Interest: Stooling, Spit-up, Vomiting, GERD, crying, and skin issues by Relationship to Study Formula (Safety Population)
14.3.2.4	Kaplan-Meier Estimate of Median Time to First Gastrointestinal Event (Safety Population)
14.3.3.1	Summary of Stool Collection Results (Safety Population)
14.3.3.2	Summary of Number of Bowel Movements Per Day by Visit - Repeated Measures Model (Safety Population)
14.3.3.3	Summary of Bowel Movement Consistency by Visit - Repeated Measures Model (Safety Population)
14.3.3.4	Summary of Fussiness by Visit - Repeated Measures Model (Safety Population)
14.3.3.5	Summary of Fussiness Compared to Normal by Visit - Repeated Measures Model (Safety Population)
14.3.3.6	Summary of Gas by Visit - Repeated Measures Model (Safety Population)
14.3.3.7	Summary of Gas Compared to Normal by Visit - Repeated Measures Model (Safety Population)
14.3.3.8	Summary of Fussy/Difficult Subscale of the Infant Characteristics Questionnaire by Visit - Repeated Measures Model (Safety Population)
14.3.3.9	Summary of Unadaptable Subscale of the Infant Characteristics Questionnaire by Visit - Repeated Measures Model (Safety Population)
14.3.3.10	Summary of Dull Subscale of the Infant Characteristics Questionnaire by Visit - Repeated Measures Model (Safety Population)
14.3.3.11	Summary of Unpredictable Subscale of the Infant Characteristics Questionnaire by Visit - Repeated Measures Model (Safety Population)

DocuSign Envelope ID: 09C751E8-FA45-44F5-939E-B2F82C69093A

14.3.3.12	Summary of Questions Not Included in Subscales of the Infant Characteristics Questionnaire (Safety Population)

12.5.2. Planned Listings

Number	Title
16.2.1	Subject Disposition
16.2.2	Protocol Deviations
16.2.3	Inclusion/Exclusion Criteria: Excluded Subjects Only
16.2.4.1.1	Demographics
16.2.4.1.2	Maternal Smoking History
16.2.4.2	Medical History
16.2.4.3	Prior and Concomitant Medications
16.2.4.4	Feeding History
16.2.5.1	Anthropometry Results
16.2.5.2	Stool Characteristics
16.2.5.3	Blood Collection Results
16.2.5.4	Stool Collection Results
16.2.5.5	PRO Formula Intake
16.2.7.1	Adverse Events
16.2.7.2	Serious Adverse Events
16.2.7.3	Treatment-Emergent Adverse Events of Special Interest: Stooling, Spit-up, Vomiting, GERD, Crying, and Skin Issues
16.2.7.4	Treatment-Emergent Adverse Events Leading to Formula Discontinuation or Death
16.2.8.1	Infant Characteristics Questionnaire Results
16.2.8.2	Physical Examinations
16.2.8.3	PRO Daily Feeding Count
16.2.8.4	PRO Other Feeding Log

DocuSign Envelope ID: 09C751E8-FA45-44F5-939E-B2F82C69093A

13. References

Bates JE, Freeland CA, Lounsbury ML. Measurement of infant difficultness. Child development. 1979 Sep 1:794-803.

Nelson SE, Rogers RR, Ziegler EE, Fomon SJ: Gain in weight and length during early infancy. Early Human Development. 1989; 19 (4):223-239.)

DocuSign Envelope ID: 09C751E8-FA45-44F5-939E-B2F82C69093A

Appendix A: Infant Characteristics Questionnaire

On the following questions, please circle the number that is most typical of your baby. "About average" means how you think the typical baby would be scored. Put NA next to any item that does not apply to your baby. For example, if your baby has not had solid food yet, you would mark the item regarding his/her reaction to solid food as NA.

1. How easy or difficult is it for you to calm or soothe your baby when he/she is upset?

1	2	3	4	5	6	7
Very easy			About average			Difficult

2. How easy or difficult is it for you to predict when your baby will go to sleep and wake up?

1	2	3	4	5	6	7
Very easy			About average			Difficult

3. How easy or difficult is it for you to predict when your baby will become hungry?

1	2	3	4	5	6	7
Very easy			About average			Difficult

4. How easy or difficult is it for you to know what's bothering your baby when he/she cries or fusses?

1	2	3	4	5	6	7
Very easy			About average			Difficult

5. How many times per day, on the average, does your baby get fussy and irritable--for either short or long periods of time?

1	2	3	4	5	6	7
Never	1-2 times per day	3-4 times per day	5-6 times per day	7-9 times per day	10-14 times per day	More than 15

6. How much does your baby cry and fuss in general?

1	2	3	4	5	6	7

Very little; much less than the average baby	Average amount; about as much as the average baby	A lot; much more than the average baby

7. How did your baby respond to his/her first bath?

1	2	3	4	5	6	7
Very well-- baby loved it			Neither liked nor disliked it			Terribly-- didn't like it

8. How did your baby respond to his/her first solid food?

1	2	3	4	5	6	7
Very favorably-- liked it immediately			Neither liked nor disliked it			Very negatively--did not like it at all

9. How does your baby typically respond to a new person?

1	2	3	4	5	6	7
Almost always responds favorably			Responds favorably about half the time			Almost always responds negatively at first

10. How does your baby typically respond to being in a new place?

1	2	3	4	5	6	7
Almost always responds favorably			Responds favorably about half the time			Almost always responds negatively at first

11. How well does your baby adapt to things (such as in items 7-10) eventually?

1	2	3	4	5	6	7
Very well, always likes it eventually			Ends up liking it about half the time			Almost always dislikes it in the end

12. How easily does your infant get upset?

1	2	3	4	5	6	7
Very hard to upset-- even by things that upset most babies			About average			Very easily upset by things that wouldn't bother most babies

13. When your baby gets upset (e.g., before feeding, during diapering, etc.), how vigorously or loudly does he/she cry and fuss?

1	2	3	4	5	6	7
Very mild intensity or loudness			Moderate intensity or loudness			Very loud or intense, really cuts loose

DocuSign Envelope ID: 09C751E8-FA45-44F5-939E-B2F82C69093A

14. How does your baby react when you are dressing him/her?

1	2	3	4	5	6	7
Very well-- likes it			About average--doesn't mind it			Doesn't like it at all

15. How active is your baby in general?

1	2	3	4	5	6	7
Very calm and quiet			Average			Very active and vigorous

16. How much does your baby smile and make happy sounds?

1	2	3	4	5	6	7
A great deal, much more than most infants			An average amount			Very little, much less than most infants

17. What kind of mood is your baby generally in?

1	2	3	4	5	6	7
Very happy and cheerful			Neither serious nor cheerful			Serious

18. How much does your baby enjoy playing little games with you?

1	2	3	4	5	6	7
A great deal, really loves it			About average			Very little, doesn't like it very much

19. How much does your baby want to be held?

1	2	3	4	5	6	7
Wants to be free most of the time			Sometimes wants to be held; sometimes not			A great deal-- wants to be held almost all of the time

20. How does your baby respond to disruptions and changes in everyday routine, such as when you go to church or a meeting, on trips, etc.?

1	2	3	4	5	6	7
Very favorably, doesn't get upset			About average			Very unfavorably, gets quite upset

DocuSign Envelope ID: 09C751E8-FA45-44F5-939E-B2F82C69093A

21. How easy is it for you to predict when your baby will need a diaper change?

1	2	3	4	5	6	7
Very easy			About average			Very difficult

22. How changeable is your baby's mood?

1	2	3	4	5	6	7
Changes seldom, and changes slowly when he/she does change			About average			Changes often and rapidly

23. How excited does your baby become when people play with or talk to him/her?

1	2	3	4	5	6	7
Very excited			About average			Not at all

24. Please rate the overall degree of difficulty your baby would present for the average mother.

1	2	3	4	5	6	7
Super easy			Ordinary, some problems			Highly difficult to deal with

25(A). On the average, how much attention does your baby require, other than for caregiving (feeding, diaper changes, etc.)?

1	2	3	4	5	6	7
Very little--much less than average			Average amount			A lot--much more than the average baby

26(B). When left alone, your baby plays well by him/herself.

1	2	3	4	5	6	7
Almost always			About half the time			Almost never--won't play by self

27(C). How does your baby react to being confined (as in a carseat, infant seat, playpen, etc.)?

1	2	3	4	5	6	7
Very well--likes it			Minds a little or protests once in awhile			Doesn't like it at all

DocuSign Envelope ID: 09C751E8-FA45-44F5-939E-B2F82C69093A

28(D). How much does your baby cuddle and snuggle when held?

1	2	3	4	5	6	7
A great deal-- almost every time			Average; sometimes does and sometimes doesn't			Very little; seldom cuddles

Statistical Analysis Plan

Protocol Title:	**Growth and Safety Study of an Infant Formula for Healthy Term Infants**
Protocol Numbers:	**BBN-IF-001**
Version:	4.0
Date:	06 December 2021
Sponsor:	Building Block Nutritionals, LLC<br>200 Garrett St # S<br>Charlottesville, VA 22902
Authors	Ryan McBride<br>Instat Services

This study will be conducted in compliance with the protocol, Good Clinical Practice and all other applicable regulatory requirements, including the archiving of essential documents.

Document History

Versio Number	Author	Date	Change
V1	Ryan McBride	4 Mar 2020	
V2	Ryan McBride	13 Feb 2021	Added information for Phase II of the study.
V3	Ryan McBride	16 Nov 2021	Added information for Analysis Visit Windows. Changed the imputation method to multiple imputation for missing data. Added section on assessment of enrollment time periods for efficacy primary endpoint. Added additional markers of inflammation to analysis. Moisture content added to stool composition analysis.
V4	Ryan McBride	06 Dec 2021	Removed the summary table of abnormal values for inflammation markers. Laboratory values that are below the lower limit of detectability will be set to that lower limit value. Average infant formula intake calculations will not include days with less than 4 records.

Signatures / Approvals

	James McGrath	Dec 7, 2021
EVP, BBN Nutritionals, LLC	Name (Print or Type)	Date

	Ryan McBride	Dec 6, 2021
Senior Biostatistician, Instat Services	Name (Print or Type)	Date

Table of Contents

Signatures / Approvals 2

- List of Abbreviations 5

1. Introduction 7
2. Study Rationale and Objectives 7
   - 2.1. Study Rationale 7
   - 2.2. Study Objectives 8
     - 2.2.1. Primary Efficacy Objective 8
     - 2.2.2. Secondary Efficacy Objective 8
     - 2.2.3. Exploratory Objective 8
     - 2.2.4. Primary Safety Objective 8
     - 2.2.5. Secondary Safety Objective 8
3. Study Design 8
4. FLOWCHART 10
   - 4.1. Study Flowchart 10
5. Determination of Sample Size 11
6. Statistical Methods 12
   - 6.1. Planned Analysis 13
   - 6.2. Reporting conventions 13
   - 6.3. Baseline Values 13
   - 6.4. Handling of Dropouts or Missing Data 13
     - 6.4.1. Coronavirus Disease 19 13
   - 6.5. Handling of repeat and unscheduled assessments 14
   - 6.6. Interim Analysis and Data Monitoring 14
   - 6.7. Multiple Testing Procedure 14
   - 6.8. Adjustments for Covariates 14
7. Analysis Populations 15
8. Subject Characteristics 16
   - 8.1. Subject Disposition 16

8.2. Demographic and Baseline Characteristics 16
8.3. Protocol Deviations 16
9. Methods of Efficacy Analysis 16
9.1. Primary Efficacy Analysis 18
9.1.1. Sensitivity Analyses of the Primary Endpoint 18
9.1.2. Assessment of Enrollment Time Periods 19
9.2. Secondary Efficacy Analysis 20
9.2.1. Growth velocities 20
9.2.2. Growth Z-scores 21
9.2.3. Growth measurements 22
9.2.4. Formula Intake Volume 23
9.3. Exploratory Analysis 23
9.3.1. Markers of Inflammation 24
10. Pharmacokinetic Analysis 24
11. Safety Analysis 24
11.1. Adverse Events 25
11.1.1. Adverse Events of Special Interest 26
11.2. Gastrointestinal Tolerance 28
11.2.1. Stool composition 29
11.2.2. Number of stools 29
11.2.3. Stool consistency 29
11.2.4. Fussiness 29
11.2.5. Amount of gas 29
11.2.6. Infant Characteristics Questionnaire (ICQ) 30
11.3. Physical Examination 31
11.4. Prior and Concomitant Medications 31
12. Planned Tables, Figures, and Listings 32
13. References 33
● Appendix A: Infant Characteristics Questionnaire 34

- **List of Abbreviations**

Abbreviation	Term
ADY	Analysis Relative Day
AE	Adverse Event
AIC	Akaike Information Criterion
ARA	Arachidonic acid
ATC Class	Anatomical/Therapeutic/Chemical Class
BBN	Building Block Nutritionals, LLC
BBN-001 w/ OPN	BBN's infant formula with osteopontin
BBN-102 w/o OPN	BBN's infant formula without osteopontin (test formula)
BMI	Body Mass Index (kg/m^2)
Brand Formula	Commercially available infant formula (control formula)
CDC	Centers for Disease Control and Prevention
CI	Confidence Interval
COVID-19	Coronavirus Disease 2019
CSR	Clinical Study Report
DHA	Docosahexaenoic acid
EDC	Electronic Data Capture
EOS	End of Study
FF	Formula fed
GERD	Gastroesophageal Reflux Disease
GI	Gastrointestinal
GOS	Galacto-oligosaccharide
GRAS	Generally recognized as safe
HM	Human milk
ICF	Informed Consent Form
ICQ	Infant Characteristics Questionnaire
IP	Investigational product
IRB	Institutional Review Board
ITT	Intent to Treat (population)
MedDRA	Medical Dictionary for Regulatory Activities
MMRM	Mixed Model, Repeat Measures
OPN	Osteopontin
PP	Per-Protocol (population)
PT	Preferred Term
RTF	Rich Text Format

SAS	SAS® Analytic Software
SERT	Safety Evaluation of Response Treatment
SOC	System Organ Class
TEAE	Treatment Emergent Adverse Event
TFL	Tables, Figures, Listings
WHO	World Health Organization

1. Introduction

Building Blocks Nutritionals (BBN), LLC has developed an infant formula for term infants that more closely resembles the composition of human milk than other commercially available infant formula products. The purpose of this randomized, controlled, double-blind study is to demonstrate that the BBN formulation meets nutritional requirements and supports age-appropriate growth of healthy term infants. The study background, design and subject assessments are described in the study protocol, BBN-IF-001.

The statistical methods to be implemented during the analyses of data collected within the scope of this study will be outlined in this document. The purpose of this plan is to provide specific guidelines from which the statistical analysis will proceed. Any deviations from this plan will be documented in the clinical study report.

2. Study Rationale and Objectives

2.1. Study Rationale

Human milk (HM) is universally considered the gold standard for infant feeding. HM is a dynamic, multi-faceted fluid containing nutrients and bioactive factors needed for infant health and development. If an infant cannot be breastfed, the American Academy of Pediatrics recommends infant formula as the next best feeding alternative. A goal of infant formula development is to mimic HM in both nutrient composition as well as physiologic outcomes. For years, infant formulas have not been able to provide many of the bioactive factors found in HM. With a goal of providing infants who receive infant formula a nutritional product closer in composition to HM, improvements in infant formula composition are warranted. This study is designed to evaluate the ability of BBN-001 with osteopontin (OPN) and BBN-102 without OPN to support age-appropriate growth in healthy term infants.

Analyses of BBN-001 w/ OPN compared to the Brand Formula and BBN-102 w/o OPN compared to the Brand Formula will occur separately depending on finalization of Generally Recognized as Safe (GRAS) approval of specific ingredients in BBN-001 w/ OPN Formula. This statistical analysis plan will only compare the efficacy and safety of BBN-102 w/o OPN with the Brand Formula.

2.2. Study Objectives

2.2.1. Primary Efficacy Objective

The primary efficacy objective is to compare the growth of infants randomized to the experimental infant formula for term infants (BBN-102 w/o OPN) versus growth of infants randomized to a commercially available term infant formula (Brand Formula).

2.2.2. Secondary Efficacy Objective

The secondary efficacy objective is to compare the formula intake volume between the formula groups (BBN-102 w/o OPN vs Brand Formula).

2.2.3. Exploratory Objective

The exploratory objective is to compare the markers of inflammation (tumor necrosis factor-alpha, interleukin 2, 4, 5, 6, 8, 10, 12, 13 & 17, interleukin 2 receptor, interleukin 1 beta, and interferon gamma) between the formula groups (BBN-102 w/o OPN vs Brand Formula).

2.2.4. Primary Safety Objective

The primary safety objective is to compare the frequency of adverse events (AEs) between the formula groups (BBN-102 w/o OPN vs Brand Formula).

2.2.5. Secondary Safety Objective

The secondary safety objective of this study is to compare the gastrointestinal tolerance (stool composition, bowel movements, stool consistency, gas, fussiness, and ICQ scales) between the formula groups (BBN-102 w/o OPN vs Brand Formula).

3. Study Design

This study is a randomized, controlled, double-blind, study of healthy term formula fed (FF) infants. In the first phase of the study, FF infants will be randomized to receive either a new infant formula formulated for healthy term infants (BBN-001 w/ OPN) or a commercially available infant formula for healthy term infants (Brand Formula). After completion of the first phase, the second phase of the study will randomize infants to receive either BBN-102 w/o OPN or Brand Formula. Infants will consume the formula for a total of 16-weeks; infant growth, serum markers of inflammation, and tolerance to the formulas will be assessed throughout the four-month study period.

The study duration from Day 1 through the EOS visit is approximately 120 days. Each infant will be screened for all inclusion and exclusion criteria. If an infant complies with all inclusion and exclusion criteria and the parent(s)/guardian(s) sign the IRB-approved

Informed Consent Form (ICF), the infant will be randomized to receive a study formula and assigned a unique subject number. If the infant does not comply with one or more of the inclusion or exclusion criteria, the infant will be defined as a screen failure.

Nine visits are scheduled at 15-day intervals with a 3-day visit window.

A flowchart of the study visits is included on the following page.

4. FLOWCHART

Shaded columns indicate visit occurs at study site

4.1. Study Flowchart

Study Visit	1	2	3	4	5	6	7	8	9
Study Day	0	15 ±3	30 ±3	45 ±3	60 ±3	75 ±3	90 ±3	105 ±3	120 ±3
Informed consent	X								
Inclusion/Exclusion criteria	X								
Demography	X								
Randomization	X								
Infant feeding history	X								
Physical Exam	X								
Medical History	X	X	X		X		X		X
Anthropometry[1]	X	X	X		X		X		X
Stool Characteristics and Tolerance Questionnaire	X	X	X	X	X	X	X	X	X
Infant Characteristics Questionnaire (ICQ)	X	X	X		X		X		X
3-day Formula/Diet Record[2]	X	X	X		X		X		X
Blood Collection[3]									X
Dispense stool collection kit[4]							X		
Stool Collection[4]									X
Telephone contact[5]	X			X		X		X	
Concomitant Medications	X	X	X	X	X	X	X	X	X
Adverse events	X	X	X	X	X	X	X	X	X
Dispense and collect study formula	X	X	X		X		X		X

[1]Anthropometry includes assessment of weight, length, and head circumference (see Appendix C in protocol for procedure details).

[2]Initial 3-day Formula/Diet Record will be recorded for 3 days following the first study visit. Remainder of 3-day Formula/Diet Records will be recorded for 3 days prior to each subsequent scheduled study visit.

[3]Blood collection to assess markers of inflammation (tumor necrosis factor-alpha, interleukin 2, 4, 5, 6, 8, 10, 12, 13, & 17; interleukin 2 receptor, interleukin 1 beta, and interferon gamma) and albumin.

[4]Stool collection to assess stool composition. Stool collection supplies will be distributed to parents at Visit 7 for collection of all stools in the 3 days just before Visit 9. The stool collection kit is returned at Visit 9.

[5]Initial telephone contact will occur 3 days after enrollment to check compliance with study feeding and inquire about subject well-being. Remainder of telephone contacts will occur mid-way between clinic visits. Telephone contact notes will be recorded in the study subject's medical record.

5. Determination of Sample Size

Approximately 352 healthy term infants will be enrolled in both phases of the study to complete a minimum of 264 evaluable infants.

Formula fed infants will be randomly allocated to one of the three study formulas (BBN-001 w/ OPN, BBN-102 w/o OPN, Brand Formula) via IWRS (Interactive Web-Based Randomization System). Randomization will be blocked by formula group and stratified by infant sex to allow for balance in the number of males and females in each formula group.

Sample size estimation is based on a test for non-inferiority of the primary efficacy endpoint, mean daily weight gain (g/day), over a 16-week study period. Mean daily weight gain will be compared between formula groups by the calculation of the 95% confidence interval on the difference between the two means. A non-inferiority margin of -3 g/day is set for the difference in mean daily weight gain for the formula-fed groups. Non-inferiority is established, at the one-sided alpha=0.025 significance level, if the lower limit of the two-sided 95% confidence interval on the difference between the two least square means (BBN – Brand) is above -3 g/day.

For phase one of the study, sample size estimation is based on a test for non-inferiority between BBN-001 w/OPN Formula and Brand Formula. Assuming a standard deviation in mean daily weight gain of 5.6 g/day (Nelson et al., 1989) and 80% power, 90 subjects per group (180 total subjects) will be sufficient to demonstrate non-inferiority (one sided alpha=0.025). Assuming a 25% attrition rate, a total of 256 subjects will be enrolled in the BBN-001 w/ OPN Formula and Brand Formula groups.

For phase two of the study, sample size estimation is based on a test for non-inferiority between BBN-102 w/o OPN Formula and Brand Formula. A blinded interim analysis of 81 subjects who completed 16 weeks of the study was performed in phase one of the study to estimate the standard deviation of mean daily weight gain (g/day). Based on this analysis, it is assumed that the standard deviation in mean daily weight gain is 6.0 g/day. Assuming this standard deviation and approximately 90% power, approximately 168 subjects (64 in the BBN-102 w/o OPN Formula group and 104 in the Brand Formula group) will be sufficient to demonstrate non-inferiority (one-sided alpha=0.025). Assuming a 25% attrition rate, a total of approximately 96 subjects will be enrolled in phase two.

The plan is to use the 129 subjects randomized to Brand Formula in the first phase of the study. Assuming a 25% attrition rate, we estimate 96 subjects will meet the Per Protocol (PP) population criteria. This data will remain blinded through the enrollment of phase

two of the study. In phase two of the study, infants will be randomized to Brand Formula or BBN-102 w/o OPN in a 1:8 ratio. Assuming a 25% attrition rate and to obtain at least 90% power, approximately 10 subjects randomized to Brand Formula and 86 subjects randomized to BBN-102 w/o OPN Formula will be enrolled in phase two of the study. With the addition of the 129 subjects from phase one of the study, there will be approximately a total of 139 subjects enrolled in the Brand Formula group and 86 subjects in the BBN-102 w/o OPN Formula group. Infants will be stratified by sex to achieve balance of males and females within each formula group.

At the conclusion of the study, a post-hoc power analysis on weight gain will be conducted.

6. Statistical Methods

The statistical analyses will be reported using summary tables, figures and listings (TFLs). Numbering for TFLs will be based on the recommended numbering convention provided by the International Conference on Harmonisation. Unless noted otherwise, all statistical tests will be two-sided with a significance level of $\alpha = 0.05$. Tests will be declared statistically significant if the calculated p-value is ≤ 0.05. Continuous variables will be summarized with means, standard deviations, medians, minimums and maximums. Categorical variables will be summarized by counts and percent of subjects in corresponding categories. Missing values are not considered for percent calculations, unless stated otherwise. In those cases, footnotes will specify the percent basis. All summary tables will be presented by formula received. Select baseline tables may also include a total summary.

Individual subject data obtained via the electronic data capture (EDC) system and from external vendors will be presented in by subject listings.

The analyses described in this plan are considered *a priori*, in that they have been defined prior to the blind being broken and the database locked. Any analysis performed after breaking the blind will be considered post-hoc and exploratory. Post-hoc analyses will be labeled as such on the output and identified in the CSR.

All analyses and tabulations will be performed using SAS® version 9.4 or higher. Tables and listings will be presented in pdf and rtf format. Upon completion, all SAS programs will be validated by an independent programmer. The validation process will be used to confirm that statistically valid methods have been implemented and that all data manipulations and calculations are accurate. Checks will be made to ensure accuracy,

consistency with this plan, consistency with tables and consistency between tables and corresponding data listings.

6.1. Planned Analysis

Analysis of primary and secondary endpoints for the randomized BBN-102 w/o OPN Formula and Brand Formula groups will be performed at the end of the 16-week analysis. The 16-week analysis will use all efficacy and safety data collected for the BBN-102 w/o OPN Formula and Brand Formula groups. The analysis of BBN-001 w/ OPN Formula compared to the Brand Formula will occur separately depending on finalization of Generally Recognized as Safe (GRAS) approval of specific ingredients in BBN-001 w/ OPN Formula.

6.2. Reporting conventions

P-values ≥0.0001 will be reported to 4 decimal places; P-values less than 0.0001 will be reported as "<0.0001". The mean will be reported to one decimal place greater than the original data. The standard deviation (SD) will be reported to two decimal places greater than the original data. Quantiles, such as median, Q1, Q3 or minimum (Min) and maximum (Max) will use the same number of decimal places as the original data. Estimated parameters, not on the same scale as raw observations (e.g., regression coefficients) will be reported to 3 significant figures.

6.3. Baseline Values

Unless otherwise noted, baseline is defined as the last non-missing value recorded prior to the first feeding of the assigned formula.

6.4. Handling of Dropouts or Missing Data

Subjects who are randomized in the study but never received an assigned formula will be considered dropouts. Dropouts will not be replaced.

All subjects who are randomized will be evaluated in the ITT population. Those subjects that do not complete the full study period due to protocol adherence or request to be discontinued from the study will not be replaced.

In general, analyses will be carried out with the data available with no imputation for missing data.

6.4.1. Coronavirus Disease 19

Due to the Coronavirus Disease 19 (COVID-19) outbreak during the course of the study, there may be several subjects who will have missing assessments which could affect the primary analysis. Subjects who are unable to attend a study visit due to COVID-19 will

be documented in the protocol deviation log. The protocol deviation date will be used as the COVID-19 affected date (or visit). If a subject reports an adverse event of COVID-19, then the event start date will be used as the affected date. In the event that a subject has an affected COVID-19 date from both sources, the earlier of the dates will be used.

Subjects who were affected by COVID-19 will be summarized in a data listing. The listing will contain the following information: subject number, formula, sex, age, first date affected by COVID-19, duration in study prior to the affected COVID-19 date, total study duration, exposure to study formula, any COVID-19 related adverse events, completion/discontinuation of the study, and reasons for discontinuation. All data listings will include a flag to denote whether the subject was affected by COVID-19.

6.5. Handling of repeat and unscheduled assessments

Unscheduled assessments will be assigned to the associated visit and timepoint based on date and time of assessment. The assessment closest to the scheduled visit will be used for analysis.

6.6. Interim Analysis and Data Monitoring

A blinded interim analysis of 81 subjects who completed 16 weeks of the study was performed in the first phase of the BBN-IF-001 study to estimate the standard deviation of weight gain. Based on this analysis, it is assumed that the standard deviation in weight gain is 6.0 g/day. No formal data monitoring is planned for this study.

6.7. Multiple Testing Procedure

The secondary endpoints (growth velocities, growth z-scores, growth measurements, and formula intake) will be analyzed between Brand Formula and BBN-102 w/o OPN formula groups. Each secondary endpoint will be analyzed using the Hochberg step-up multiple testing procedure to control for the familywise error rate at level α. The tests of statistical difference will be tested using a Hochberg corrected p-value.

6.8. Adjustments for Covariates

Adjustments for sex, age at enrollment, baseline values, formula, visit, pooled site, formula by pooled site interaction, and formula by visit interaction will be made for statistical analyses. If the model does not converge when using all these covariates, then the formula by pooled site interaction term will be dropped from the model.

Some of the sites have very few subjects. All sites in the same state will be grouped together and then states will be grouped together geographically into a new pooled site variable.

7. Analysis Populations

- Intent to Treat (ITT): All randomized subjects in the study based on the formula group they are randomized to.

- Per Protocol (PP): A subset of the ITT population. It will consist of all subjects who complete the feeding protocol without significant protocol violations, have weight measurements at both baseline and Week 16, and who consume a single non-study formula feeding no more than 10 times during the duration of 16-week study as documented on the Other Than Formula Feeding Log. Additionally, subjects who consume more than 3 complete days of non-study formula, defined as greater than 50% (4 feedings) of the number of feedings in a 24-hour period from non-study formula, will also be excluded from the PP population. Inclusion criteria states that four anthropometric Z-scores (weight for age, length for age, head circumference for age, and weight for length) had to be ≥5th and ≤95th percentile. If two or more of the four anthropometric Z-score inclusion criteria are not met, it will be considered a significant protocol violation and the infant will be excluded from the Per Protocol population.

  Given that some sites rely on paper growth charts (hand plotting) of anthropometric measures, occasional small errors may occur with subjects just slightly outside the 5th or 95th percentile being enrolled in the study. If only one of the four anthropometric Z-score inclusion criteria are not met, it will be considered a minor protocol violation and the infant will remain in the Per Protocol population.

- Safety Population: The safety population will be comprised of any subjects who are randomized and consumed at least one feeding of study formula.

Classification into ITT or PP populations will be conducted prior to the database lock. All listings will be provided for the ITT population. A separate listing will be included on the set of subjects who are randomized but never intake the randomized formula before discontinuing from the study.

Since this study has a primary aim at demonstrating non-inferiority, the PP population will represent the primary analysis population to evaluate the treatment groups in terms of “efficacy”.

All clinical outcomes (primary and secondary) will be subject to analyses using both the PP and ITT population.

8. Subject Characteristics

8.1. Subject Disposition

Information regarding subject disposition will be summarized for all subjects by treatment group. Summaries will include: number of subjects enrolled, number of subjects in each analysis population, number of subjects completing the study, number of subjects who discontinue the study early. For those who discontinue early, the primary reason for discontinuation will be summarized.

8.2. Demographic and Baseline Characteristics

Demographics variables will include: age at enrollment, sex, ethnicity, race, and maternal smoking history. Age will be calculated by comparing date of birth to date of informed consent.

Baseline characteristics will include: anthropometry (weight, length, head circumference) and feeding history. Feeding history will include if child was ever breastfed, duration child was breastfed for, and infant's birth weight. All other feeding history information will be listed by subject. Initial visit assessments will also include a physical exam, a review of medical history, maternal smoking history, stool characteristics and tolerance.

Continuous variables will be summarized using descriptive statistics (mean, median, standard deviation, minimum, and maximum). Categorical variables will be summarized using counts and percentages.

8.3. Protocol Deviations

Protocol deviations will be collected throughout the duration of the study. Protocol deviations will be assigned a sponsor-defined category type. In addition, each deviation will be defined as significant or minor. A tabular summary of all significant deviations will be generated. In addition, a by-subject listing of all protocol deviations (significant and minor) will be produced.

9. Methods of Efficacy Analysis

Since the study's primary aim is to demonstrate non-inferiority in mean daily weight gain (g/day) between the formula groups, the Per Protocol Population will represent the primary analysis population to evaluate the formula groups in terms of "efficacy". All clinical outcomes (primary and secondary) will be subject to analyses using both the PP and ITT Populations. Analyses using the PP and ITT Populations will be based on the formula the infant is randomized to.

The primary efficacy variable is the mean daily weight gain (g/day) over a 16-week study period. Secondary variables for efficacy to be considered include:

- Anthropometric measures
- Formula intake volume

Efficacy endpoints will be summarized by study visits at study sites. The protocol schedule of assessment lists target visit days that occur every 15 or 30 days and associated tests to be conducted on these visits and also specify associated visit windows (+/- 3 days) for these visits. However, it is not uncommon for subjects to have visits outside the protocol specified visit windows due to various reasons. Because of this, analysis visits and windows are specified below to include as much data as possible in the analyses. While presenting summaries of data by visit, analysis visits will be used according to the windows specified below. Using the analysis visits and windows allow for more comparable comparisons of test results which are closest to the protocol specified target visits and time on study formula while correcting for possible issues that occurred during the study.

Analysis Visit	Target Day	Window
1	0	N/A
2	15	8 – 22
3	30	23 – 44
5	60	45 – 74
7	90	75 – 104
9	120	105 – 134

Analysis relative day (ADY) will be derived and used to determine the appropriate analysis visit. The subject’s randomization date (ADY = -1) will be used to calculate the treatment start day (ADY =1) and the analysis relative day for each study visit. ADY will be used to compare to the analysis visit target day to identify the analysis visit. If more than one visit falls within a window, then the visit closest to the target day will be used in the analysis (ANL02FL = Y). If more than one visit falls within a window and they are the same distance to the target day, then the latest visit will be used in the analysis. Unscheduled assessments will be assigned to the associated analysis visit based on date and time of assessment.

9.1. Primary Efficacy Analysis

The primary efficacy endpoint, mean daily weight gain (g/day) over the 16-week study period and at each study visit, will be calculated as (average weight in grams at each study visit – average weight in grams at baseline visit)/number of days since baseline visit. The weight is measured a minimum of two times at each study visit (a third weight is measured if the first two do not meet the threshold specified in the protocol). If two measurements are taken, then the average of the two will be used in the preceding formula. If there are more than two measurements, then the two measurements that are the closest will be averaged and used in the preceding formula. If three measurements are equidistant, then the lower two measurements will be averaged and used in the preceding formula. If there are less than 2 measurements recorded, then the average weight measurement will be set to missing. This average weight measurement will be used in the calculation for mean daily weight gain above. Descriptive statistics of the mean daily weight gain will be presented by visit and formula group. Mean daily weight gain at visit 9 (Day 120) will be compared between BBN-102 w/o OPN and the Brand Formula with a mixed-effect model for repeated measures (MMRM). The test for non-inferiority of the primary efficacy endpoint will have the following hypotheses:

H_0: The difference (BBN-102 w/o OPN – Brand Formula) in mean daily weight gain between the formula groups is less than or equal to -3 g/day at visit 9 (Day 120).

H_1: The difference (BBN-102 w/o OPN – Brand Formula) in mean daily weight gain between the formula groups is greater than -3 g/day at visit 9 (Day 120).

Formula groups will be compared using a MMRM with sex, age at enrollment, baseline value, formula, visit, pooled site, formula by pooled site interaction, and formula by visit interaction as factors. The covariance matrix will be chosen based on the Akaike Information Criterion (AIC) to choose the covariance matrix that best fits the model. Non-inferiority will be determined if the lower limit of the two-sided 95% CI for the difference (BBN-102 w/o OPN – Brand Formula) in least square means is greater than -3, assuming a non-inferiority margin of 3 g/day.

9.1.1. Sensitivity Analyses of the Primary Endpoint

The primary efficacy endpoint, mean daily weight gain (g/day), is to be assessed at Treatment Visit 9 (Day 120). Due to missing data, sensitivity analyses will be performed to assess the robustness of the primary analysis. The purpose of these analyses is to assess any potential bias due to subject assessments occurring during COVID-19 or missing data due to early dropouts or missed visits. There will be two sensitivity analyses on the

primary efficacy endpoint, mean daily weight gain (g/day) at Day 120, for both the ITT and Per-Protocol Populations.

- Exclude COVID-19 affected data: The first analysis will be to conduct the planned analysis only on the subjects who were not affected by COVID-19 at or before Day 120 (with visit window), i.e., if a subject was affected by COVID-19, then the subject will be removed from the analysis.
- Multiple imputation of missing data: The second analysis will be to conduct the planned analysis on all subjects and will use the multiple imputation method to support the analysis. The Markov Chain Monte Carlo (MCMC) method will be used to impute the missing data within each formula group under the missing at random paradigm. One hundred imputed datasets will be generated. Each dataset will be analyzed using the analysis model specified above. The final estimate of difference between formula groups and inferences will be handled using PROC MIANALYZE.

9.1.2. Assessment of Enrollment Time Periods

The primary efficacy endpoint, mean daily weight gain (g/day), is to be assessed at Treatment Visit 9 (Day 120). The Brand Formula group in this analysis includes subjects that were enrolled in either phase one or phase two of the study. Due to enrollment of subjects during different time periods, we will assess the assumption that growth for subjects is similar during these different time periods. To do this we will compare the growth of subjects in the Brand Formula group that enrolled in the first phase of the study with those that enrolled in the second phase of the study. Summary statistics with 95% confidence intervals for mean daily weight gain (g/day) for all subjects in the Brand Formula group from phase one of the study and all subjects in the Brand Formula group from phase two of the study will be produced.

To assess if growth is similar between the two enrollment time periods we will generate 100 random samples from subjects in the Brand Formula group from phase one of the study. These random samples will be the same size as the number of subjects in the Brand Formula group from phase two of the study. The mean and standard error of the 100 random samples along with the 95% confidence interval for mean daily weight gain (g/day) for the Brand Formula group from phase one and the summary statistics with 95% confidence intervals for mean daily weight gain (g/day) for the Brand Formula group from phase two of the study will be summarized. This will be done for both the ITT and Per-Protocol Populations.

9.2. Secondary Efficacy Analysis

The following secondary endpoints (growth velocities, growth z-scores, growth measurements, and formula intake) are of interest and will be analyzed in a similar method as the primary efficacy endpoint. Each secondary endpoint will be analyzed using the Hochberg step-up multiple testing procedure to control for the familywise error rate at level α. Statistical difference of formula groups will be tested using Hochberg corrected p-values. The following secondary efficacy endpoints will be analyzed:

Growth velocities:

- Mean daily length gain (mm/day) at visit 9 (Day 120)
- Mean daily head circumference gain (mm/day) at visit 9 (Day 120)

Growth Z-scores:

- Mean weight-for-age z-score at visit 9 (Day 120)
- Mean length-for-age z-score at visit 9 (Day 120)
- Mean weight-for-length z-score for age at visit 9 (Day 120)
- Mean head circumference-for-age z-score at visit 9 (Day 120)

Growth measurements:

- Mean weight (g) at visit 9 (Day 120)
- Mean length (cm) at visit 9 (Day 120)
- Mean head circumference (cm) at visit 9 (Day 120)

The average daily volume of formula intake will be compared between formula groups at visits 1, 2, 3, 5, 7, and 9.

9.2.1. Growth velocities

Mean daily length gain (mm/day) and mean daily head circumference gain (mm/day) over the 16-week study period and at each study visit, will be calculated as (average length or average head circumference in mm at each study visit – average length or average head circumference in mm at baseline visit)/number of days since baseline visit. The length and head circumference are measured a minimum of two times at each study visit (a third measurement is taken if the first two do not meet the threshold specified in the protocol). If two measurements are taken, then the average of the two will be used in the preceding formula. If there are more than two measurements, then the two measurements that are the closest will be averaged and used in the preceding formula. If three measurements are equidistant, then the lower two measurements will be averaged and used in the preceding formula. If there are less than 2 measurements recorded, then

the average weight measurement will be set to missing. This average measurement will be used in the calculation for mean daily length and head circumference gain above.

Descriptive statistics of the derived growth velocities (mean daily length gain and mean daily head circumference gain) will be presented by visit and formula group. A MMRM analysis will be used to compare measures between formula groups at visit 9 (Day 120). The test for statistical difference of the growth velocity between formula groups will have the following hypotheses:

H_0: There is no difference in the mean daily growth velocity between the formula groups at visit 9 (Day 120).

H_1: There is a difference in the mean daily growth velocity between the formula groups at visit 9 (Day 120).

Formula groups will be compared using a MMRM with sex, age at enrollment, baseline value, formula, pooled site, visit, formula by pooled site interaction, and formula by visit interaction as factors. The covariance matrix will be chosen based on the AIC criteria to choose the covariance matrix that best fits the model. 95% confidence intervals and p-values will be reported on the difference of least square means for comparison of formula groups.

9.2.2. Growth Z-scores

Infant growth will also be descriptively summarized using z-scores based on the 2009 CDC growth charts for infants and children 0 to 2 years of age, which are based on the World Health Organization growth reference study. The following z-scores will be calculated using the average measurements: weight-for age, length-for-age, weight-for-length, and head circumference-for-age z-scores. For each subject, a line listing of all z-scores (weight-for-age z-score, length-for-age z-score, weight-for-length z-score, head circumference-for-age, and BMI-for-age z-score) will be provided. Descriptive statistics will be used to summarize the z-score data by visit and formula group. A SAS program and data to be used for the z-scores is found at https://www.cdc.gov/nccdphp/dnpao/growthcharts/resources/sas-who.htm.

A MMRM analysis will be used to compare measures between formula groups at visit 9 (Day 120). The test for statistical difference of the Z-scores between formula groups will have the following hypotheses:

H_0: There is no difference in mean z-scores between the formula groups at visit 9 (Day 120).

H_1: There is a difference in mean z-scores between the formula groups at visit 9 (Day 120).

Formula groups will be compared using a MMRM with sex, age at enrollment, baseline value, formula, pooled site, visit, formula by pooled site interaction, and formula by visit interaction as factors. The covariance matrix will be chosen based on the AIC criteria to choose the covariance matrix that best fits the model. 95% confidence intervals and p-values will be reported on the difference of least square means for comparison of formula groups.

The z-scores generated based on World Health Organization growth reference standards will be plotted for each subject over time.

9.2.3. Growth measurements

The growth measurements for weight, length, and head circumference will be collected at visits 1, 2, 3, 5, 7, and 9. The weight, length, and head circumference are each to be measured a minimum of two times at each study visit (a third measurement is taken if the first two do not meet the threshold specified in the protocol). If two measurements are taken, then the average of the two will be used. If there are more than two measurements, then the two measurements that are the closest will be. If three measurements are equidistant, then the lower two measurements will be averaged. If there are less than 2 measurements recorded, then the average weight measurement will be set to missing.

Descriptive statistics of the average growth measurements (weight, length, and head circumference) will be presented by visit and formula group. A MMRM analysis will be used to compare measures between formula groups at visit 9 (Day 120). The test for statistical difference of the growth measurements between formula groups will have the following hypotheses:

H_0: There is no difference in the mean growth measurement between the formula groups at visit 9 (Day 120).

H_1: There is a difference in the mean growth measurement between the formula groups at visit 9 (Day 120).

Formula groups will be compared using a MMRM with sex, age at enrollment, baseline value, formula, pooled site, visit, formula by pooled site interaction, and formula by visit interaction as factors. The covariance matrix will be chosen based on the AIC criteria to

choose the covariance matrix that best fits the model. 95% confidence intervals and p-values will be reported on the difference of least square means for comparison of formula groups.

Growth measures will be plotted for each subject over time.

9.2.4. Formula Intake Volume

The volume of formula consumed in ounces is recorded at visits 1, 2, 3, 5, 7, and 9. An initial 3-day Formula/Diet Record will be recorded for 3 days following the first study visit. The remainder of 3-day Formula/Diet Records will be recorded for 3 days prior to each subsequent scheduled study visit. Records will be assigned to 24-hour windows. The average daily volume (oz/day) will be calculated by averaging the 24-hour windows with sufficient information. 24-hour windows that haver less than 4 feeding records will not be included when calculating the average daily volume. The average daily volume will be summarized by formula group and visit using descriptive statistics. A two-sample t-test will be used to test a difference between formula groups at each visit with 95% confidence intervals and p-values presented.

Average daily formula intake (oz/kg/day) and average daily formula intake (mL/kg/day) will also be summarized by formula group and visit using descriptive statistics.

9.3. Exploratory Analysis

The following exploratory endpoints will be analyzed:

Markers of Inflammation

- Mean value for tumor necrosis factor-alpha at visit 9 (Day 120)
- Mean value for interleukin 2 at visit 9 (Day 120)
- Mean value for interleukin 4 at visit 9 (Day 120)
- Mean value for interleukin 5 at visit 9 (Day 120)
- Mean value for interleukin 6 at visit 9 (Day 120)
- Mean value for interleukin 8 at visit 9 (Day 120)
- Mean value for interleukin 10 at visit 9 (Day 120)
- Mean value for interleukin 12 at visit 9 (Day 120)
- Mean value for interleukin 13 at visit 9 (Day 120)
- Mean value for interleukin 17 at visit 9 (Day 120)
- Mean value for interleukin 2 receptor at visit 9 (Day 120)
- Mean value for interleukin 1 beta at visit 9 (Day 120)
- Mean value for interferon gamma at visit 9 (Day 120)

9.3.1. Markers of Inflammation

A single blood sample (of at least 0.5 milliliters but not more than 0.8 milliliters) will be collected from each subject via heel stick at the last study visit (visit 9, Day 120) to assess markers of inflammation (tumor necrosis factor-alpha, interleukin 2, 4, 5, 6, 8, 10, 12, 13, and 17; interleukin 2 receptor, interleukin 1 beta, and interferon gamma). Biomarker values that are below the lower limit of detectability will be set to that lower limit value (for example, < 5 ng/mL will be replaced with 5 ng/mL). Summary statistics for these biomarkers will be summarized separately by formula group. Formula groups will be compared using a two-sample t-test with 95% confidence intervals and p-values to test the following hypotheses:

H_0: The mean values for the marker of inflammation for each formula group is equal.

H_1: The mean values for the marker of inflammation for each formula group is not equal.

No adjustment for alpha will be made for these exploratory endpoints.

10. Pharmacokinetic Analysis

No pharmacokinetic analysis is planned for this study.

11. Safety Analysis

All safety analysis will be based on the Safety Population. Analysis using the Safety population will be based on the formula the infant is given.

The gastrointestinal tolerance safety endpoints will be summarized by study visits. The protocol schedule of assessment lists target visit days that occur every 15 days and associated tests to be conducted on these visits and also specify associated visit windows (+/- 3 days) for these visits. However, it is not uncommon for subjects to have visits outside the protocol specified visit windows due to various reasons. Because of this, analysis visits and windows are specified below to include as much data as possible in the analyses. While presenting summaries of data by visit, analysis visits will be used according to the windows specified below. Using the analysis visits and windows allow for more comparable comparisons of test results which are closest to the protocol specified target visits and time on study formula while correcting for possible issues that occurred during the study.

Analysis Visit	Target Day	Window

1	0	N/A
2	15	8 – 22
3	30	23 – 37
4	45	38 – 52
5	60	53 – 67
6	75	68 – 82
7	90	83 – 97
8	105	98 – 112
9	120	113 – 134

Analysis relative day (ADY) will be derived and used to determine the appropriate analysis visit. The subject's randomization date (ADY = -1) will be used to calculate the treatment start day (ADY =1) and the analysis relative day for each study visit. ADY will be used to compare to the analysis visit target day to identify the analysis visit. If more than one visit falls within a window, then the visit closest to the target day will be used in the analysis (ANL02FL = Y). If more than one visit falls within a window and they are the same distance to the target day, then the latest visit will be used in the analysis. Unscheduled assessments will be assigned to the associated analysis visit based on date and time of assessment.

11.1. Adverse Events

Adverse events (AE) summaries will only consider Treatment Emergent Adverse Events (TEAEs). TEAEs are defined as those adverse events with date of onset after formula intake and those existing adverse events that worsened during the study. If it cannot be determined whether the AE is treatment emergent due to a missing or incomplete (partial) onset date, the AE will be considered treatment emergent. Verbatim terms entered into the clinical database via the EDC system will be mapped to preferred terms and system organ classes using the Medical Dictionary for Regulatory Activities (MedDRA), version 23.0.

Each AE summary will be displayed by formula group. Summaries that are displayed by system organ class and preferred terms will be ordered by descending order of incidence of system organ class and preferred term within each system organ class. Summaries of the following types will be presented:

- Overall summary of the TEAEs which contain an overview of each item below.
- Subject count and incidence rate of TEAEs by MedDRA system organ class (SOC) and preferred term (PT).

- Subject count and incidence rate of TEAEs by MedDRA SOC, PT and highest severity. At each level of subject summarization, a subject is classified according to the highest severity if the subject reported one or more events.
- Subject count and incidence rate of TEAEs by MedDRA SOC, PT and closest relationship to study drug (Related/Not Related). Related AEs are those reported as "Related" or "Possibly Related". At each level of subject summarization, a subject is classified according to the closest relationship if the subject reported one or more events. Adverse events with missing relationship will be considered related for this summary.
- Subject count and incidence rate of Serious TEAEs by MedDRA SOC and PT.
- Subject count and incidence rate of TEAEs leading to study discontinuation by MedDRA SOC and PT.

11.1.1. Adverse Events of Special Interest

An overall summary of number and percentage of infants with any physician-reported AE of special interest will be presented by formula group.

Subject count and incidence rate by highest severity and by causality will be presented for all physician-reported AEs of special interest, A by-patient listing of all AEs of special interest will be provided.

Standardized definitions for common AEs that may or may not be related to formula tolerance (stooling, spit-up, vomiting, GERD, crying, skin issues**)** were provided to all investigators. Comparisons between formula groups of the incidence of these AEs of special interest will be tested using Fisher's Exact tests.

Time to first gastrointestinal event will be analyzed using Kaplan-Meier method by formula group. Kaplan-Meier estimates of the 25th, 50th, and 75th percentiles and the 95% confidence intervals of the median will be provided for each formula group.

ADVERSE EVENT	DEFINITION
**Stooling Issues**	
Difficulty having bowel movement	Crying, fussing, or turning red when having a bowel Movement
Hard stools	Healthcare professional diagnosis; pellet or hard rock-like<br>Stools
Constipation	Less than 3 bowel movements in 7 days
Acute diarrhea	Runny or watery stools for less than 2 weeks

Chronic diarrhea	Runny or watery stools for more than 2 weeks or ≥3 separate episodes of acute diarrhea in 2 weeks
**Spit-up, Vomiting, GERD issues**	
Regurgitation	Milk comes up into mouth but never out of mouth AND infant DOES NOT arch his/her back as if in pain, stop drinking even if hungry, or cry, wheeze, or cough related to feedings
Infantile spit up	Milk comes out of the mouth after feeding (typically non-forceful), and the amount that comes out is less than half of the feeding volume; non-projectile
Vomiting	Milk comes out of the mouth after feeding (typically forceful), and the amount that comes out is more than half of the feeding volume
Gastroesophageal Reflux Disease (GERD)	Baby arches his/her back as if in pain, stop drinking even if hungry, or cry, wheeze or cough related to feedings (with or without milk coming up into mouth)
**Crying issues**	
Crying/Neonatal abnormal crying	Infant cries for 3 or more hours per day
Infantile colic/Infant colic	Infant cries inconsolably for 3 or more hours per day, at least 3 days per week, AND for at least 3 weeks
**Skin issues**	
Diaper rash	Contact/irritant dermatitis in the diaper area, with erythema and/or skin breakdown on the exposed convex skin surface (rate as Mild, Moderate, Severe using definitions outlined below)<br><br>**Mild**: Baby has an area of pinkness in the diaper area<br>**Moderate**: Baby has definite pinkness in a large area with some small areas of definite redness<br>**Severe**: Baby has intense redness over a large area of the perianal region, or any area of redness with skin breakdown in the diaper area
Atopic dermatitis/Eczema	Any skin lesions, not in the diaper area. Score for any erythema, edema/papulation, and/or excoriation on scale from 0-3 each (9 points max). For each item, 0 = none, 1 = mild, 2 = moderate, 3 = severe.<br><br>**Mild**: 1 or 2 points<br>**Moderate**: 3 to 5 points

	**Severe**: 6 to 9 points

11.2. Gastrointestinal Tolerance

Gastrointestinal tolerance (stool composition, number of stools per day, stool consistency, fussiness, gas, Infant Characteristics Questionnaire (ICQ) scores) will be compared between BBN-102 w/o OPN and Brand Formula. The studentized residuals for each endpoint will be assessed for normality using a histogram and quantile-quantile plot. If the normality assumption holds, then the gastrointestinal characteristic or general disposition score will be summarized by formula group and visit with descriptive statistics. Treatment effect will be compared using a mixed-effect model for repeated measures (MMRM) with sex, age, baseline value, formula, pooled site, visit, formula by pooled site interaction, and formula by visit interaction as factors. The covariance matrix will be chosen based on the AIC criteria to choose the covariance matrix that best fits the model. In addition, the formula effect by visit will be summarized. An alpha level of 0.05 will be used to test the formula effect. 95% confidence intervals and p-values will be reported on the difference of least square means for comparison of formula groups at each visit. The test for treatment effect of the score for the gastrointestinal characteristic or general disposition over the 16-week period will have the following hypotheses:

H_0: There is no difference between the formula groups.

H_1: There is a difference between the formula groups.

If departure from normality is detected, then the gastrointestinal characteristic or general disposition responses will be summarized by frequencies and percentages for each category by formula group and visit. Formula groups will be compared using a Multinomial Logistic Regression model with sex, age at enrollment, baseline value, formula, pooled site, visit, formula by pooled site interaction, and formula by visit interaction as factors. The default SAS covariance structure matrix (independent) for this model will be used. In addition, the formula effect by visit will be summarized. An alpha level of 0.05 will be used to test the formula effect. 95% confidence intervals and p-values will be reported on the odds ratio to compare formula groups at each visit. The test

for treatment effect of the score for the gastrointestinal characteristic or general disposition over the 16-week period will have the following hypotheses:

H_0: Odds Ratio for formula groups = 1, no difference between the formula groups.

H_1: Odds Ratio for formula groups ≠ 1, there is a difference between the formula groups.

11.2.1. Stool composition

For stool composition, stool samples will be collected at the last study visit (visit 9, Day 120) to assess moisture content, total soap fatty acid concentrations, soap palmitic acid (calcium palmitate) concentrations, and calcium concentration. Stool composition values that are below the lower limit of detectability will be set to that lower limit value (for example, < 0.05 mg/g will be replaced with 0.05 ng/mL). Dry weights for each concentration will be summarized by formula group with descriptive statistics. Mean concentrations of the formula groups over the 16-week study will be analyzed as described in section 11.2.

11.2.2. Number of stools

Number of stools per day will be assessed and reported at each visit. The responses to the average number of bowel movements per day will be coded as: 0=0, 1=1, 2=2, 3=3, 4=4, 5=5, 6=6, 7=7, 8=8, 9=9, and 10=10+. The score for number of bowel movements over the 16-week study will be analyzed as described in section 11.2.

11.2.3. Stool consistency

Stool consistency in the past 24 hours will be assessed and reported at each visit. The responses to the consistency of the bowel movement will be coded as: 1=Hard, 2=Formed Soft, 3=Mushy Soft, 4=Runny Soft, 5=Watery. The score for stool consistency over the 16-week study will be analyzed as described in section 11.2.

11.2.4. Fussiness

Two separate questions for fussiness will be assessed and reported at each visit. Fussiness in the past 24 hours will be coded as: 0=Not at all fussy, 1=Slightly fussy, 2=Moderately fussy, 3=Very fussy, and 4=Extremely fussy. Over the past 24 hours, fussiness compared to normal will be coded as: 0=Less fussy than normal, 1=About the same level of fussiness as always, and 2=More fussy than normal. Each score for fussiness over the 16-week study will be analyzed as described in section 11.2.

11.2.5. Amount of gas

Two separate questions for gassiness, and amount of gas in the past 24 hours will be assessed and reported separately at each visit. Gas over the past 24 hours will be coded

as: 0=None at all, 1=Slight amount of gas, 2=Moderate amount of gas, and 3=Excessive amount of gas. Over the past 24 hours, gassiness compared to normal will be coded as: 0=Less gas than normal, 1=About the same amount of gas as normal, and 2=More gas than normal. Each score for gassiness over the 16-week study will be analyzed as described in section 11.2.

11.2.6. Infant Characteristics Questionnaire (ICQ)

The Infant Characteristics Questionnaire (ICQ) was administered at visits 1, 2, 3, 5, 7, and 9. The ICQ (Appendix A) was developed as a short, factor-analytic screening device for difficultness (Bates et al., 1979). The data from the 24-item questionnaire will be summarized into four subscales: Fussy-difficult, Unadaptable, Dull, and Unpredictable. Based on the examination of the factor structure of the items, the authors of the ICQ composed scales using only the discriminating items for use with their normative sample. These scales are composed as follows:

- Fussy/Difficult: Items 1, 5, 6, 13, 22, and 24
- Unadaptable: Items 9, 10, 11, and 20
- Dull: Items 15 (reverse-coded), 16, and 23
- Unpredictable: Items 2, 3, and 4

The sum of the responses for each of the items above comprise the score for each scale. A response for each item is required to calculate a sum score, otherwise the sum score will be set to missing. Higher scores indicate a more difficult temperament. ICQ scales will be summarized by study visits at study sites. Study visits are planned to occur every 15 or 30 (+/- 3) days. A window will be applied to each visit to ensure that subjects are compared at the same formula duration.

Analysis Visit	Target Day	Window
1	0	N/A
2	15	8 – 22
3	30	23 – 44
5	60	45 – 74
7	90	75 – 104
9	120	105 – 134

If more than 1 visit falls within a window, then the visit closest to the target day will be used in the analysis. If more than one visit falls within a window and they are the same distance to the target day, then the latest visit will be used in the analysis. Unscheduled assessments will be assigned to the associated visit based on date and time of assessment.

The scores of the four subscales will be summarized separately by formula group with descriptive statistics at each of the 6 visits. The test for treatment effect of each score over the 16-week study will have the following hypotheses:

H_0: There is no difference between the formula groups.

H_1: There is a difference between the formula groups.

Formula groups will be compared using a MMRM with sex, age at enrollment, baseline value, formula, pooled site, visit, formula by pooled site interaction, and formula by visit interaction as factors. The covariance matrix will be chosen based on the AIC criteria to choose the covariance matrix that best fits the model. In addition, the formula effect by visit will be summarized. An alpha level of 0.05 will be used to test the formula effect. 95% confidence intervals and p-values will be reported on the difference of least square means for comparison of formula groups at each visit.

The items which are included in the questionnaire but not included in scales above will be summarized individually with descriptive statistics by visit and formula group.

11.3. Physical Examination

Physical examination results will be listed by subject. No summary tables of physical examination are planned.

11.4. Prior and Concomitant Medications

At each study visit, site personnel will interview parent(s)/guardian(s) to obtain information about all concomitant therapy that was administered since the previous study visit. Concomitant medications include prescription medications, over-the-counter medications, and herbal supplements. This information will be recorded in the infant's medical record.

Any medications or therapy administered and discontinued before the first feeding of randomized formula will be considered as prior medications. Concomitant medications (medications present while consuming randomized formula during the study period) will be recorded throughout the study and at early discontinuation. Prior and concomitant medication verbatim terms captured via the EDC system will be mapped to Anatomical/Therapeutic/Chemical (ATC) class and Preferred Terms using the World Health Organization (WHO) Drug Dictionary, B3 September 2020 version.

Concomitant medications will be summarized for each formula group by WHO ATC class and preferred term. These summaries will present the number and percent of infants using each medication. Subjects may have more than one medication per ATC

class and preferred name. At each level of subject summarization, a subject is counted once if one or more medications at that level is reported for the subject. Each summary will be ordered by descending order of incidence of ATC class and preferred term.

Prior medications will be listed by subject.

12. Planned Tables, Figures, and Listings

A table of contents for the tables, listings, and figures will be presented in a separate document as the list of summaries or numbering may change after finalization of this document. If additional summaries are added that are not described in this document, then this SAP will be amended, or an addendum will be created.

13. References

Bates JE, Freeland CA, Lounsbury ML. Measurement of infant difficultness. Child development. 1979 Sep 1:794-803.

Nelson SE, Rogers RR, Ziegler EE, Fomon SJ: Gain in weight and length during early infancy. Early Human Development. 1989; 19 (4):223-239.)

• Appendix A: Infant Characteristics Questionnaire

On the following questions, please circle the number that is most typical of your baby. "About average" means how you think the typical baby would be scored. Put NA next to any item that does not apply to your baby. For example, if your baby has not had solid food yet, you would mark the item regarding his/her reaction to solid food as NA.

1. How easy or difficult is it for you to calm or soothe your baby when he/she is upset?

1	2	3	4	5	6	7
Very easy			About average			Difficult

2. How easy or difficult is it for you to predict when your baby will go to sleep and wake up?

1	2	3	4	5	6	7
Very easy			About average			Difficult

3. How easy or difficult is it for you to predict when your baby will become hungry?

1	2	3	4	5	6	7
Very easy			About average			Difficult

4. How easy or difficult is it for you to know what's bothering your baby when he/she cries or fusses?

1	2	3	4	5	6	7
Very easy			About average			Difficult

5. How many times per day, on the average, does your baby get fussy and irritable--for either short or long periods of time?

1	2	3	4	5	6	7

Never	1-2 times per day	3-4 times per day	5-6 times per day	7-9 times per day	10-14 times per day	More than 15

6. **How much does your baby cry and fuss in general?**

1	2	3	4	5	6	7
Very little; much less than the average baby		Average amount; about as much as the average baby				A lot; much more than the average baby

7. **How did your baby respond to his/her first bath?**

1	2	3	4	5	6	7
Very well--baby loved it		Neither liked nor disliked it				Terribly--didn't like it

8. **How did your baby respond to his/her first solid food?**

1	2	3	4	5	6	7
Very favorably--liked it immediately		Neither liked nor disliked it			Very negatively--did not like it at all	

9. **How does your baby typically respond to a new person?**

1	2	3	4	5	6	7
Almost always responds favorably		Responds favorably about half the time			Almost always responds negatively at first	

10. **How does your baby typically respond to being in a new place?**

1	2	3	4	5	6	7
Almost always responds favorably		Responds favorably about half the time			Almost always responds negatively at first	

11. **How well does your baby adapt to things (such as in items 7-10) eventually?**

1	2	3	4	5	6	7
Very well, always likes it eventually		Ends up liking it about half the time			Almost always dislikes it in the end	

12. **How easily does your infant get upset?**

1	2	3	4	5	6	7

Very hard to upset-- even by things that upset most babies	About average	Very easily upset by things that wouldn't bother most babies

13. When your baby gets upset (e.g., before feeding, during diapering, etc.), how vigorously or loudly does he/she cry and fuss?

1	2	3	4	5	6	7
Very mild intensity or loudness		Moderate intensity or loudness			Very loud or intense, really cuts loose	

14. How does your baby react when you are dressing him/her?

1	2	3	4	5	6	7
Very well-- likes it		About average--doesn't mind it			Doesn't like it at all	

15. How active is your baby in general?

1	2	3	4	5	6	7
Very calm and quiet		Average			Very active and vigorous	

16. How much does your baby smile and make happy sounds?

1	2	3	4	5	6	7
A great deal, much more than most infants		An average amount			Very little, much less than most infants	

17. What kind of mood is your baby generally in?

1	2	3	4	5	6	7
Very happy and cheerful		Neither serious nor cheerful				Serious

18. How much does your baby enjoy playing little games with you?

1	2	3	4	5	6	7
A great deal, really loves it		About average			Very little, doesn't like it very much	

19. How much does your baby want to be held?

1	2	3	4	5	6	7
Wants to be free most of the time		Sometimes wants to be held; sometimes not			A great deal-- wants to be held almost all of the time	

20. How does your baby respond to disruptions and changes in everyday routine, such as when you go to church or a meeting, on trips, etc.?

1	2	3	4	5	6	7
Very favorably, doesn't get upset		About average			Very unfavorably, gets quite upset	

21. How easy is it for you to predict when your baby will need a diaper change?

1	2	3	4	5	6	7
Very easy		About average			Very difficult	

22. How changeable is your baby's mood?

1	2	3	4	5	6	7
Changes seldom, and changes slowly when he/she does change		About average			Changes often and rapidly	

23. How excited does your baby become when people play with or talk to him/her?

1	2	3	4	5	6	7
Very excited		About average				Not at all

24. Please rate the overall degree of difficulty your baby would present for the average mother.

1	2	3	4	5	6	7
Super easy		Ordinary, some problems			Highly difficult to deal with	

25(A). On the average, how much attention does your baby require, other than for caregiving (feeding, diaper changes, etc.)?

1	2	3	4	5	6	7
Very little--much less than average		Average amount			A lot--much more than the average baby	

26(B). When left alone, your baby plays well by him/herself.

1	2	3	4	5	6	7
Almost always		About half the time			Almost never--won't play by self	

27(C). How does your baby react to being confined (as in a carseat, infant seat, playpen, etc.)?

1	2	3	4	5	6	7
Very well--likes it		Minds a little or protests once in awhile			Doesn't like it at all	

28(D). How much does your baby cuddle and snuggle when held?

1	2	3	4	5	6	7
A great deal--<br>almost every time		Average; sometimes does<br>and sometimes doesn't			Very little;<br>seldom cuddles	

Adobe Sign
