# Supplementary material for: New infant formulas for healthy term infants: A randomized, controlled, double-blind, multicenter, non-inferiority design safety study
Source: PLoS One. 2025 Dec 17;20(12):e0336689. doi: 10.1371/journal.pone.0336689 (PMC12711075; doi:10.1371/journal.pone.0336689)
Supplement: Attachment — Submitted filename: Response to Reviewers.docx [file pone.0336689.s006.docx]

The below indicates revisions made in response to reviewer/editor request, with our responses in red.

Formatting changes not highlighted

- Updated formats of headers, in-text citations, and references to tables and figures to match PLOS One templates and style guidelines.
- Removed Funding information from main text document

When submitting your revision, we need you to address these additional requirements.

1.Please ensure that your manuscript meets PLOS ONE's style requirements, including those for file naming. The PLOS ONE style templates can be found at

<https://journals.plos.org/plosone/s/file?id=wjVg/PLOSOne_formatting_sample_main_body.pdf> and

<https://journals.plos.org/plosone/s/file?id=ba62/PLOSOne_formatting_sample_title_authors_affiliations.pdf>

2. Please note that funding information should not appear in any section or other areas of your manuscript. We will only publish funding information present in the Funding Statement section of the online submission form. Please remove any funding-related text from the manuscript.

Verified.

3. Thank you for uploading your study's underlying data set. Unfortunately, the repository you have noted in your Data Availability statement does not qualify as an acceptable data repository according to PLOS's standards.

At this time, please upload the minimal data set necessary to replicate your study's findings to a stable, public repository (such as figshare or Dryad) and provide us with the relevant URLs, DOIs, or accession numbers that may be used to access these data. For a list of recommended repositories and additional information on PLOS standards for data deposition, please see <https://journals.plos.org/plosone/s/recommended-repositories>.

Editor: No changes have been made given our data is provided on Zenodo, which is listed on the linked page as an acceptable repository. The DOI is currently not public but will be made public if the manuscript is accepted. Please advise if further action is necessary.

4. Please include captions for your Supporting Information files at the end of your manuscript, and update any in-text citations to match accordingly. Please see our Supporting Information guidelines for more information: <http://journals.plos.org/plosone/s/supporting-information>.

Verified, however no captions were necessary.

5. If the reviewer comments include a recommendation to cite specific previously published works, please review and evaluate these publications to determine whether they are relevant and should be cited. There is no requirement to cite these works unless the editor has indicated otherwise.

No amendments necessary.

7. Please review your reference list to ensure that it is complete and correct. If you have cited papers that have been retracted, please include the rationale for doing so in the manuscript text, or remove these references and replace them with relevant current references. Any changes to the reference list should be mentioned in the rebuttal letter that accompanies your revised manuscript. If you need to cite a retracted article, indicate the article’s retracted status in the References list and also include a citation and full reference for the retraction notice.

[Note: HTML markup is below. Please do not edit.]

Reviewer's Responses to Questions

**Comments to the Author**

1. Is the manuscript technically sound, and do the data support the conclusions?

The manuscript must describe a technically sound piece of scientific research with data that supports the conclusions. Experiments must have been conducted rigorously, with appropriate controls, replication, and sample sizes. The conclusions must be drawn appropriately based on the data presented.

Reviewer #1: Yes

Reviewer #2: Yes

Reviewer #3: Partly

2. Has the statistical analysis been performed appropriately and rigorously?

Reviewer #1: I Don't Know

Reviewer #2: Yes

Reviewer #3: Yes

3. Have the authors made all data underlying the findings in their manuscript fully available?

The [PLOS Data policy](http://www.plosone.org/static/policies.action#sharing) requires authors to make all data underlying the findings described in their manuscript fully available without restriction, with rare exception (please refer to the Data Availability Statement in the manuscript PDF file). The data should be provided as part of the manuscript or its supporting information, or deposited to a public repository. For example, in addition to summary statistics, the data points behind means, medians and variance measures should be available. If there are restrictions on publicly sharing data—e.g. participant privacy or use of data from a third party—those must be specified.

Reviewer #1: No

Reviewer #2: Yes

Reviewer #3: Yes

Editor: We request the ability to retain raw data, made available upon request, and provide only the summary data used for tables and figures. This is due to 1) privacy and 2) data ownership. First, although de-identified, these data correspond to a sensitive human population (infants) and contain information that together could risk identification (sex, birth dates, ethnicities, medical histories, anthropometrics, locations of IRB approvals, etc.), and it is uncommon in the field of pediatric nutrition science to publish full raw datasets. Second, these data are owned by the funders, and requests would first need to be approved after request to corresponding author David Glover at dlgover@pbmcap.com.

4. Is the manuscript presented in an intelligible fashion and written in standard English?

PLOS ONE does not copyedit accepted manuscripts, so the language in submitted articles must be clear, correct, and unambiguous. Any typographical or grammatical errors should be corrected at revision, so please note any specific errors here.

Reviewer #1: Yes

Reviewer #2: Yes

Reviewer #3: Yes

5. Review Comments to the Author

Please use the space provided to explain your answers to the questions above. You may also include additional comments for the author, including concerns about dual publication, research ethics, or publication ethics. (Please upload your review as an attachment if it exceeds 20,000 characters)

Reviewer #1: subject
The purpose of the study was well stated and was well designed.

Suggested:

The study covers a 120day study and the long -term effects of this formula can provide more information that can be mentioned in the restrictions section,

Not given the geographical or ethnic diversity of infants and its effect on information results

We have expanded on the limitations of the study and what populations the results can be generalized to.

Reviewer #2: The complexity of a two-part trial requires careful explanation (which the authors have done, but in a clearer way is needed).
- A key limitation of this study is that both experimental formulas differed from the control on multiple components simultaneously. Because several compositional changes were tested together, it is not possible to determine which specific ingredient(s) were responsible for the observed improvements in tolerance (reduced gassiness/fussiness) or stool fatty acid profiles.

Addressed in the limitations.
- Another limitation is that genetic and parental influences on infant growth were not evaluated. Since anthropometric factors are strongly influenced by heredity, some observed differences in growth patterns may reflect underlying genetic variation rather than formula composition. Although baseline size was adjusted in statistical models, without genetic or parental growth data, residual confounding remains possible.

Addressed in the limitations.
- The experimental formulas differ in multiple components, making attribution of effects difficult. At the same time, the relatively small control groups (especially in Part 2 due to 8:1 randomization) reduce statistical precision. Together, these design features limit confidence in interpreting the observed differences.

Regarding statistical power, Part 2 included infants from Part 1 in the control group, comprising similar statistical power as in Part 1. Though ultimately, we agree that it would have been ideal to have had a complete separation between the two parts or conduct them at the same time.

- The study considered breastfeeding only in terms of duration prior to enrollment, but did not account for other maternal or contextual influences (such as maternal nutrition, health status, or socioeconomic conditions) that are known to affect infant growth through breast milk composition and feeding practices. It limits the ability to generalize findings to mixed-fed populations and to compare outcomes meaningfully against the full range of breastfeeding influences.

Addressed in the limitations. We also noted that results also do not generalize to other types of formulas (soy-based, hypoallergenic, extensively hydrolyzed, etc.) either.

- While this study demonstrates safety and non-inferiority of the new formulas compared to standard formula, future research should include a concurrent breastfed reference group. Breastfeeding is the gold standard for infant nutrition, and having an exclusively breastfed control would allow for more meaningful interpretation of growth and tolerance outcomes in the context of natural feeding. Such a comparison would also help clarify whether any observed differences between formulas approach, match, or diverge from the growth patterns of breastfed infants.

Addressed in the limitations. We made further comment in the discussion on what is expected to happen in breastfed infants based on existing meta-analyses.

Reviewer #3: Dear editor in chief,
This manuscript can be accepted. It was a nice and comprehensive article on formulas for infants. The results can shed light on this issue and can be used to be evaluated in further investigations.

6. PLOS authors have the option to publish the peer review history of their article ([what does this mean?](https://journals.plos.org/plosone/s/editorial-and-peer-review-process#loc-peer-review-history)). If published, this will include your full peer review and any attached files.

If you choose “no”, your identity will remain anonymous but your review may still be made public.

**Do you want your identity to be public for this peer review?** For information about this choice, including consent withdrawal, please see our [Privacy Policy](https://www.plos.org/privacy-policy).

Reviewer #1: No

Reviewer #2: **Yes:** Dr. Saeid Sadat mansouri

Reviewer #3: No
